# Kala-azar elimination in a highly-endemic district of Bihar, India: A success story

**DOI:** 10.1371/journal.pntd.0008254

**Published:** 2020-05-04

**Authors:** Vijay Kumar, Rakesh Mandal, Sushmita Das, Shreekant Kesari, Diwakar Singh Dinesh, Krishna Pandey, Vidyanand Rabi Das, Roshan Kamal Topno, Madan Prasad Sharma, Rudra Kumar Dasgupta, Pradeep Das

**Affiliations:** 1 Department of Vector Biology and Control, Rajendra Memorial Research Institute of Medical Sciences (ICMR), Agamkuan, Patna, Bihar, India; 2 Department of Microbiology, All India Institute of Medical Sciences, Patna, Bihar, India; 3 Department of Clinical Medicine and Treatment, Rajendra Memorial Research Institute of Medical Sciences (ICMR), Agamkuan, Patna, Bihar, India; 4 State Health Society of Bihar, Patna, Bihar, India; 5 National Vector Borne Disease Control Program, Sham Nath Marg, New Delhi, India; University of Liverpool, UNITED KINGDOM

## Abstract

**Background:**

Visceral leishmaniasis (VL) or Kala-azar has been a major public health problem in Bihar, India, for several decades. A few VL infected districts including Vaishali have reported >600 cases annually. Hence, in 2015, the Government of India entrusted ICMR-Rajendra Memorial Research Institute of Medical Sciences, Patna, to implement an integrated control strategy for achieving the VL elimination target (<1 case per 10,000 people at the block level) in the Vaishali District of Bihar.

**Methodology:**

This study was conducted between January 2015 and December 2016. An integrated control strategy including the spatio-temporal mapping of VL-case distribution, active case detection, chemical-based vector control using indoor residual spraying (IRS), community awareness campaigns, the training of IRS members, the training of medical doctors for effective treatment, daily monitoring and the supervision of IRS activities, logistic management, post-IRS quality assurance, epidemiological surveillance, and entomological monitoring was performed. An insecticide quantification test was performed for evaluating the IRS quality on sprayed walls. A modern compression pump was used to maintain spray quality on different wall surfaces. The impact of IRS was assessed through sand fly collection in human dwellings and cattle sheds in pre- and post-IRS. The insecticide susceptibility of local *P*. *argentipes* was performed before each IRS round (in February and June) during 2015–2016. Statistical analysis such as the mean, percentage, and 95% CI were used to summarize the results.

**Findings:**

All 16 blocks of the Vaishali District achieved the VL elimination target in 2016. The integrated VL control strategy helped reduce the number of VL cases from 664 in 2014 to 163 in 2016 and the number of endemic villages from 282 in 2014 to 142 in 2016. The case reduction rate was increased from 22.6% in 2014 to 58.8% in 2016. On average, 74 VL infected villages became Kala-azar free each year from 2015 to 2016.

**Conclusions:**

The results of this study suggest that the elimination of VL is possible from all endemic blocks of Bihar if the integrated Vaishali VL control strategy is applied under strong monitoring and supervision.

## Author summary

The World Health Organization (WHO) has set a target to eliminate visceral leishmaniasis (VL), commonly known as “Kala-azar,” as a public health problem in India by 2020. The elimination target is defined as achieving less than 1 case per 10,000 people at the block level. Although India has made substantial progress in the elimination of the disease since 2012, VL remains a stable public health problem in four middle-eastern states including Bihar. Bihar contributes >61% of the total Indian cases annually, and a few districts of the state have reported more than 600 cases annually. In this study, the results indicate that an intensive integrated VL control strategy including epidemiological analysis based on a geographical information system (GIS), hot-spot mapping, active case detection, vector control using the indoor residual spraying (IRS) of chemical insecticides, awareness campaigns, human resource development, the close monitoring of control activities, and active epidemiological surveillance and entomological monitoring can achieve the elimination target in the highly endemic region of Bihar. The elimination of VL from highly endemic zones is urgently required to control any new outbreak. Therefore, the implementation of the Vaishali VL control strategy is strongly recommended in all highly endemic districts of Bihar, India.

## Introduction

Visceral leishmaniasis (VL or Kala-azar) is a deadly tropical disease caused by the protozoan parasite genus *Leishmania*. The clinical manifestation of the disease is characterized by irregular bouts of fever, weight loss, splenomegaly, hepatomegaly, and anemia. Visceral leishmaniasis is endemic in >80 countries. However, 90% of the global cases are reported in six countries: Brazil, Ethiopia, India, Somalia, South Sudan, and Sudan [1]. In the Indian subcontinent (ISC), VL is pure anthroponotic and parasites are spread to humans by the bite of an infected female sand fly species *Phlebotomus argentipes* (Diptera: Psychodidae) [2]. Worldwide, an estimated 200,000–400,000 cases and 20,000–40,000 deaths occur annually [3]. The Indian subcontinent accounts for two-thirds of the total global cases, of which more than 50% occur in the State of Bihar, India. Moreover, 61.1% (i.e. 33 out of 54) of total VL affected Indian districts are from Bihar.

In India, the national VL control program was well-initiated in 1990–1991. In 2002, the Government of India launched a National Health Policy to eliminate VL from the region by 2010 [4, 5]. Later in 2005, under the umbrella of WHO, the governments of Bangladesh, India, and Nepal developed a strategic framework to eliminate VL as a public health problem by 2015 [4, 5]. The target year was initially postponed to 2017, but is now 2020 [1, 6]. Elimination is defined as reducing the annual incidence of VL to <1 case per 10,000 people at the sub-district/block level [6]. The main strategies for VL elimination are: (a) early case detection and complete treatment, (b) integrated vector management, (c) effective disease surveillance, (d) social mobilization and behavioral changes; and (e) operational research [4–6]. In India, indoor residual spraying (IRS) with dichlorodiphenyltrichloroethane (DDT) has been the mainstay for VL vector control since 1976 [1,7]. Many studies have been performed to assess the impact of IRS with other VL vector control methods to establish IRS as a single or combined control strategy [8–11]. In 2015, biannual DDT-based IRS was replaced by the synthetic pyrethroid alphacypermethrin (5%) due to resistant development in sand flies against DDT [12, 13].

The current spatial distribution of VL is mostly confined to a limited area in four middle-eastern Indian states (Bihar, Jharkhand, Uttar Pradesh, and West Bengal). Therefore, it is possible to control it and even achieve the elimination target in the country. The scientific feasibility of the elimination strategy is based on the epidemiological vulnerability and the effectiveness and feasibility of intervention. However, after several efforts taken from different government and non-governmental organizations, VL is still endemic in the 458 poorest blocks of Bihar. A few VL infected blocks have persistently remained highly endemic (i.e. ≥3 cases/10,000 people) for several years [5]. Moreover, the VL-vector control program in Bihar has suffered from several problems such as the improper implementation of control strategies, weak monitoring and supervision systems, lack of technological involvement, lack of awareness activities about the program, delay in the diagnosis and treatment of infected patients, and the injudicious application of IRS [12, 14, 15]. Therefore, the implementation of all control strategies in an effective way is paramount to achieve the VL elimination target in Bihar and in India overall.

Hence, in 2015, the Director General of Health Services (DGHS, New Delhi) and the National Vector Borne Disease Control Program (NVBDCP, New Delhi) assigned the Vaishali District (the second most VL endemic district in Bihar) to ICMR-Rajendra Memorial Research Institute of Medical Sciences (ICMR-RMRIMS; Patna) to reduce the VL incidence to below 1 case per 10,000 people in all infected blocks. This study demonstrates the impact of the control activities performed to reach the elimination target in the Vaishali District.

## Materials and methods

### Study area

**Fig 1:**
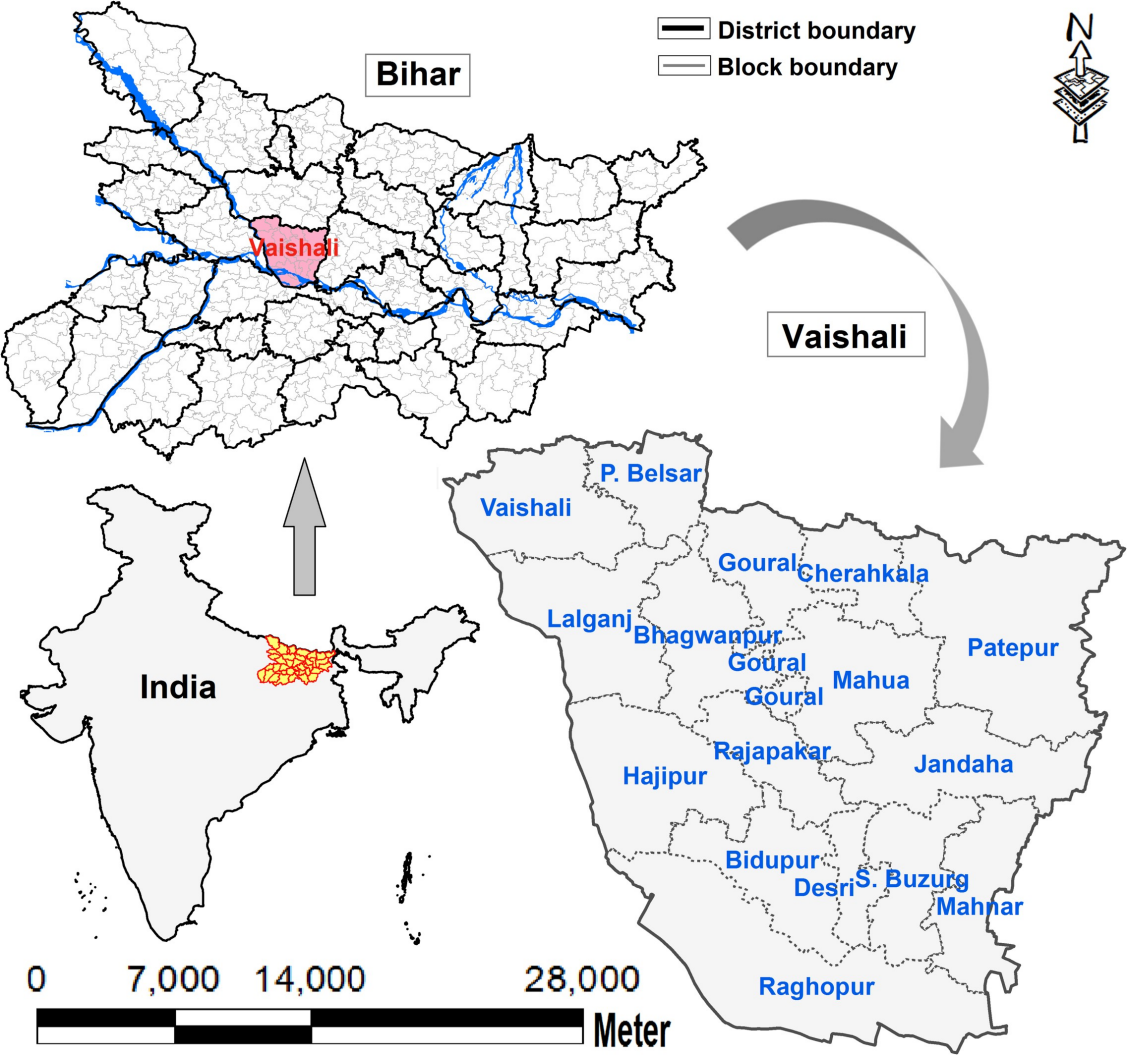
Map of the study area showed the location of Bihar in India and the location of Vaishali in Bihar. A GIS-database built in the remote sensing project of ICMR-Rajendra Memorial Research Institute of Medical Sciences was used to create the maps in the figure.

The Vaishali District of Bihar (25.6833°N, 85.2167° E) is bordered by the Muzaffarpur District in the north, the Ganga River and the Patna District in the south, the Samastipur District in the east, and the Gandak River and Saran District in the west (Fig 1). Vaishali extends over an area of 2,036 square kilometers with 16 community development (CD) blocks, 1,542 villages, and 65 wards. The total recorded population is 3.5 million with 0.61 million households [16]. A total of 2719 cases (average 906 cases/year) and two deaths were reported during 2012–2014 [17]. On average, 22% of the villages in the district were VL-affected each year.

### Epidemiological database generation and case surveillance

An epidemiological VL-case database [from the household level to the village, block, and district levels] was generated for 2012 to 2016. The annual line-list of the total VL and post-Kala-azar dermal leishmaniasis (PKDL) patients treated during the year were obtained from the District Vector Borne Disease Control Office (DVBDCO) in Hajipur, Vaishali. The line-list includes patient details such as name, age, gender, address, onset of fever in days, date of diagnosis and method, date of treatment, medicine used for treatment and total days, previous VL history (if there has been a relapse or reoccurrence), and post-treatment follow up. Each patient was then verified at their mentioned address and the geographic location (latitude and longitude) recorded using the global positioning system (GPS) device for mapping. Visceral leishmaniasis cases belonging to other districts and repeatedly mentioned in the annual line list were omitted from the final database, and the number of total cases was then summarized at the district, block, and village levels. The annual case incidence rate (CIR) per 10,000 people at the block level from 2012–2016 was calculated using the following Eq. [15]:

CIR=(a/b)x10,000

where a = the total number of new VL cases reported in the block annually, and b = the total number of persons at risk in the block annually. The population data of the year of the 2011 Indian census was used to calculate the block population 2012 and onwards based on the 2.5% mean annual population growth rate of the Vaishali District. The case reduction rate (CRR) for each year was calculated using the following Eq.:

CRR(%)=[(a‐b)/a]x100

where a = the total number of annual cases in the last year and b = the total number of annual cases in the target year.

### IRS village selection and action plan preparation

A GIS-based spatial mapping technique was employed for identifying and selecting the villages and the risk-prone households to include in the action plan for implementing a targeted IRS. The mapping of epidemiological data was done in two steps: (a) at the household (HH) level and (b) at the village level. The concept of village-level mapping was corroborated from our previous study, which dealt with the village endemicity analysis of VL in North Bihar using hot-spot mapping [15]. Based on the spatio-temporal trend of VL case dynamics in the Vaishali District, two types of villages were selected to receive IRS-based sand fly control intervention at the HH level. First, all the endemic villages having at least one VL case in a calendar year in the last three years (including the implementation year) were selected. Second, high-risk non-endemic villages (i.e. villages with no case report in the last three years) located along the periphery (S1 Appendix) of endemic villages in the last two years before the implementing year were selected. Furthermore, the peripheral villages of newly endemic villages during the first round of IRS were included in the action plan of the second round of IRS. Household level mapping was performed to identify the neighboring HHs within 500 m of a current index case location for focal spraying to interrupt disease transmission in the whole village (S1 Fig). The 500 m concept was adopted based on the highest possible flight range of sand flies in Bihar, India [18]. Household-level IRS was applied as soon as a village was reported with a new case. Focal spray was suspended if the report of a new case occurred within 12 weeks from the last date of IRS in the village (as per the duration of the effectiveness of SP reported in the WHO Pesticides Evaluation Scheme [PES] guidelines). The villages covered by focal spraying before the scheduled date mentioned in the action plan their remaining HHs were sprayed (by regular spraying) as per the date in the action plan. All GIS maps were prepared in ArcGIS v9.3 (ESRI, Redlands, CA, USA).

### Micro-action plan preparation and distribution to the squads for IRS implementation

A micro-action plan was developed for each IRS round. It included the names of revenue villages and related clinical sub-centers, panchayats and blocks, the total VL cases in the last three years including the implementing year, population, households, rooms, the number of squads and the total amount of insecticides required to cover the village population, the total days, the date of spray, and the names of the owners of the starting and the ending HHs for each squad per day. The squads were named in alphabetical order (i.e. A, B, C. . . . . . H) and identified with the villages allotted to them. The spray date was mentioned in one row in the micro-action plan. The final micro-action plan was supplied to the DVBDCO for scrutiny, modification if required, and distribution among the IRS squads.

### IRS rounds and insecticides used

This study was conducted between 2015 and 2016. Two rounds of IRS were performed annually using 166 IRS squads (S1 Table). All IRS activities (i.e. the recruitment process and team wise grouping of squad members, distribution and work allotment of spray teams at the block level, procurement and allotment of spraying logistics like the spray machine, buckets, measuring mugs, measuring cylinders, polythene sheets, pens, papers, registers, personal protection equipment or PPE (i.e. full sleeved shirt and pants, boots, helmets with visor, goggles, gloves, and masks), and the storing, distribution, and dispatch of insecticides at the block level and then to each team were implemented and performed by the State Government of Bihar. Standard protocol was explained to all squad members for the proper use of PPE during spraying. In 2015, the first round of IRS was performed using DDT (WP 50% at the dosage of 1g/m^2^). In the second round, DDT was used for the first 15 days then SP (alphacypermethrin 5% at 25mg/m^2^) was introduced due to an extended reporting of high-level (i.e. ≤50% mortality rate) resistance of DDT in *P*. *argentipes* [12, 15]. From that point on, SP was continued for VL vector control in Vaishali using IRS. In the switchover to SP from DDT, the efficacy of the alphacypermethrin dosage (i.e. 0.05 as recommended by WHO) was evaluated for sand fly control in Bihar through a collaborative study between RMRIMS, Patna, and LSTM, Liverpool. As the dosage was found effective for sand fly control, it has been adopted by the national VL vector control program by the NVBDCP, New Delhi, India [19].

### Training, demonstration, and human resource development

Four levels of staff training were conducted in each block: (a) the training of doctors, (b) the training of supervisors, (c) the training of monitors, and (d) the training of IRS operators. Indoor residual spraying training courses were conducted in accordance with the WHO/ Tropical Disease Research (TDR) *Manual for Indoor Residual Spraying* [20]. Training was performed in two rounds annually 5–7 days before each IRS round. However, new staff recruited during the program were trained immediately. Medical training for two days each in 6 primary health centers (PHCs) in 6 blocks were conducted for doctors concerning treatment with single-dose liposomal amphotericin B. Four of the PHCs were pre-established (i.e. before 2015) and two were newly established (i.e. from 2015) treatment centers upgraded for the prompt treatment of new VL cases at the sub-district level. The location of the treatment centers and the PHCs (namely Hajipur, Mahnar, Mahua, Raghopur, Patepur, and Vaishali) were selected based on population density, VL endemicity, road network, and proximity of villages around the PHCs. Supervisors were trained regarding supervising and monitoring the work at the block level: they included the staff from RMRIMS, the Kala-azar technical supervisors (KTSs), and the IRS Camp in Charge supervisor employed by the State Health Society of Bihar (SHSB). The monitors were especially trained as IRS monitors at the squad level. They were also provided knowledge regarding active case searching between social communities. The monitoring group included squad level field staff (hired on daily wages with minimum qualifications - 12^th^ grade passes in any subject), lab technicians from RMRIMS, and accredited social health activists (ASHAs) engaged by the SHSB. All IRS squad members (each squad consisted of one superior field worker and five field workers) were trained as IRS operators at the village and HH levels. The main purposes of their training were to achieve the proper use of spray pumps, the judicious application of insecticide (i.e. quality control: suspension preparation, spray timing, moving of pumps, and vertical surface coverage), and the better acceptance of IRS among social communities.

### Multi-level supervision and monitoring systems

**Fig 2:**
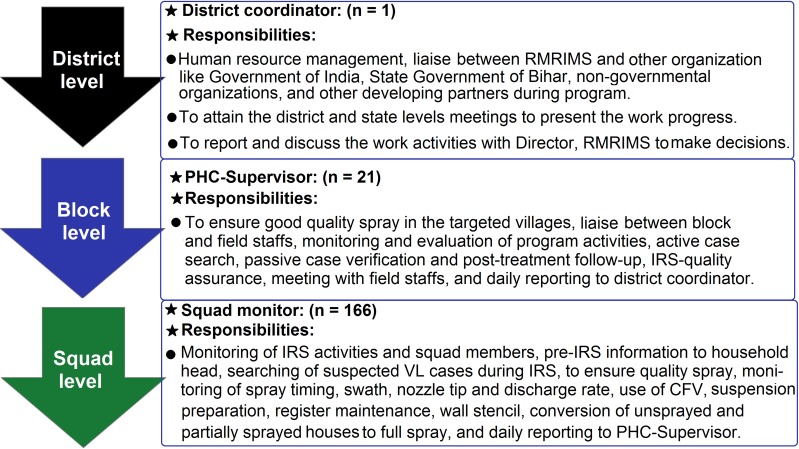
The three-tier monitoring and supervision system adopted by RMRIMS for IRS-based VL vector control program in the Vaishali District, Bihar, India.

An active monitoring and supervision system was developed for effective and smooth execution of all activities under the control program. Monitoring and supervision strategies were implemented at the three levels of government (i.e. the district to the block and village levels). One district coordinator, 21 PHC supervisors (1 per 8 squads), and 166 squads monitors (1 per each squad) were appointed in the IRS program. The nature of work and responsibilities assigned to each level of staff is summarized and illustrated in Fig 2. The hierarchy of program staff involved in the IRS work is shown in S2 Fig. Apart from RMRIMS and the SHSB, other organizations that participated in monitoring and evaluating the program activities were the World Health Organization (WHO), the National Vector Borne Disease Control Program (NVBDCP), and CARE India.

### Creating mass awareness and estimating perception for intervened activities

For creating mass awareness and circulating the needful information regarding Kala-azar, its sign and symptoms, mode of transmission, its lethal impact affecting public health, precautionary measures for complete treatment ensuring individual relief, and approaches for disease control via vector control particularly by IRS among the different communities of targeted villages, Information Education and Communication (IEC) was performed at zonal offices, club, schools, and community levels in different blocks. Behavior change communication (BCC) activities such as small media (i.e. handbills, posters, stickers, leaflets, and pamphlets), mass media (i.e. banners, campaign, audio tapes, and local arts), and training and demonstration (in communication skills) were performed. Outdoor media materials such as posters, stickers, handbills, and banners were used to inform the public about the signs and symptoms of VL and PKDL cases, its diagnosis and treatment, sand fly breeding and resting places, vector control through IRS and its methods, and post-IRS cautions. A proclamation was issued by RMRIMS two days before each IRS round to announce the date and time of upcoming IRS in the targeted villages (through microphone announcements, ASHAs, and social mobilization). The proclamation was made repeatedly on the same day. The types and quantity of IEC/BCC materials developed and distributed during IRS in 2015–2016 are visualized and summarized in the supplementary documents (S2 Table and S2 Appendix). A population survey (at pre- and post-IRS) was conducted to assess the effect of IEC, BCC, and prior information about IRS among social communities. In total, 16 villages (8 endemic and 8 non-endemic) each from 16 PHCs and 30 HHs in each village were selected randomly for surveying the awareness created by IEC, BCC, and prior information. Moreover, all the febrile cases that came to the treatment centers seeking VL treatment were interviewed and the amount of information they knew about VL and its symptoms were assessed.

### Implementation and standardization of compression pump

For improving the IRS quality and HH coverage, the conventional stirrup pump (CSP) was replaced with a modern hand compression pump (HCP) due to its easy operation and better efficacy in terms of coverage quality, handling, and user friendliness [21]. In the first round of IRS in 2015, all blocks in the Vaishali District were divided into two groups: 9 blocks by CSP (non-branded) and 7 blocks by HCP (produced and marketed by ASPEE Marut). All spray operators were provided with pre-IRS training regarding the implementation of proper spraying (the distance of the nozzle from the wall and the appropriate speed of spraying), the correct doses of insecticide (the ratio between insecticide and water), and proper solution preparation (the correct measure of water and insecticide, and its mixing). Post-IRS (1 day) HH spray quality and the operational feasibility of both pumps were assessed and compared for scaling up the use of the hand compression pump in 9 remaining blocks. The 50 sprayed villages (comprising 25 endemic and 25 non-endemic villages) each from CSP and HCP block groups were selected for the HH survey. In each village, 30 sprayed HHs were visited and examined for IRS quality assessment. In the meantime, total 200 spraymen were interviewed to assess the operational acceptability of both pumps for IRS.

### Evaluation of DDT-IRS

Insecticide Quantification Kit (IQK) based IRS quality assessment was performed for facilitating timely decision making and response towards the poor quality of insecticide application, maximizing the benefits from vector control, and minimizing the risks to public health and the environment [12, 22, 23]. Residual DDT was sampled from all 16 PHCs in the Vaishali District during the first round of IRS (February–July) in 2015. In each block, 3 villages sprayed by the 3 different squads were assessed. In total, 48 villages sprayed by 48 different squads were selected for the IQK test in the first round of DDT-IRS 2015. Indoor residual spraying samples were collected from every tenth house in a sequence of 60 sprayed houses in a village (6HHs/village). To avoid bias in quality assessment, samples were collected from three types of HHs: (a) mud wall, (b) brick wall, and (c) cemented plastered wall. Adhesive (2x5) cm^2^ Bostik discs (Bostik, Leicester, UK) and Sellotape (10 cm^2^) were used for extracting the DDT samples from the sprayed walls. Samples were scratched from different heights (i.e. 0.5, 0.9, 1.4, and 1.7 m) along four wall sides in a room (one scratch from each wall). All samples were collected between 0 and 1 day post spraying. A total of 1,152 wall samples were collected and tested for evaluating the DDT-IRS quantity on the wall using an IQK kit in the laboratory based on standard guidelines [12, 22, 23]. Any undersprayed HHs recorded with <0.8g/m^2^ DDT deposition on the walls were re-sprayed [12].

### Susceptibility test

Insecticide susceptibility tests were carried out for local *P*. *argentipes* against DDT (at 4% dosage) and SP (at 0.05% dosage) 7 days before the start of each IRS round during 2015 and 2016. The tests were conducted using ready-to-use insecticide impregnated paper based on WHO guidelines [24, 25]. Four replicates for each insecticide were performed. For each test, 18–25 *P*. *argentipes* were taken. Two replicate tests of 20–25 sand flies for control were also performed parallel to each test. For each test, the sand flies were exposed for 1h to the insecticide impregnated and control papers. The percent of mortalities was calculated by scoring the dead and alive sand flies after 24h of the recovery period. The mortality of the test sand flies was mathematically corrected by Abbott’s formula, and the control mortality range within 5–20% was only expressed as the corrected percent mortality [25, 26].

### Pre- and post-IRS sand fly density assessment

Local *P*. *argentipes* densities were monitored at pre- and post-IRS weeks using miniature CDC light traps installed in human dwellings and animal shelters at 18.00 hours and 06.00 hours. Light traps were placed near the corner of the bedroom/cattle shed in a house (0.2 m above the ground and 0.03 m away from the wall) [20, 26]. Pre-IRS sand fly densities were collected 7 days before the IRS intervention as baseline data at the selected HHs. Post-IRS sand fly densities were monitored at 15 days, 1 month, and 3 month intervals for evaluating the effect of IRS intervention in the study houses. For evaluating the IRS intervention effect, two groups of HHs were selected for entomological assessment. The first group contained intervention HHs, which were HHs selected from an endemic village where IRS was done with DDT/SP for sand fly control. All rooms in the HHs in this group were fully sprayed up to 1.8 m height. The second group contained control HHs, which were selected from a non-endemic village where IRS was not performed due to the non-endemicity of VL.

One village each from the intervention and control sites were randomly selected at the block level. In each village, 2 HHs from each group of 3 HH-types (mud, brick, and cemented plastered walls) were randomly selected for entomological collection. In total, 192 HHs were selected for sand fly collection in each IRS round. All insects collected in the light traps were labelled with HH identification, the village name, and the date of collection. Then the insects were brought to the RMRIMS laboratory, Patna, for sorting and further analysis. *P*. *argentipes* sand flies were separated from the other species and insects using morphological characteristics, and they were kept carefully in separate vials for density estimation. The percent of sand fly count change (SFC) was calculated using the following Eq. [26, 27]:

%ofchange(R)=[(B‐A)‐(D‐C)/A]x100

where A = the baseline mean SFC for the intervention HHs; B = the post-IRS mean SFC for the intervention HHs, C = the baseline mean SFC for the control HHs, and D = post-IRS the mean SFC for the control/sentinel HHs. Negative and positive ‘R’ values were associated with decrease and increase in SFC post-IRS, respectively.

### IRS data collection and coverage estimation

The household-level IRS status of each village was documented after spraying. The spray status such as partially sprayed, fully sprayed, and refused HHs were recorded. The total population, number of households, and villages sprayed against the targeted number were estimated for each IRS round. A post-IRS (within 2 days) house-to-house field survey was conducted based on WHO guidelines for the assessment of IRS quality (i.e. uniformly sprayed, patchy sprayed, and coverage of spray on the wall up to 1.8 m height), the status of the wall stencil (i.e. false stenciling and doubling in HH number), the post-IRS wall status in sprayed HHs (if washed out or repainted), and to determine if any HHs had missed out during IRS [20]. In each round, 7,200 HHs (30 HHs randomly selected in each village) from 240 villages (15 villages randomly selected) in 16 blocks were surveyed for the assessment. The definitions of the different spray status, wall stenciling, and missed HHs are given in the supplementary document (S3 Table).

### Daily monitoring and logistic support

For accurate implementation and smooth execution of all IRS activities to ensure good quality [i.e. suspension preparation including insecticide dosage, nozzle discharge rate, and length of swath on wall, and the proper use and performance of the control flow valve (CFV)], quality control monitoring was performed for each IRS squad as per WHO/TDR guidelines [20, 28]. When deficiencies were found, immediate corrective measures (i.e. guidelines, demonstration, and training for appropriate solution preparation and spraying and the replacement/supply of related spray instruments) were taken to improve the IRS quality. Any pumps and other related instruments (i.e. buckets, insecticide, water measuring mugs, measuring cylinders, nozzle tips, and the muslin cloth used to strain solid materials from the insecticide solution) found damaged, faulty, or missing by the spray squads during IRS were replaced immediately [28]. At the grass-roots level, superior field workers (SFWs) and squad monitors were observed and identified the problems. Final corrective measures such as the replacement of damaged and faulty pumps or instruments and the supply of lost/missed instruments and parts were made by the Camp in Charge supervisor and PHC supervisor deputed to each block. Faulty nozzle tips and CFVs were identified based on the discharge rate. Nozzle tips that produced below or above the standard discharge rate (i.e. 550±10 ml/m for a nozzle tip with CFV and 700±50 ml/m for a nozzle tip without CFV) were changed with a new one [28]. However, nozzle tips and CFVs found jammed with the spray substance or any other solid particles were cleaned using normal water. Jammed nozzle tips were cleaned using an unused toothbrush, while jammed CFVs were submerged in lukewarm water and left overnight for cleaning.

### Active case detection and prompt treatment

A door-to-door active case search was conducted in all endemic villages reported in the last year and in the new endemic villages reported in the implementation year. Two rounds (i.e. February-March and June-July adjusted with two annual peaks of VL cases in Vaishali) of the active case detection survey were conducted annually [29]. The staff involved in active case detection (ACD) activities were trained lab technicians and squad monitors from RMRIMS and accredited social health activists (ASHAs) from SHSB. They reached out to each HH before the start of each round. One ASHA covered 200 households or approximately 1,000 people. Moreover, all SFWs were trained in collecting febrile case information when they visited HHs during IRS. All household members appearing with a prolonged fever history (i.e. ≥14 days) associated with clinical splenomegaly and weight loss were screened with the 39-aminoacid–recombinant kinesin antigen (rK39) rapid diagnostic test [5]. Patients with a positive rK39 test were referred to the nearest health center for treatment with subsequent follow up at home [28]. Moreover, patients with a history of VL treatment who were suspected for PKDL-like skin lesions were also tested with rK39, and cases with positive results were referred for confirmation of diagnosis and treatment. However, patients with low hemoglobin, multiple infections, and other serious conditions were referred to RMRIMS for expert treatment [28]. All VL treated patients were followed up for 6 months post-treatment, and all PKDL patients were tracked until they received the full course of PKDL treatment.

### Data storage and statistical analysis

A joint database was developed for storage and analysis of epidemiological, entomological, and IRS-related data (the coverage of population, HHs, and villages) collected from the study area during 2015–2016. All data were stored on an Excel spreadsheet using Microsoft Office (2007). The descriptive statistics such as mean, minimum and maximum range, standard error (SE), confidence intervals (CI), and percentages were used to summarize the data. The t-statistic was used to calculate the sand fly density variation in IRS and non-IRS villages. The significance of all statistical tests was set at 0.05. All statistical analysis and graph preparations (i.e. bar and line graphs) were performed using Excel 2007 and SPSS 21.0 software.

### Ethics statement

The ICMR-Rajendra Memorial Research Institute of Medical Sciences Ethical Review Committee (affiliated with the Ministry of Health and Family Welfare, Government of India) approved the study. Informed written consent from adults and assent from children between 11 and 17 years old were obtained for their participation. For children below 11 years old, consent from a parent or local guardian was also obtained for their participation in the study. Villagers’ households were accessed in the study only after written informed consent was obtained. The data was analyzed anonymously; hence, no patient/person was identified by name.

## Results

### Village selection and IRS-coverage estimation

During 2015–2016, a total of 4 rounds of IRS were performed. The details of the IRS villages are summarized in the supplementary document (S4 Table). On average, 1,046.8 villages were targeted for IRS in each round; which represents 67.9% of the total revenue villages in the Vaishali District. An average 624.5 endemic and 422.3 non-endemic villages were targeted for spraying in each IRS round, and all villages were fully covered within an average of 58.4 days of spray. S5 Table shows the target and coverage details of spray households and population data with the percentage of locked HHs, HHs that refused IRS, and partially-sprayed and fully-sprayed HHs. In each round, an average 3,077,683 people (84.8% of the total population in Vaishali) and 521,634 HHs (82.2% of the total HHs in Vaishali) were targeted for spraying; of which 2,998,788 people (97.4%) and 504,356 (96.7%) HHs were covered during IRS. On average, 17,252 (3.3%) of the targeted households remained unprotected in each IRS round during 2015–2016. Of these, 9,986 or 1.9% HHs remained unprotected due to being locked while 7,266 or 1.4% HHs refused spraying. The percent of fully sprayed HHs increased from 90.8% in the first round of IRS in 2015 to 96.2% in the second round of IRS in 2016 (the average being 94.1% in all IRS rounds during 2015–2016). The percent of locked and refused HHs numbers gradually decreased from 2.7 and 2.4, respectively, in the first round of IRS in 2015 to 0.9% for both in the second round of IRS in 2016. On average, there were 1.4% and 1.9% refused and locked HHs, respectively, in all IRS rounds during 2015–2016.

### Focal spraying for VL transmission interruption

In total, 93 villages that were reported with new VL cases were targeted for focal spraying (FS) during 2015–2016. Of the 93 villages, 81 (87.1% of total targeted villages; 40.5 villages per year) were covered through FS (S6 Table). In each FS attempt, an average of 63.9 HHs (an average population of 355.6) was sprayed 500 m around a new case location. Around 12 out of 81 (i.e. 28.1%) FS villages reported a new VL case at the end of the year. In total, 12 villages (i.e. 6 villages/year) remained uncovered through FS during the study period; almost 9 (4.5 of 6 villages/year or 75%) reported a new VL case at the end of the year.

### Medical training and fast-treatment at block level health centers and other centers

In total, 14 medical doctors were trained in the 6 primary health centers (PHCs) at the block level for the treatment of new VL cases with liposomal amphotericin B single dose 10mg/kg. In total, 244 new VL cases (42.5% of the total 574 cases; 167 in 2015 and 77 in 2016) were treated during 2015–2016. Treatment of all VL-confirmed patients was completed within one day of the diagnosis test. During the study period, the 82 rK39 positive febrile cases diagnosed as VL with multiple problems (i.e. lack of clinical symptoms, typhoid, jaundice, anemia, and low hemoglobin) were referred to the Sadar Hospital, Hazipur, and RMRIMS, Patna, for expert treatment.

### Human resource development and IRS-improvement

A total of 21 PHC supervisors, 4 KTSs, 16 Camp in Charge supervisor, 166 squad monitors, 2431 ASHAs, 166 SFWs, and 830 field workers (FWs) were trained during the study period. S7 Table shows the contribution of squad monitors, ASHAs, KTSs, and PHC supervisors in refusal breaking during IRS in 2015–2016. Of the total HHs that refused IRS, 97.7% were converted to sprayed status per IRS round. The conversion rate of sprayed HHs from HHs that refused was highest among the squad monitors (54%) followed by the PHC supervisors (23.4%), ASHAs (15.9%), and KTSs (6.7%). Mainly, the ASHAs and squad monitors were responsible for refusal breaking at the grass-roots level. Many hard and tough HHs that refused IRS and were left unsprayed by ASHAs and squad monitors were converted to spray HHs by PHC supervisors and KTSs. The results of the post-IRS HH level survey of SFW and FW work activities are analyzed and summarized in the supplementary document (S8 Table). The results show 0.4% of the total surveyed HHs was recorded with false stenciling per IRS round; of which 76% partially sprayed HHs were stenciled as fully sprayed and 24% refused HHs were stenciled as partially/fully sprayed. False stenciling activities were recorded at zero percent in the second round of IRS in 2016. Overall, 0.2% HH wall stencils (per round) were recorded with multiple/double stencils; which was gradually decreased from 0.4% in the first round IRS in 2015 to 0.03% in the second round IRS in 2016. Similarly, the incidents of washed out/repainted HH numbers, patchy sprayed HH numbers, <1.8 m sprayed HH numbers, and missed HH numbers were greatly decreased from 0.5%, 21.9%, 8.4%, and 3.3% in the first round of IRS in 2015 to 0%, 3.2%, 0.2% and 0% in the second round of IRS in 2016, respectively (S8 Table). In each IRS round, an average of 92.1% (range: 78.1–97.4%) and 97.1% (range: 91.6–99.8%) of the total surveyed HHs were recorded as being uniformly sprayed and the walls were sprayed up to 1.8 m height, respectively. Incidents of patchy sprayed and HHs sprayed less than 1.8 m height were gradually decreased from the first round in 2015 to the second round in 2016.

### Logistic management and IRS quality improvement

The logistic equipment supplied for IRS quality improvement are summarized in the supplementary document (S9 Table). A total of 819 pumps were newly provided during the IRS program in 2015–2016; of which 723 were HCPs (225 in the first round of IRS in 2015 and 498 in the first round of IRS in 2016) and 96 were CSPs (47 in the first round of IRS in 2015 and 49 in the second round of IRS in 2015). The total damaged equipment replaced were 295 buckets, 179 insecticide measuring mugs, 170 water measuring mugs, 173 gloves, and 130 measuring cylinders. The total missing or faulty nozzle tips and washers newly provided or repaired was 269 and 77, respectively. In addition, damaged Markin clothes were changed with fresh ones 483 times. Also, 199 jammed CFVs were cleaned during the four IRS rounds.

### IRS improvement through IEC, BCC, and other pre-IRS activities

The impact of IEC, BCC, and pre-IRS activities on community awareness development about VL and its vector and control related activities are assessed and summarized in the supplementary document (S10 Table). A wide change in awareness development was observed before and after the IEC, BCC, and pre-IRS activities in all IRS rounds. The average percent of HHs aware of VL and PKDL cases and its symptoms, aware of treatment facilities at the government hospitals, and with knowledge of the VL vector, its breeding and resting sites, taking control of VL infection, and precautionary activities were increased from 53.5%, 59.1%, 46.1%, 59.8%, and 46.2% in the pre-activity period to 77%, 84.1%, 75.8%, 69%, and 51.7% in the post activity period, respectively. The details of HHs receiving prior information about IRS through different sources and practicing the post-IRS activities are provided in the supplementary document (S11 Table). The results show that an average of 99.9% of the total sampled HHs received prior information about IRS during the study period. The majority of the HHs (average 64.3%) were reported to receive pre-IRS information through microphone announcements and ASHAs both, followed by 24.9% through microphone announcements only and 9.7% through ASHAs only. Households receiving pre-IRS information through both microphone announcements and ASHAs considerably increased through the first round of IRS in 2015 to the second round of IRS in 2016. Only 0.8% of the total number of sampled HHs received IRS information through handbills, posters, banners, and neighbors. In each IRS round, 0.8% of the total targeted HHs denied receiving IRS after pre-information during 2015–2016. On average, 66.6% of the total sampled HHs were observed to practice post-IRS activities such as not washing the sprayed walls, wiping the floor after spraying, and waiting the room a minimum 30 min after IRS. Rapid improvement was observed in the practice of post-IRS activities from the first round of IRS in 2015 through the second round of IRS in 2016 (S11 Table).

### Impact of IEC, BCC and other pre-IRS activities on VL treatment awareness

During 2015–2016, a total of 143 febrile cases (59 in 2015, and 84 in 2016) visited government hospitals (at the block and district levels) to avail themselves of diagnosis and treatment of VL due to information provided by IEC, BCC, social mobilization, and microphone announcements. The 19 of 59 febrile cases (i.e. 32.2%) in 2015 and 7 of 84 (8.3%) in 2016 had a history of local public health clinic (non-governmental) visits (single/multiple times). Twenty-three of these patients were initially treated for seasonal flu and 3 were treated for typhoid. None of the 26 patients were tested for VL infection. In total, 6 out of 143 febrile cases (4.2%; 2 of the 6 were previously treated as typhoid) tested positive after taking the rK39 based VL confirmatory test at the block hospital. Five rK39 positive patients appeared with clinical symptoms (i.e. more than 15 days of fever, pallor and weakness, appetite loss, splenomegaly, and hepatomegaly) and were treated at the block/district hospitals and one asymptomatic patient rK39 positive with <15 days fever was referred to RMRIMS for further confirmation using bone marrow and spleen puncture tests. The patient was detected as VL positive with the bone marrow test (i.e. level 1+; 1–10 parasites/1000 oil immersion field), and he was treated at RMRIMS under expert observation [30, 31]. All positive VL patients were treated using single dose liposomal amphotericin B (10mg/kg as per the NVBDCP treatment guidelines) [28]. No relapse case was observed within 6 months post-treatment follow up.

### Implementation and standardization of hand compression pumps for IRS

In the first round of IRS in 2015, 150 conventional stirrup pumps or CSPs (2 pumps per squad) were replaced with 225 hand compression pumps or HCPs (distributed at a ratio of 3 pumps per squad) in 7 blocks (75 squads): Bidupur, Desri, Sahdei Bujurg, Hajipur, Jandaha, Raghopur, and Rajapakar. Newly provided HCPs were calibrated for IRS with DDT. All squad members were trained accordingly. The calibration details of HCPs compared to CSPs are described in the supplementary document (S12 Table).

### IQK-based post-IRS DDT quantification on walls and decision making

**Fig 3:**
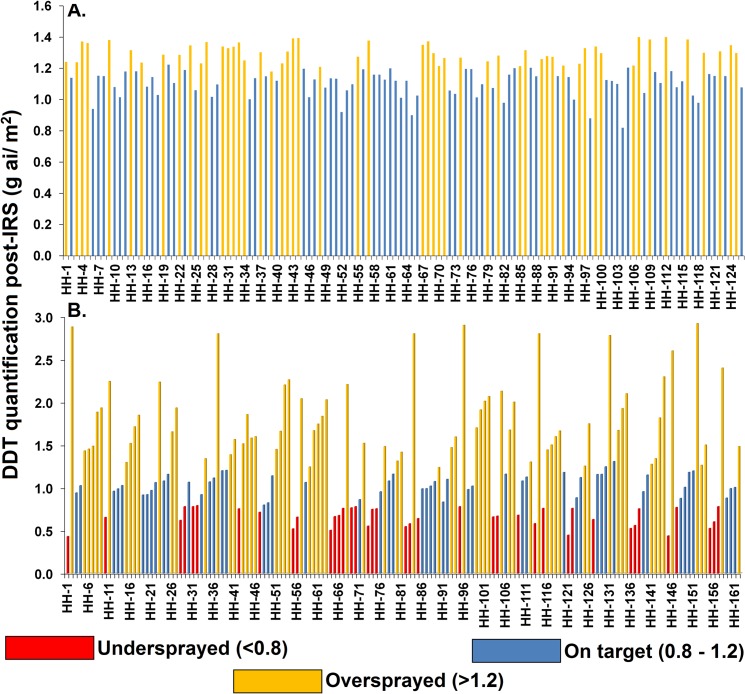
IQK-based DDT quantification post-IRS HHs sprayed by HCP (Panel A) and CSP (Panel B) assessed for undersprayed, on target, and oversprayed in first round of IRS in 2015 in the Vaishali District, Bihar.

In total, 288 HHs (126 HHs sprayed by HCP; 162 HHs sprayed by CSP) were assessed for DDT post-IRS deposition on walls using the IQK test resulting in 1152 samples (504 from HCP-sprayed walls and 648 from CSP-sprayed walls). The average quantity of DDT recovered on walls post-IRS was 1.25 g ai/m^2^ (95% CI: 0.35–2.90). The recovered quantity varied between pumps. For the HCP-sprayed walls, the average quantity recorded was 1.18 g ai/m^2^ (95% CI: 0.74–1.42). While on CSP-sprayed walls, the average quantity observed was 1.31 g ai/m^2^ (95% CI: 0.35–3.2). In total, in HCP sampled HHs, 1.4% of walls (7 of 504) were undersprayed (<0.8 g ai/m^2^), 54.8% of walls (276 of 504) were within the target range (0.8–1.2 g ai/m^2^), and 43.8% of walls were oversprayed (>1.2 g ai/m^2^). In the case of CSP sampled HHs, the percentage of undersprayed, on target range, and oversprayed HHs observed were 20.5, 29.9, and 49.5%, respectively. The average HH result for HCP showed 74 of 126 (58.7%) houses on target, and 52 of 126 (41.3%) oversprayed houses (Fig 3A). No (0%) house was undersprayed with HCP. For CSP, the average HH result showed 24.1% (39 of 162) houses undersprayed, 32.1% (52 of 162) houses on target, and 43.8% (71 of 162) houses oversprayed (Fig 3B). In total, 39 of 288 sampled HHs (13.5%) were re-sprayed during the first round of IRS in 2015.

### Assessment of HCP and CSP for IRS-intervention

Household-level (n = 1,500) spray quality among the 100 intervention villages (50 villages each for HCP and CSP) were assessed during the first round of IRS in 2015 (S13 Table). The 57.5% (862 of 1,500) of the CSP sprayed HHs showed evidence of a patchy spray mark on wall. The CSP based IRS-method (i.e. the entry of two persons at a time in the room for spraying, one for pumping and another for operating the spray-work, and insufficient space inside the rooms for conducting the CSP based IRS process which caused irritation among householders and resulted in partial coverage of walls in the house) was the reason for incomplete spraying in 50.1% (367 of 577) of total partially sprayed HHs. The operational acceptability of both pumps for IRS were surveyed among 200 spraymen during the study period. The majority of the spraymen (83%; 166 of 200) were comfortable with HCPs followed by CSPs (11.5%; 23 of 200). The 11 (5.5%) spraymen were comfortable for IRS with both pumps. No one was uncomfortable for IRS with any of the two pumps. Based on this result along with IQK-status, in the first round of 2016 and onwards, IRS in all 16 blocks was fully operated by HCP. In 2016, the previously used 225 HCPs in 7 blocks and 82 CSPs in remaining 9 blocks (total 91 squads; 3 HCPs to each squad) were replaced with 498 standard HCPs (manufactured and marketed by Hudson). The spraying characteristics of the standard HCPs were same as those of the previous HCPs used in the IRS rounds in 2015 (S12 Table).

### Active case detection and prompt treatment

During 2015–2016, an average of 1,55,712.3 people from 30,740.5 HHs over 273.3 villages were screened for active case detection (ACD) in each IRS round (Table 1). A total of 634 rK39 tests (1 test for each febrile case; average 158.5/round) were performed; of which 26 febrile cases (4.1%; 6.5-cases per round) were recorded positive for VL with more than 14 days fever. The percent of positive rK39 tests was reduced from 4.6% in the first round in 2015 to 3.4% in the second round in 2016 (P = 0.62). The rK39 positive febrile cases searched by different groups recorded were 7 by ASHAs (26.9%; average 1.8 case per round), 6 by squad monitors and SFWs (23.1%; average 1.5 case per round), and 13 (50%; average 3.3 case per round) by RMRIMS teams including the PHC supervisor and trained lab technician. Patients detected as VL positive with rK39 tests were sent to block hospitals for confirmation by doctors and prompt treatment. During the study period (2015–2016), 4.5% of total cases (26 of 574) were diagnosed through ACD. No recurrence was observed among all treated patients during 6 months of post-treatment follow up. In total, 39 suspected PKDL (15 in 2015 and 24 in 2016) cases spotted across the blocks during 2015–2016. The 27 suspected PKDL cases (69.2%; 10 in 2015 and 17 in 2016) that tested initially positive with rK39 tests were sent to RMRIMS, Patna, or the Sadar Hospital, Hazipur, for further confirmation through the skin biopsy test and prompt treatment. The 27 of 27 (100%) patients were confirmed for PKDL with positive skin biopsy tests. This number represents 79.4% of the total PKDL cases (27 of 34 total cases) reported during 2015–2016.

**Table 1:** Details of villages, households, and population covered, and number of febrile cases found positive with rK39 tests during active case detection in the Vaishali District, Bihar, in 2015–2016.

Year (s)	IRS Round (S)	Total Villages Surveyed (N)	Total HHs Covered	Total Population Screened for VL	Total Suspected Febrile Cases (%)	rK39 Positive Febrile Cases (%)	VL (+) Cases Searched by ASHAs (%)	VL (+) Cases Searched by Squad-Monitors and SFWs (%)	VL (+) Cases Searched by RMRIMS’s PHC Supervisor and Lab-Technicians (%)
**2015**	First Round	304	40,292	199,856	218 (0.1%)	10 (4.6%)	3 (30%)	3 (30%)	4 (40%)
	Second Round	312	34,268	175,652	164 (0.1%)	7 (4.3%)	2 (28.6%)	1 (14.3%)	4 (57.1%)
**Total 2015**		616	74,560	375,508	382 (0.1%)	17 (4.5%)	5 (29.4%)	4 (23.5%)	8 (47.1%)
**2016**	First Round	236	23,524	121,203	133 (0.1%)	5 (3.8%)	1 (20%)	1 (20%)	3 (60%)
	Second Round	241	24,878	126,138	119 (0.1%)	4 (3.4%)	1 (25%)	1 (25%)	2 (50%)
**Total 2016**		477	48,402	247,341	252 (0.1%)	9 (3.6%)	2 (22.2%)	2 (22.2%)	5 (55.6%)
**Average**		273.3	30,740.5	155,712.3	158.5 (0.1%)	6.5 (4.1%)	1.8 (26.9%)	1.5 (23.1%)	3.3 (50%)

### Pre-IRS insecticide susceptibility test of local *P*. *argentipes*

The insecticide susceptibility of local *P*. *argentipes* to DDT (4%) and SP (0.05%) was evaluated before the start of each IRS round in the Vaishali District, Bihar, during 2015–2016 (S14 Table). The corrected mortality rate of sand flies against DDT ranged from 40% to 55.6% (overall: 48%; average: 47.7% in the first round of 2015, and 48.3% in the second round in 2015). The results indicate high-level resistance development in local *P*. *argentipes* against DDT. Therefore, later on in the second round of IRS in 2015, after 15 days, DDT (50%; WP) was replaced by SP (alphacypermethrin 5%; WP). Local sand flies were exposed to SP concentrated papers (0.05%) for mortality estimation, and tests were continued during each IRS round for the routine programmatic monitoring of insecticide susceptibility level among local sand flies. The results showed *P*. *argentipes* were highly susceptible to SP during all IRS rounds. The corrected mortality rates recorded were always 100%.

### Pre and post IRS sand fly density estimation

Sand fly densities (pre- and post-IRS) in the sprayed and unsprayed villages were monitored to assess the IRS intervention effect using DDT and SP (S15 Table). *P*. *argentipes* densities fluctuated (i.e. decreases or increases) in all control villages post-IRS in all IRS rounds. A significant difference was observed in *P*. *argentipes* densities collected in SP sprayed and unsprayed villages during the 15 days, 1 month, and 3 months post-IRS collections (t = 4.6; P <0.01). This difference was observed in the 15 days post-IRS collections (t = 3.2; P <0.05) for DDT-IRS periods. No significant difference was observed in the 1-month and 3-month post-IRS collections (t = <1.7; P = 0.12). This result indicates that IRS using DDT did not affect sand fly densities for up to one month post-IRS, and it seemed that sprayed villages remained unprotected from sand fly bites until the next spray. The sand fly reduction rate (RR) post-IRS was much higher in the SP-IRS villages than it was in the DDT-IRS villages in all the IRS rounds (S15 Table). At the 15 days post-IRS (SP round), the sand fly RR ranged between 91.5 and 94.6%. A minimum of 81.5% (maximum 85.9%) of the sand fly RR was maintained in SP-sprayed villages for up to 1 month post-IRS. At 3 months post-IRS, the RR ranged from 33.2% to 43.9%. While in the DDT-IRS round in the intervention villages, the sand fly RR at the 15 days post-IRS ranged from 40.1% to 46.9%, and at the 1 month post-IRS, the RR was recorded between 4.6% and 17.2%. A fast increase in sand fly abundance was observed at the 3 month post-IRS in the DDT round: it ranged from 16.5 to 19.7%.

### VL cases reduction, village endemicity analysis, and target achievement

**Fig 4:**
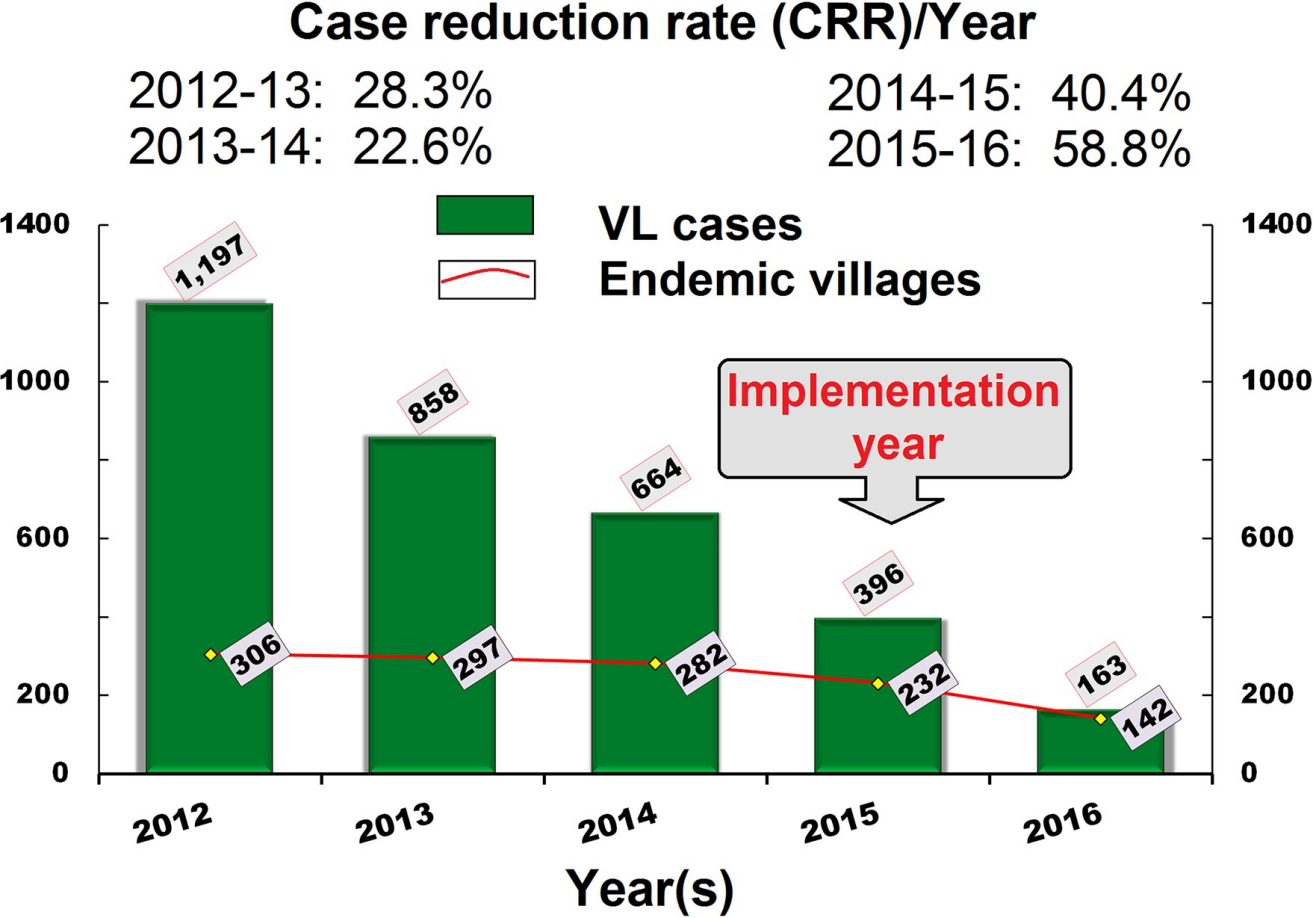
The number of VL cases and affected villages reported each year during 2012–2016 in the Vaishali District, Bihar. The case reduction rate (CRR) was calculated between 2013 and 2016.

**Fig 5:**
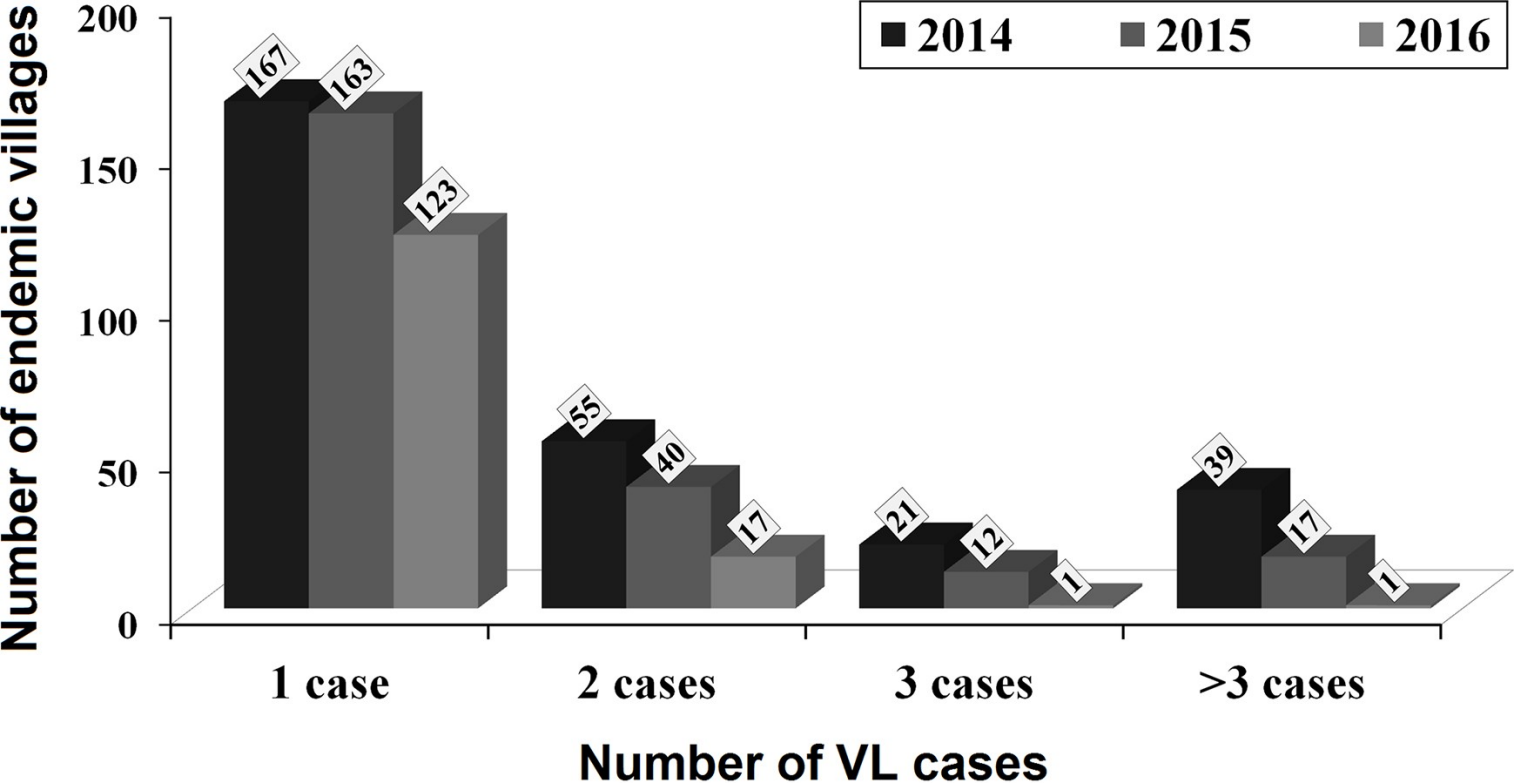
The case wise annual distribution of VL-affected villages during 2014–2016 in the Vaishali District, Bihar.

The number of total new VL-cases and endemic villages gradually decreased from 664 in 2014 to 163 in 2016, and 282 in 2014 to 142 in 2016, respectively (Fig 4). The CRR increased from 22.6% in 2014 to 58.8% in 2016. The average annual CRRs in 2015 and 2016 (i.e. 49.6%/year) were high (almost twofold) compared to the average annual CRRs in 2014 and 2013 (i.e. 25.5%/year). There were 13 to 16 blocks each year in the Vaishali District (16 in 2012, 15 in 2013, and 13 in 2014) reported with ≥1 CIR between 2012 and 2014 (Table 2). The block names reported with <1 CIR varied between 2013 and 2014. In 2015, 6 more blocks (Desri, Goraul, Lalganj, Mahua, Shadei Buzurg, and Patedi Belsar) achieved the elimination target (i.e. <1 case per 10,000 people) along with 3 blocks in 2014 (i.e. Bhagwanpur, Cherahkala, and Rajapakar). All the 16 blocks of the Vaishali District achieved the elimination target in 2016. The annual CIR among the blocks ranged between 0.2 and 0.8 (average 0.4). Fig 5 shows the case wise annual distribution of VL-endemic villages. The total villages reported with one VL case decreased from 167 in 2014 to 123 in 2016 (26.3% reduction; average 13.2%/year). Similarly, the hot-spot villages reported with 2, 3, and >3 cases were gradually decreased from 55, 21, and 39 in 2014 to 17, 1, and 1 in 2016, respectively. The average reduction of 2, 3, and >3 cases villages recorded were 34.5%, 47.6%, and 48.7% annually. On average, 74 endemic villages were VL free annually during 2015–2016. Overall, 8 villages [7 in 2015 (3% of total endemic villages) and 1 in 2016 (0.7% of total endemic villages)] for an average of 4 villages/year (1.9%) were newly endemic during 2015–2016.

**Table 2:** Block wise details of VL cases and its incidence rate (per 10,000 people) in the Vaishali District, Bihar, during 2012–2016.

Name of PHCs	VL Cases					Case Incidence Rate (CIR; 10,000 People)				
	2012	2013	2014	2015	2016	2012	2013	2014	2015	2016
**Bhagwanpur**	40	33	20	17	4	1.9	1.5	0.9	0.7	0.2
**Bidupur**	85	48	41	33	8	3.1	1.7	1.4	1.1	0.3
**Cherahkala**	31	30	11	3	4	2.3	2.2	0.8	0.2	0.3
**Desri**	54	22	16	8	1	5.3	2.1	1.5	0.7	0.1
**Goraul**	25	30	34	14	6	1.4	1.6	1.8	0.7	0.3
**Hajipur**	78	73	71	53	31	1.7	1.5	1.5	1.1	0.6
**Jandaha**	68	55	49	28	7	2.5	2.0	1.7	1.0	0.2
**Lalganj**	48	53	31	20	12	1.8	1.9	1.1	0.7	0.4
**Mahnar**	71	48	51	33	16	3.7	2.5	2.6	1.6	0.8
**Mahua**	129	103	53	27	15	4.5	3.5	1.8	0.9	0.5
**Patedi Belsar**	30	9	11	5	2	3.1	0.9	1.1	0.5	0.2
**Patepur**	107	93	79	43	19	2.9	2.4	2.0	1.1	0.5
**Raghopur**	279	161	125	65	15	11.3	6.4	4.8	2.4	0.6
**Rajapakar**	36	30	15	11	4	2.3	1.8	0.9	0.6	0.2
**Shadei Buzurg**	27	22	26	13	9	2.1	1.6	1.9	0.9	0.6
**Vaishali**	89	48	31	23	10	4.6	2.4	1.5	1.1	0.5
**Total Cases per Year/ Blocks with ≥1 CIR**	1,197	858	664	396	163	16	15	13	7	0

## Discussion

In the current study, we demonstrate for the first time that the achievement of the VL elimination target is feasible at the block level in the highly-affected region of Bihar. Our results show that the annual reduction of new VL cases and endemic villages of at least 40% at the district level is possible when an integrated VL control strategy is implemented under strong supervision and a monitoring system. Among the 33 VL-endemic districts of Bihar, Vaishali, one of the most affected zones, ranked second in endemicity during 2012–2014 [15]. It has 16 blocks, and all blocks had a VL incidence above or near the elimination target (i.e. <1 case per ten thousand people). Our data analysis revealed an average of 29.5% CRR per year during the period 2013–2014 in the Vaishali District. The CRR was observed to gradually increase by 40.4% in 2015 and 58.8% in 2016 after implementation of an integrated VL control strategy from the first round of IRS in 2015. The number of hot-spot villages reported with ≥2 cases were found to decrease considerably during 2016 compared to 2014. This result indicates that the integrated VL control strategy was highly effective to limit the occurrence of new cases at the meso-scale/village level across all blocks in the Vaishali District. This study also suggests that rather than performing a single activity at a different time and/or in different ways, a combined effort including the use of GIS-based epidemiological mapping for IRS village planning; strong supervision; monitoring of program activities; rigorous training of medical officers for effective treatment; the training of ASHAs, PHC supervisors, and squad monitors in the active searching of VL and PKDL cases and IRS management; the training of SFWs and FWs for quality IRS; the use of HCP for quality IRS, logistic monitoring for improved IRS coverage, immediate focal spraying at new case locations, and routine entomological monitoring is very important for control of a high-endemic burden of VL in any location. The most novel approach adopted in the study was the inclusion of non-endemic risk-prone peripheral villages of the hot-spot endemic villages in the delivery of a targeted IRS. This strategy helped to prevent spatio-temporal dynamics of VL case occurrence between endemic and non-endemic villages [17]. As a result, the number of total VL-endemic villages including old and newly-endemic villages gradually decreased during 2015–2016. A previous study performed in the Vaishali District reported 68 new endemic villages annually [17].

Focal spraying is shown to be effective for interrupting intra-village transmission. The approach assumed that the instantaneous covering of all neighboring HHs through FS within 500 m of a new case location could kill all infected sand flies. Thus, it limits ongoing vector transmission among HHs at a broad scale across villages. This fact is well supported by the result of our FS analysis data. Only a few villages of the total FS villages (i.e. average 28.1%/year) appeared with a new case at the end of the year while the percentage was very high for FS uncovered villages (i.e. average 75%/year).

The availability of a treatment facility at the nearest center with doctors in good treatment practice and precise knowledge is an important factor of control of any kind of disease in an affected region. In Vaishali, among six VL treatment centers, all medical personal were trained for the prompt treatment of VL patients with single dose liposomal amphotericin B [32]. This approach has diminished the treatment dependency of local patients at a single center (i.e. the Sadar Hospital at Hazipur). Thus, it has helped reduce delay in the VL treatment process and it has helped break intra- and inter-HH transmission in a village.

Furthermore, an ACD alongside with the prompt treatment of clinically confirmed VL patients played an important role in reducing parasite stability and its available sources among social communities. In this process, we have followed the WHO-T3 model of test, treat, and track (i.e. test every suspected case via a diagnostic test, treat every confirmed case, and track the disease) to lead to better achievement in the Vaishali VL control program. The involvement of different groups in ACD has multiplied the number of eyes in the case monitoring and surveillance system. This has helped to eradicate different types of human reservoirs (VL and PKDL) of *L*. *donovani* among unnoticed pockets across the rural endemic villages. This may also be a legitimate method to disrupt VL transmission between HHs/villages. Overall, 4.5% of the total VL cases and a vast majority of the PKDL cases (79.4%) were reported to the case surveillance system through ACD during 2015–2016. The positive rK39 test rate was 4.1% per round during the study period. Our result was in accordance with the previous studies performed in the endemic region of Bihar [33, 34]. The early detection of VL and PKDL cases and treating the patients earlier to interrupt indigenous transmission is an important factor for successful control of the VL case burden in a highly endemic region.

On the other hand, the success and failure of a disease control program is directly or indirectly related to the quality of the staff involved in the work. Therefore, human resource development is necessary for all disease control programs. It brings about the transmission and acquisition of skills, abilities, and competences that enable individuals to work, and contributes to the improvement of the program at large. The results of this study show that a great improvement in all aspects of the VL control program occurred after the training of all staff including medical doctors and IRS workers. The regular training of spray applicators, squad monitors, ASHAs, KTSs, and PHC supervisors resulted in increasing the spray coverage including the number of fully sprayed HHs over the IRS rounds. Incidents of refused and partially sprayed HHs decreased considerably from the strategic control implementation period in the first round of IRS in 2015 to the second round of IRS in 2016. The deployment of monitors at the squad level were found most effective in IRS-refusal conversion, as more than 50% of the total refused HHs were converted to sprayed status by a squad monitor during the Vaishali study period. However, the PHC supervisors and KTSs played the lead role among all staff such as FWs, SFWs, squad monitors, and ASHAs. Hard and tough refusals from HHs were converted by the PHC supervisors and KTSs.

While IRS remains the backbone of VL vector control, improved HH coverage across all targeted villages is necessary. During the study period, 100% coverage of targeted villages was ensured in each IRS round with 96.7% HH coverage. In addition to human resource development, quality assurance is equally important to meet the needs of the program to achieve the target [22]. Thus, the focus of the quality assurance process is the monitoring and assessment of program activities to improve the activities below minimum standards. In the Vaishali study, a multi-level monitoring and supervision system was developed and implemented. A considerable impact has been shown from all levels of IRS monitoring and supervision groups. The results of the HH level post-IRS survey revealed a great improvement in spray applicator activities as well as IRS quality. The mismatch of sprayed and unsprayed HHs between the IRS-register and field-reality was minimized successfully in a negligible amount. Moreover, remarkable progress was made in the percentage of uniformly sprayed HHs up to the 1.8m height. Incidents like missed HHs areas and washed out and repainted HH numbers were also eliminated. Along with the IRS coverage, the availability of insecticide on sprayed walls should be ensured to obtain the long-lasting mass impact of the IRS-based vector control program [35].

Although monitoring played an important role in IRS quality improvement, the consistency and maintenance of IRS quality among all HHs throughout the program is another challenging responsibility. A permissible system is required with extra equipment available to achieve an instant corrective measure. During the Vaishali program, we developed an IRS warehouse in collaboration with the State Government of Bihar for the quick dispatch and easy delivery of all equipment directly to the squads for the replacement of faulty and missing equipment during IRS. We also performed quality assessment between pumps for achieving improved IRS quality among HHs across the IRS villages. The results showed that HCPs are better than CSPs in all aspects like IRS quality and operational feasibility. The IQK results also support the better IRS quality of HCPs (the average DDT found was 1.18 g ai/m^2^). In more than 58% of the sample HHs, the DDT quantity on the walls was found in the target range and no HH was undersprayed. Thus, the IRS quality of the program was improved by implementing HCPs in all 16 blocks of the Vaishali District. This suggests that a good IRS program should have a suitable monitoring and evaluation system in place that allows evidence-based decision making. Our activities are in line with the guidelines of WHO and Tropical Disease Research (TDR) [20].

The results of an awareness survey showed considerable change in the knowledge, attitudes, and practices related to VL (including its symptoms and facility of treatment at the government hospitals), sand flies, and its control activities among different social communities before and after the sensitization and mobilization program. A considerable number of people (i.e. >51%) started to use precautions to avoid sand fly bites and practice daily cleanliness at their HH premises. These measures maximized the vector control impact in sprayed HHs. The dwellers of unsprayed HHs were associated with single protection at the least. Moreover, the microphone announcements and ASHAs were found to be the highest effective media to inform the villagers about the IRS program. This strategy helped reduce the number of locked HHs at the minimum level among social communities. The results of the post-IRS survey showed that more than 64.3% of HHs had received pre-IRS information through the microphone announcements and ASHAs both. Our results support the results of previous studies performed in rural villages in Bihar [36].

The routine monitoring of entomological data on transmission risk and targeting the right vector with the appropriate intervention are the two most important factors for controlling any kind of vector borne disease [37, 38]. Indoor residual spraying using DDT has historical track records of VL control through minimizing sand fly abundance in the endemic regions of Bihar [39]. Our entomological data analysis results showed that DDT is not very effective for sand fly control in Vaishali. A quick resurgence of sand flies with high density was observed at one month post-IRS. The results of a susceptibility test confirmed the resistance development of local *P*. *argentipes* to DDT. Insecticide susceptibility monitoring studies previously performed in other endemic regions of Bihar have also found resistance development to DDT in local sand flies [12, 13, 26]. Therefore, the immediate implementation of SP occurred, as the mortality of local *P*. *argentipes* to SP has always been recorded at the 100% level during WHO-based susceptibility evaluation tests. A significant difference in sand fly densities was observed between SP sprayed and unsprayed villages. The reduction rate was always recorded at the >81% up to 1 month post-IRS and 33% at least at 3 months post-IRS. This result suggests that the entomological monitoring including sand fly abundance pre- and post-IRS, and insecticide susceptibility should be mandatory in the VL vector control program in all the endemic villages in the District of Bihar as well as in India. A strict monitoring system must be implemented for reviewing the progress of entomological observation routinely [12, 37].

The Vaishali study is an integrated VL control strategy. The estimated total cost for executing all activities at one time could be one difficulty in replicating the model in other endemic districts of Bihar. Minor modifications might also be required in the Vaishali control strategy for implementing it in a new endemic region. Another limitation of the Vaishali study is that it has not been used on a small scale but only during the attacking phase when the cases are widely distributed. Thus, reactive or proactive IRS might be cost effective during the sustenance phase when the cases are more localized and focalized, which has not been tested in the Vaishali study. Despite the limitations, the Vaishali study has been implemented successfully with high acceptability among different social communities. The Vaishali strategy is highly effective in achieving the elimination target in all endemic blocks.

In conclusion, the results of this study show the considerable impact of an integrated VL control strategy to achieve the elimination target in all blocks of the Vaishali District. This strategy could be the stepping stone for achieving the VL elimination target at the block level in other highly affected districts of Bihar. Application of the Vaishali strategy in all highly endemic blocks of Bihar could ultimately help reduce new VL cases and its spread in new geographical areas on the mass scale within the state of Bihar. This step could be a game changer for achieving the VL elimination target in Bihar as well as in the country by 2020. Along with the existing VL vector control method (i.e. IRS using DDT in endemic villages in the last three years) and case management activities (i.e. case detection, complete treatment, and post-treatment follow up), the addition of new methods like the spraying of peripheral villages of hotspot villages; the focal spraying of all HHs around 500 m of a new VL case location; the use of SP IRS for vector control; the use of HCPs instead of CSPs for IRS, designing and implementing a multi-level monitoring system; the involvement of ASHAs, IRS-members, monitors and supervisors in ACD; community awareness development; and routine entomological and epidemiological monitoring have increased the efficiency of the VL control program at a considerable level. The spraying of non-endemic high-risk peripheral villages along with endemic villages resulted in substantial reduction in village endemicity. The involvement of ASHAs, SFWs, FWs, and squad monitors in febrile case searching maximized the quantity of ACD-based VL cases reported to the program surveillance system. The rigorous training and monitoring of spray applicators and squad monitors improved the quality of IRS; which resulted in the reduction of sand fly densities across villages in the Vaishali District. Moreover, the implementation of HCPs in the IRS program and the insecticide switchover to SP from DDT accelerated the effectiveness of the control strategy. The implementation of a multi-level monitoring system from the district to HH levels has helped perform an effective and improved IRS. Especially the deployment of monitors at the squad level and supervisors at the PHC level improved the IRS quality and its coverage at the household level. Moreover, awareness creation through small and large media activities, microphone announcements, and ASHAs played an important role in IRS acceptance and active and passive VL case surveillance among social communities. Thus, a fast reduction was observed in new VL occurrence and distribution of infected villages in the Vaishali District. Our study suggests that VL elimination is possible from the endemic regions of Bihar if the Vaishali strategy is implemented properly under strong monitoring and supervision. For policy makers, government organizations, and non-governmental organizations in the State of Bihar, we strongly recommend implementing the Vaishali strategy in all endemic districts to achieve the VL elimination target by 2020.

## Supporting information

S1 Checklist STROBE checklist. (DOC)

S1 Fig GIS based mapping of new VL cases at the household level in Vaishali district, Bihar (India) for IRS-based VL vector control immediately followed by new case report. A GIS-database built in the remote sensing project of ICMR-Rajendra Memorial Research Institute of Medical Sciences was used to create the maps in the figure.
(TIF)

S2 Fig Hierarchy of staffs involved in monitoring and supervision of IRS activities in Vaishali district, Bihar (India) during 2015–16. (TIF)

S1 Appendix An example of GIS based epidemiological mapping used for IRS-village selection at the block level (Panel ‘A-P’) of Vaishali district, Bihar (India). The red line around the new villages administrative boundary shows the nearest neighboring VL-endemic hot-spot and high-risk non-endemic villages within 500 m. A GIS-database built in the remote sensing project of ICMR-Rajendra Memorial Research Institute of Medical Sciences was used to create the maps in the figure.
(DOCX)

S2 Appendix Details of different types IEC/BCC (Panel ‘A-M’) conducted during VL-vector control programme in Vaishali district, Bihar (India) during 2015–16. (DOCX)

S1 Table IRS schedule of Kala-azar vector control programme during 2015 and 2016 in Vaishali district Bihar. (DOCX)

S2 Table Details of IEC/BCC materials distributed during 2015 and 2016 in Vaishali district, Bihar. (DOCX)

S3 Table Definition of refused, partially- and fully-sprayed, and missed houses, false stenciling, and doubling in house serial number during IRS-based VL-vector control programme in Vaishali district, Bihar, India. (DOCX)

S4 Table The total number of villages targeted and covered during the first and second rounds of IRS in 2015 and 2016 in the Vaishali District, Bihar. (DOCX)

S5 Table Details of population and HH data targeted, covered, and sprayed during the first and second rounds of IRS in 2015 and 2016 in the Vaishali District, Bihar. (DOCX)

S6 Table. Details of villages, population, and HHs data targeted and covered by FS during IRS in 2015–2016 in the Vaishali District, Bihar (DOCX)

S7 Table The number of households that refused IRS and the conversion rate to sprayed status by squad monitors, ASHAs, KTSs, and PHC supervisors during IRS in the Vaishali District, Bihar, in 2015–2016. (DOCX)

S8 Table Household level post-IRS survey report regarding the IRS activities and quality spraying in the villages of the Vaishali District, Bihar, during 2015–2016. (DOCX)

S9 Table Details of the damaged, faulty, and missing equipment repaired or newly provided during IRS in the Vaishali District, Bihar, in 2015–2016. (DOCX)

S10 Table The impact of IEC/BCC and social mobilization on the VL control program assessed pre- and post-IRS in the Vaishali District, Bihar. (DOCX)

S11 Table Survey report for HHs that received pre-IRS information from different sources, and the awareness developed about post-IRS activities in the Vaishali District, Bihar. (DOCX)

S12 Table Standardization of hand compression pump (HCP) for IRS in the Vaishali District, Bihar. (DOCX)

S13 Table Household based IRS survey for assessing the spray quality between HCPs and CSPs. (DOCX)

S14 Table Insecticide susceptibility status of *P*. *argentipes* to DDT (4%) and SP (5%) assessed using the WHO-based tube method in the Vaishali District, Bihar, during 2015–2016. (DOCX)

S15 Table The results of *P*. *argentipes* densities caught in sprayed and unsprayed villages in pre- and post-IRS periods in the Vaishali District, Bihar, during 2015–2016. The percent of change in post-IRS sand fly densities was calculated to assess the IRS intervention effect using DDT and SP.
(DOCX)
