# Supplementary material for: Kala-azar elimination in a highly-endemic district of Bihar, India: A success story
Source: PLoS Negl Trop Dis. 2020 May 4;14(5):e0008254. doi: 10.1371/journal.pntd.0008254 (PMC7224556; doi:10.1371/journal.pntd.0008254)
Supplement: S1 Checklist — (DOC) [file pntd.0008254.s001.doc]

S1 Checklist: STROBE checklist

|  | Item No. | Recommendation | Page  No. | Relevant text from manuscript |
| --- | --- | --- | --- | --- |
| **Title and abstract** | 1 | (*a*) Indicate the study’s design with a commonly used term in the title or the abstract | 1 | Please see the title of the manuscript |
|  |  | (*b*) Provide in the abstract an informative and balanced summary of what was done and what was found | 2 & 3 | Paragraph 1, 2, 3 and 4 |
| Introduction | | | |  |
| Background/rationale | 2 | Explain the scientific background and rationale for the investigation being reported | 5, 6, 7 | Paragraph 1, 2 and 3 |
| Objectives | 3 | State specific objectives, including any prespecified hypotheses | 6 & 7 | Last paragraph 4 and 5 |
| Methods | | | |  |
| Study design | 4 | Present key elements of study design early in the paper | 7 - 19 | 1^st^ to 15^th^ paragraphs of the method section |
| Setting | 5 | Describe the setting, locations, and relevant dates, including periods of recruitment, exposure, follow-up, and data collection | 9 - 19 | Method section: 1^st^ to 14^th^ paragraphs |
| Participants | 6 | (*a*) *Cohort study*—Give the eligibility criteria, and the sources and methods of selection of participants. Describe methods of follow-up  *Case-control study*—Give the eligibility criteria, and the sources and methods of case ascertainment and control selection. Give the rationale for the choice of cases and controls  *Cross-sectional study*—Give the eligibility criteria, and the sources and methods of selection of participants | 8, 10,11,16  17, 18, 19 | Method section: paragraphs 2, 3, 12, & 15 |
|  |  | (*b*) *Cohort study*—For matched studies, give matching criteria and number of exposed and unexposed  *Case-control study*—For matched studies, give matching criteria and the number of controls per case | Not applicable |  |
| Variables | 7 | Clearly define all outcomes, exposures, predictors, potential confounders, and effect modifiers. Give diagnostic criteria, if applicable | 9 - 19 | Method section: 3^rd^ to 16^th^ paragraphs |
| Data sources/ measurement | 8* | For each variable of interest, give sources of data and details of methods of assessment (measurement). Describe comparability of assessment methods if there is more than one group | 9 - 19 | Method section: 3^rd^ to 16^th^ paragraphs |
| Bias | 9 | Describe any efforts to address potential sources of bias | 14, 15, 19 | Method section: 10^th^ & 16^th^ paragraphs |
| Study size | 10 | Explain how the study size was arrived at | 3-10 & 12-15 | Method section: paragraphs 9^th^ – 15^th^ and 16^th^ – 19^th^ |

| Quantitative variables | 11 | Explain how quantitative variables were handled in the analyses. If applicable, describe which groupings were chosen and why | 9-19 | In each IRS round, and before, during and after the IRS. |
| --- | --- | --- | --- | --- |
| Statistical methods | 12 | (*a*) Describe all statistical methods, including those used to control for confounding | 8, 15, 16 19 | Method section: paragraphs 2, 11, 12 & 16 |
|  |  | (*b*) Describe any methods used to examine subgroups and interactions | Not applicable |  |
|  |  | (*c*) Explain how missing data were addressed | Not applicable |  |
|  |  | (*d*) *Cohort study*—If applicable, explain how loss to follow-up was addressed  *Case-control study*—If applicable, explain how matching of cases and controls was addressed  *Cross-sectional study*—If applicable, describe analytical methods taking account of sampling strategy | 8, 10,11,16  17, 18, 19 | We calculated both the case incidence rate and case number in VL affected villages. We performed vector control in VL endemic villages of last 3-years and implementation year. We monitored the vector densities in the VL affected villages. |
|  |  | (*e*) Describe any sensitivity analyses | Not applicable |  |
| Results | | | | |
| Participants | 13* | (a) Report numbers of individuals at each stage of study—eg numbers potentially eligible, examined for eligibility, confirmed eligible, included in the study, completing follow-up, and analysed | 35, 36 | Results: paragraphs 11 |
|  |  | (b) Give reasons for non-participation at each stage | 35, 36 | Results: paragraphs 11 |
|  |  | (c) Consider use of a flow diagram | 37 | Results: paragraphs 11  Instead of a flow chart we preferred to present this in a table (Table 12) |
| Descriptive data | 14* | (a) Give characteristics of study participants (eg demographic, clinical, social) and information on exposures and potential confounders | 37 | Table 12 |
|  |  | (b) Indicate number of participants with missing data for each variable of interest |  | Not applicable |
|  |  | (c) *Cohort study*—Summarise follow-up time (eg, average and total amount) | 35, 36 | Results: paragraphs 11 |
| Outcome data | 15* | *Cohort study*—Report numbers of outcome events or summary measures over time | 35, 36 | Results: paragraphs 11 |
|  |  | *Case-control study—*Report numbers in each exposure category, or summary measures of exposure | 40-43 | Results: paragraphs 13&14  Tables 14&15 |
|  |  | *Cross-sectional study—*Report numbers of outcome events or summary measures | 40-43 | Results: paragraphs 13&14  Tables 14&15 |
| Main results | 16 | (*a*) Give unadjusted estimates and, if applicable, confounder-adjusted estimates and their precision (eg, 95% confidence interval). Make clear which confounders were adjusted for and why they were included | Not applicable |  |
|  |  | (*b*) Report category boundaries when continuous variables were categorized | Not applicable |  |
|  |  | (*c*) If relevant, consider translating estimates of relative risk into absolute risk for a meaningful time period | Not applicable |  |
| Other analyses | 17 | Report other analyses done—eg analyses of subgroups and interactions, and sensitivity analyses | Not applicable |  |

| Discussion | | | | |
| --- | --- | --- | --- | --- |
| Key results | 18 | Summarise key results with reference to study objectives | 44-49 | Discussion section: Paragraphs 2-5 |
| Limitations | 19 | Discuss limitations of the study, taking into account sources of potential bias or imprecision. Discuss both direction and magnitude of any potential bias | 49-50 | Discussion section: Paragraphs 6 |
| Interpretation | 20 | Give a cautious overall interpretation of results considering objectives, limitations, multiplicity of analyses, results from similar studies, and other relevant evidence | 50 | Last paragraph |
| Generalisability | 21 | Discuss the generalisability (external validity) of the study results | 44-49 | Discussion section: Paragraphs 2-5 |
| Other information | |  | | |
| Funding | 22 | Give the source of funding and the role of the funders for the present study and, if applicable, for the original study on which the present article is based | 2 | This research was supported by the intramural program of the Rajendra Memorial Research Institute of the Medical Sciences, Indian Council of Medical Sciences (ICMR). |
