# Supplementary figures and images for: Kala-azar elimination in a highly-endemic district of Bihar, India: A success story
Source: PLoS Negl Trop Dis. 2020 May 4;14(5):e0008254. doi: 10.1371/journal.pntd.0008254 (PMC7224556; doi:10.1371/journal.pntd.0008254)

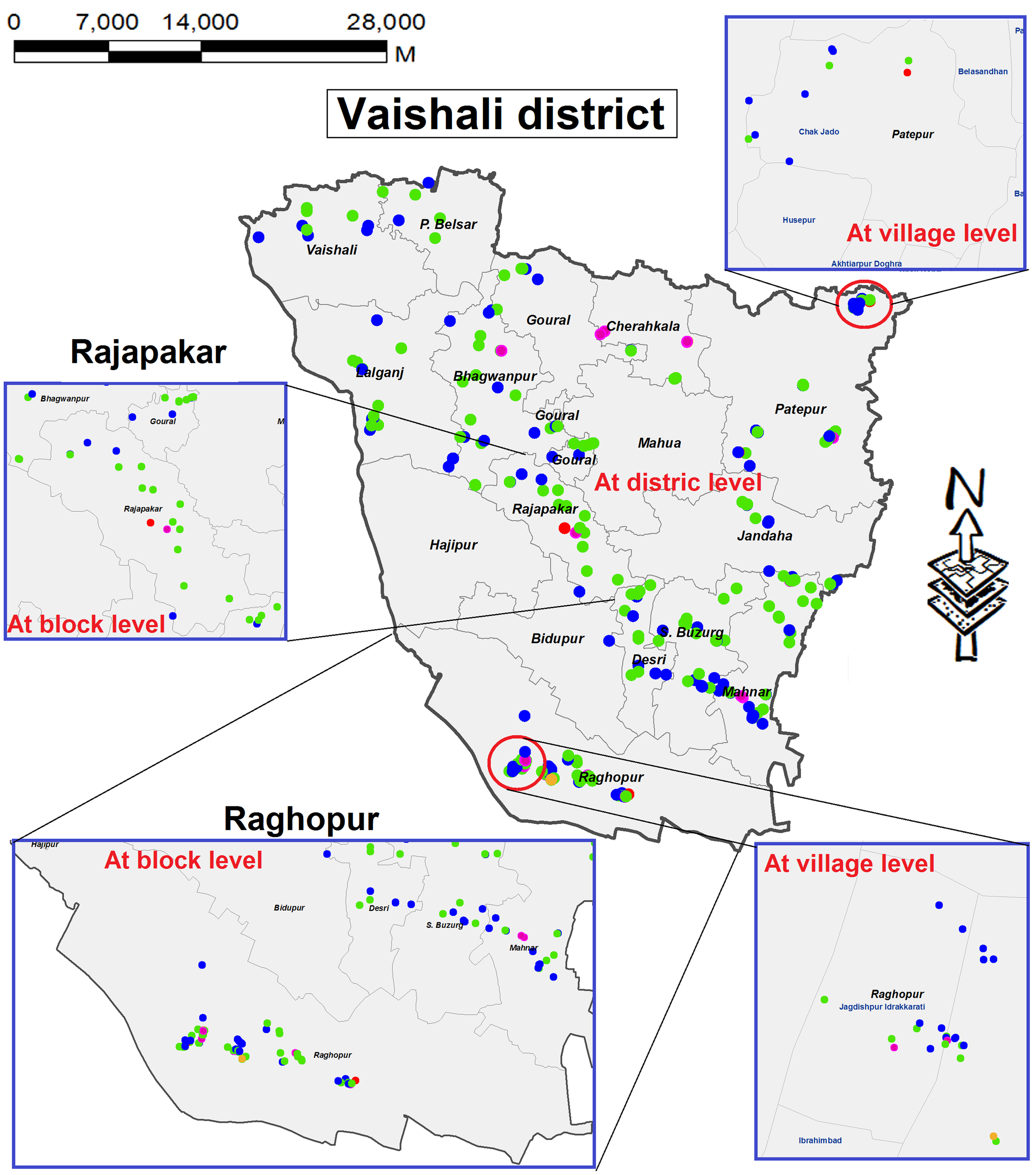

Supplement: S1 Fig — A GIS-database built in the remote sensing project of ICMR-Rajendra Memorial Research Institute of Medical Sciences was used to create the maps in the figure. (TIF) [file pntd.0008254.s002.tif]

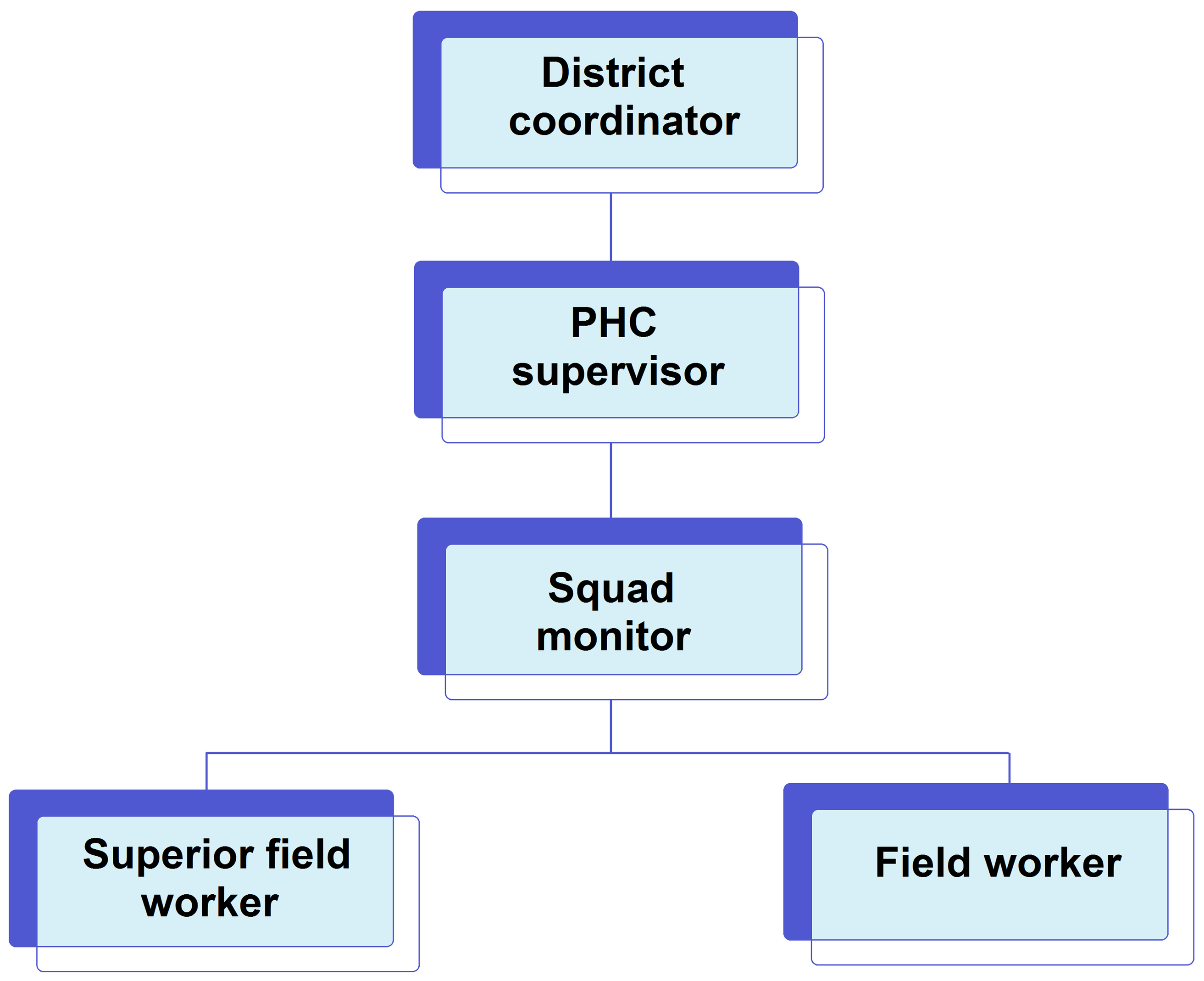

Supplement: S2 Fig — (TIF) [file pntd.0008254.s003.tif]
