# Supplementary material for: Kala-azar elimination in a highly-endemic district of Bihar, India: A success story
Source: PLoS Negl Trop Dis. 2020 May 4;14(5):e0008254. doi: 10.1371/journal.pntd.0008254 (PMC7224556; doi:10.1371/journal.pntd.0008254)

**S1 Appendix. An example of GIS based epidemiological mapping used for IRS-village selection at the block level (Panel ‘A-P’) of Vaishali district, Bihar (India). The red line around the new villages administrative boundary shows the nearest neighboring VL-endemic hot-spot and high-risk non-endemic villages within 500 m. A GIS-database built in the remote sensing project of ICMR-Rajendra Memorial Research Institute of Medical Sciences was used to create the maps in the figure.**

1. **Bhagwanpur**


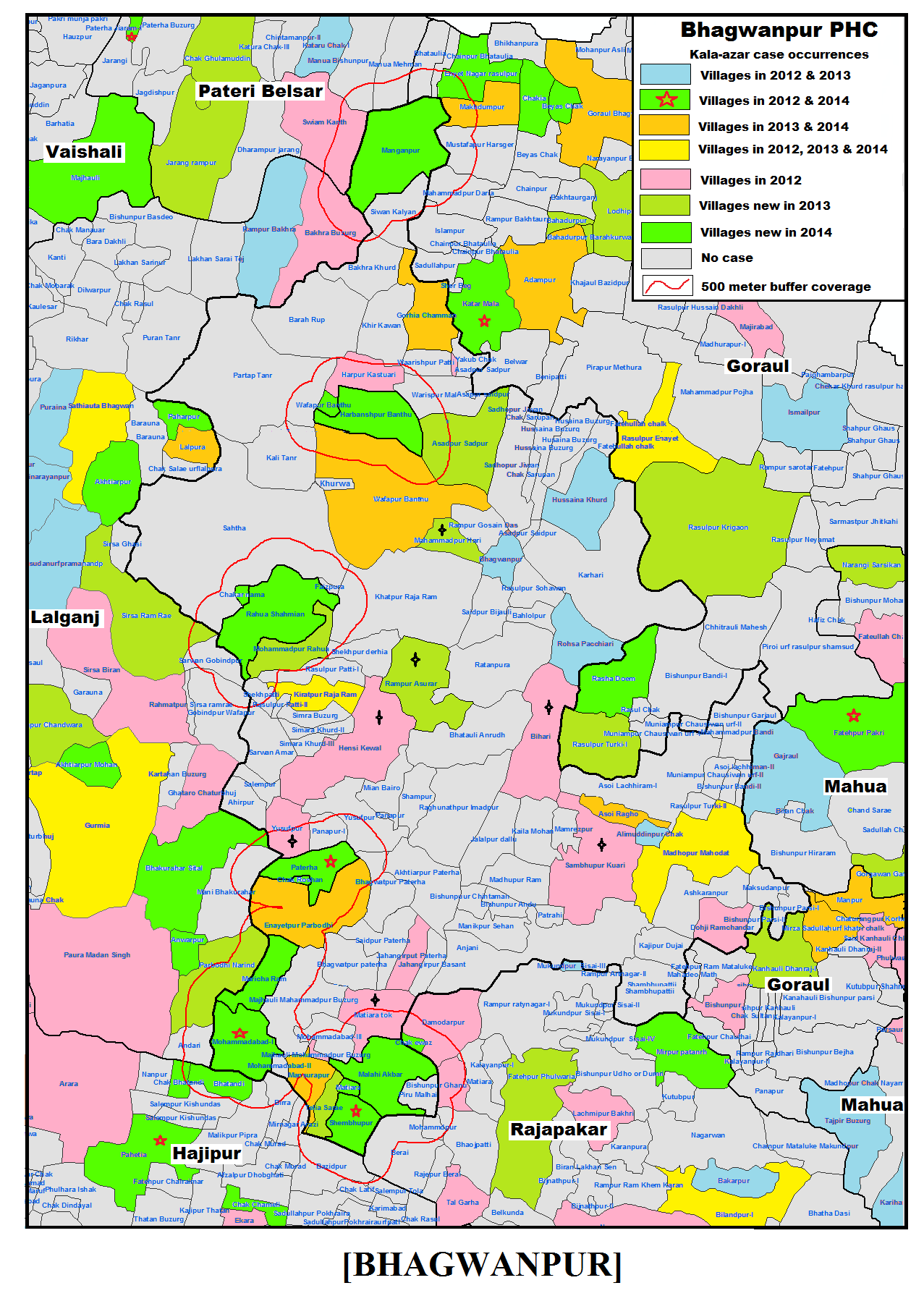


1. **Bidupur**


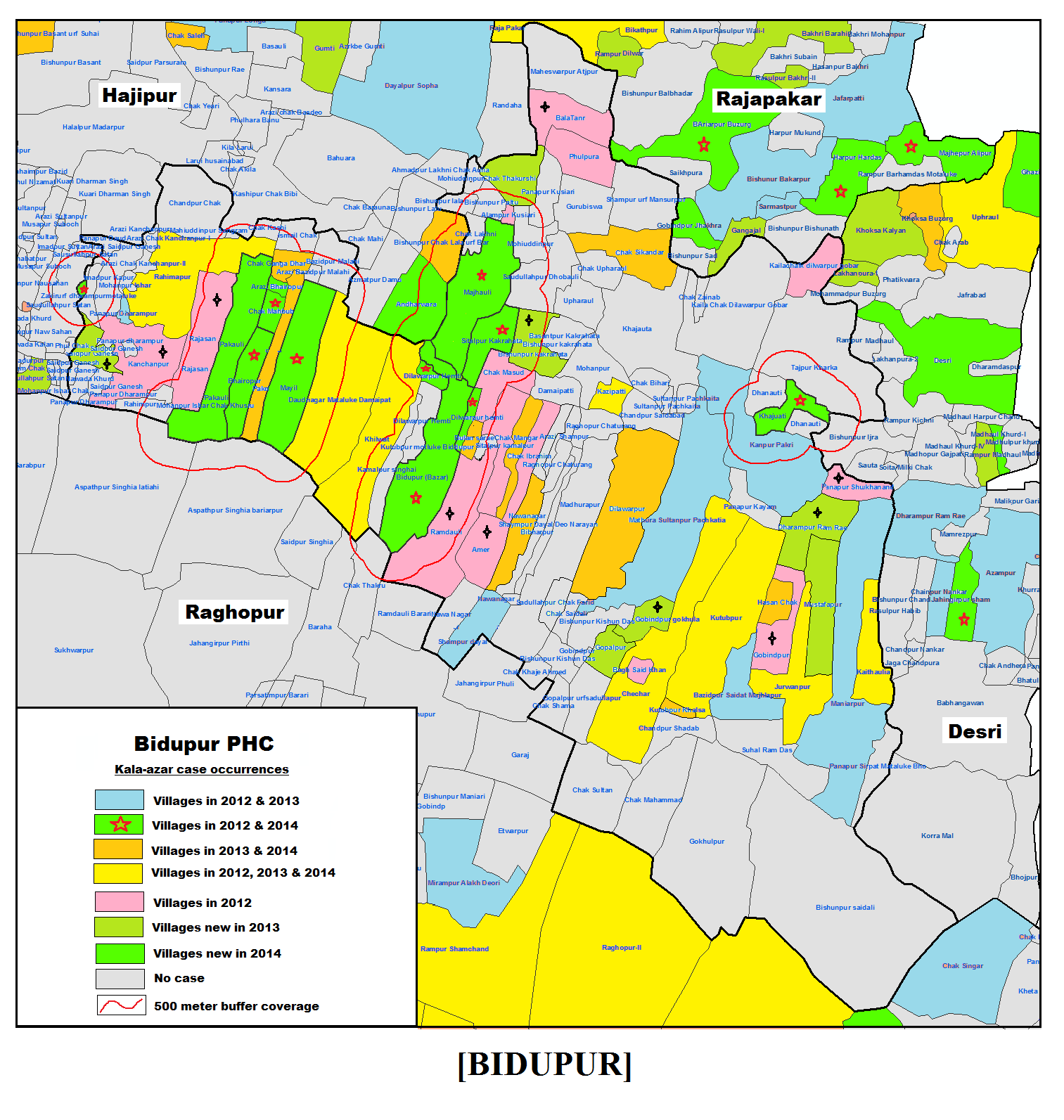


1. **Chehra kalan**


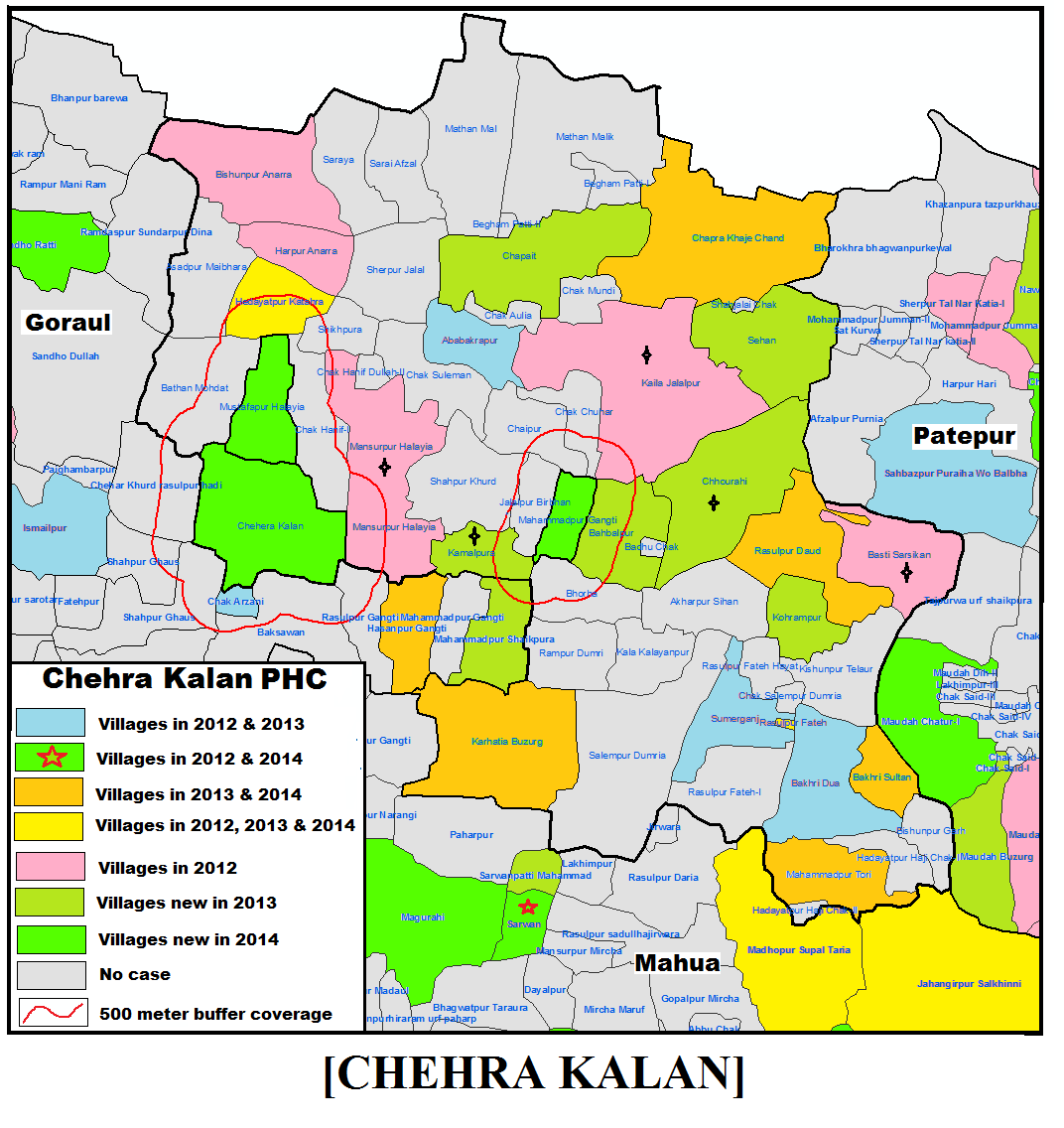


1. **Desri**


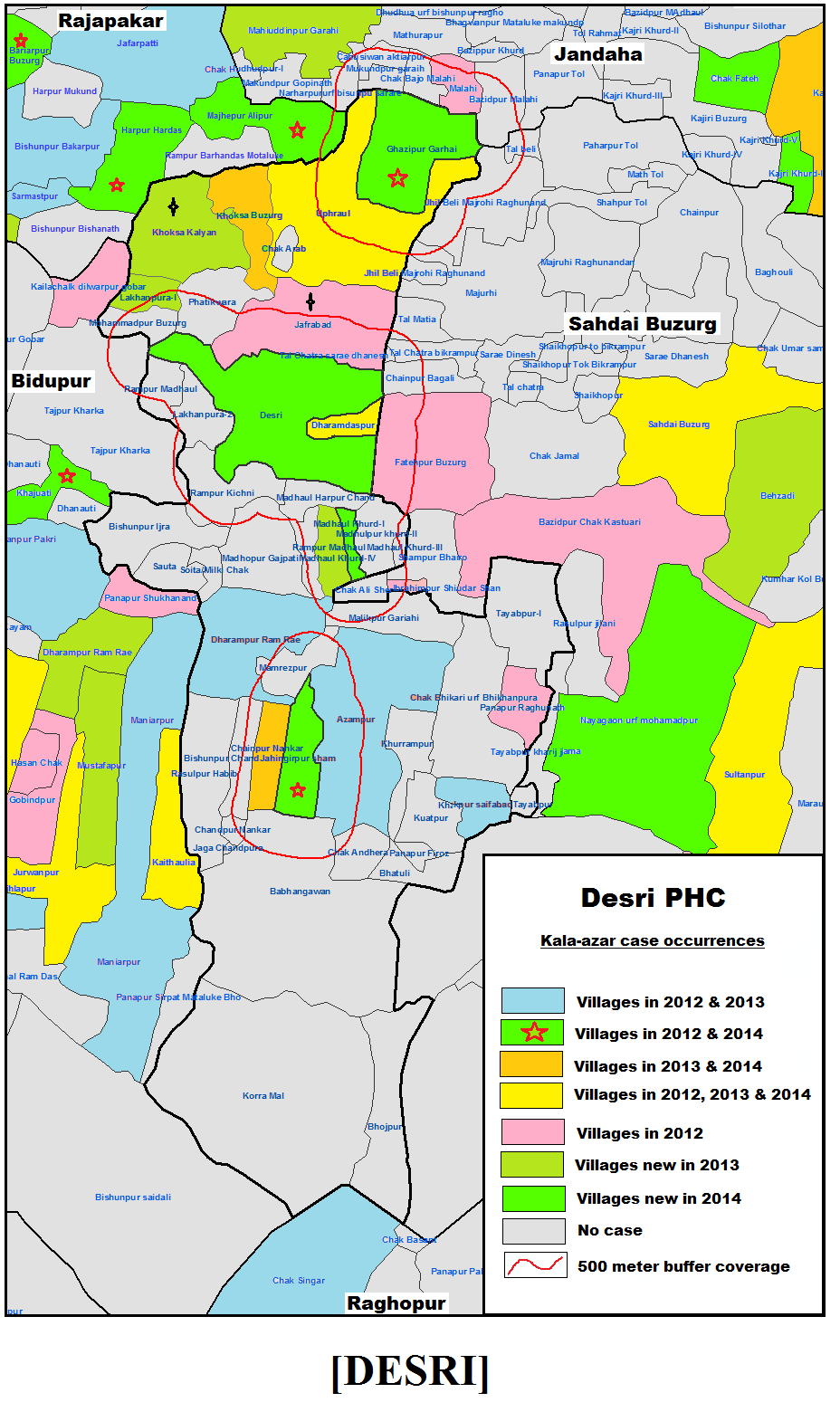


1. **Goraul**


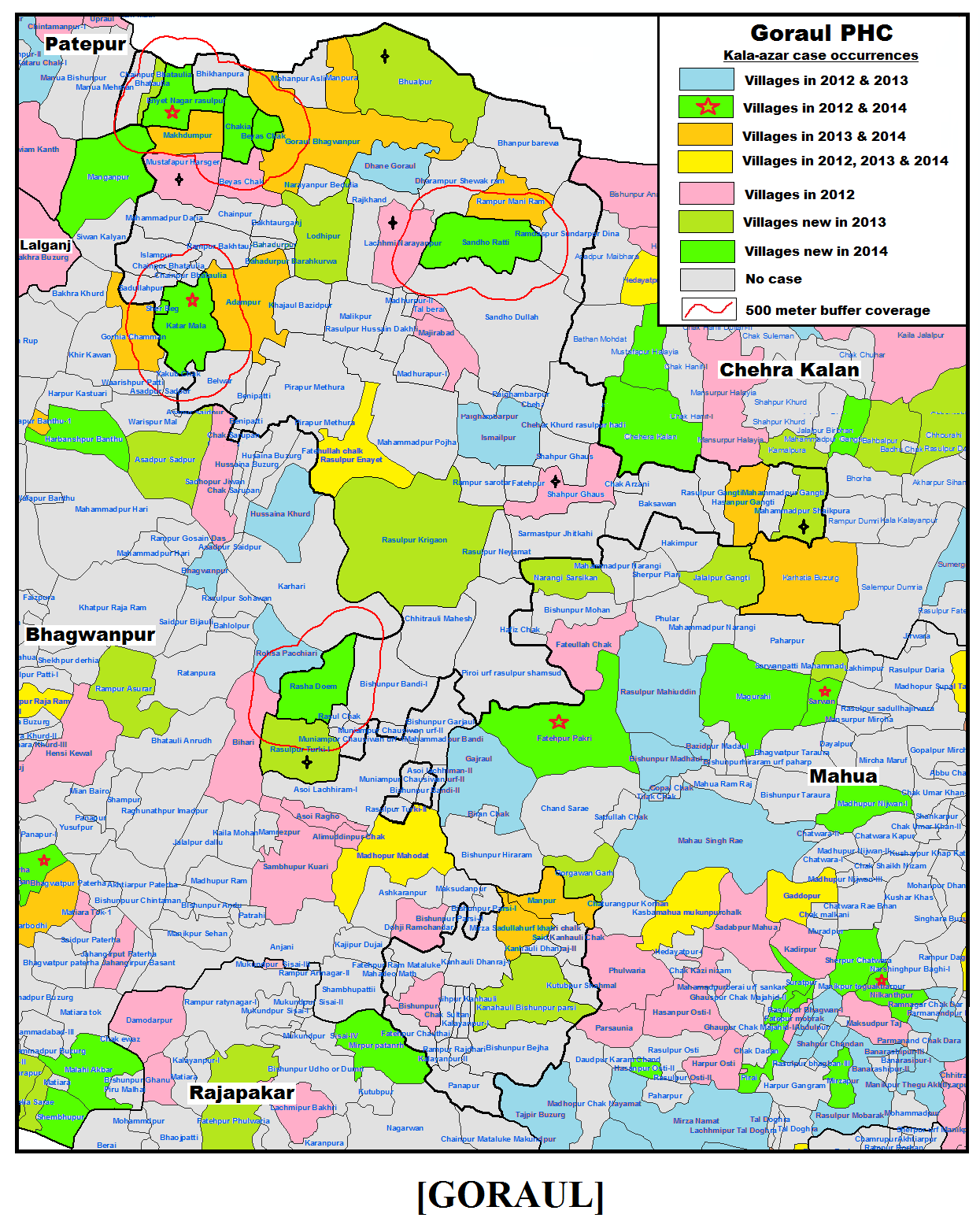


1. **Hajipur**


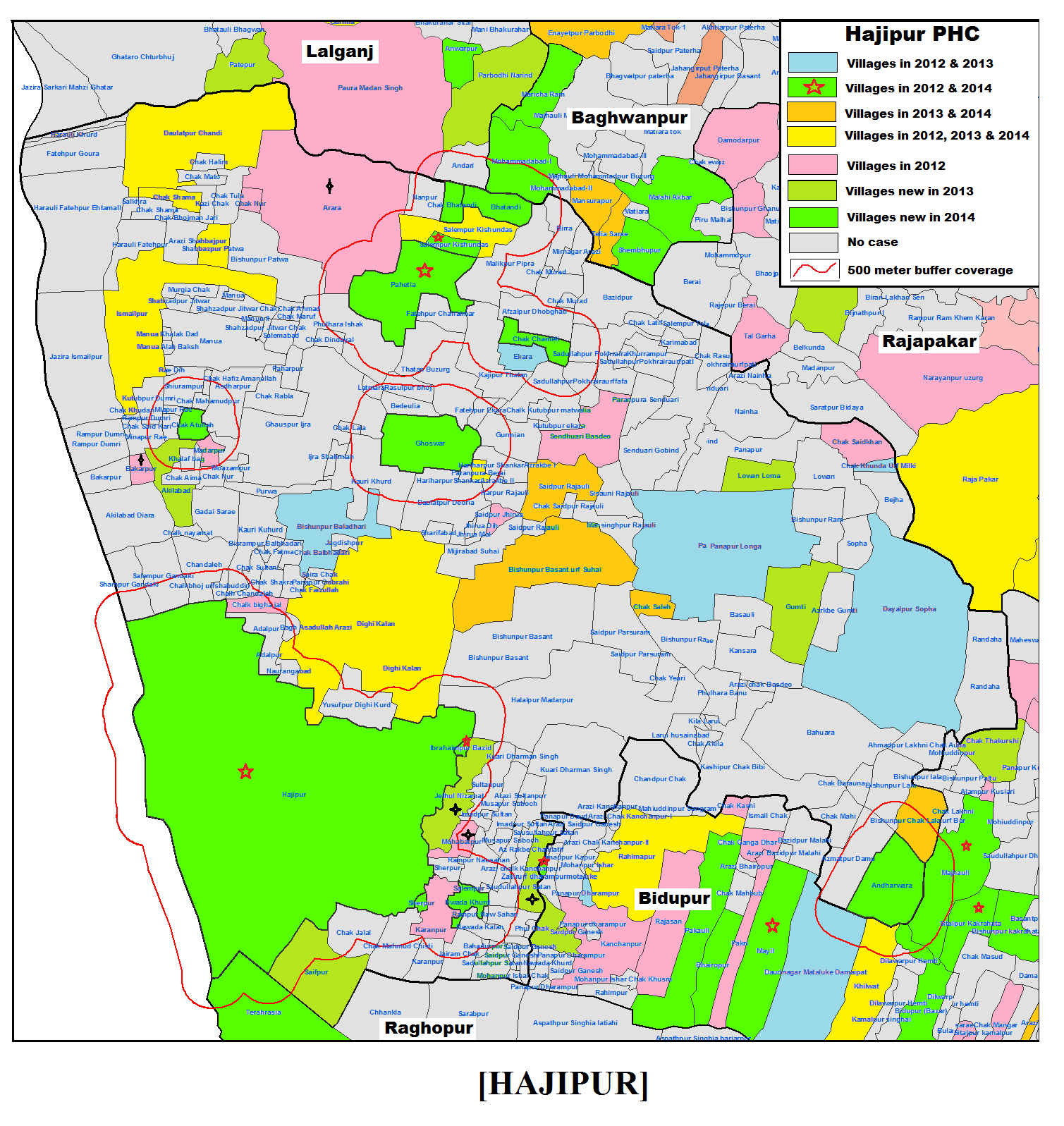


**J. Jandaha**


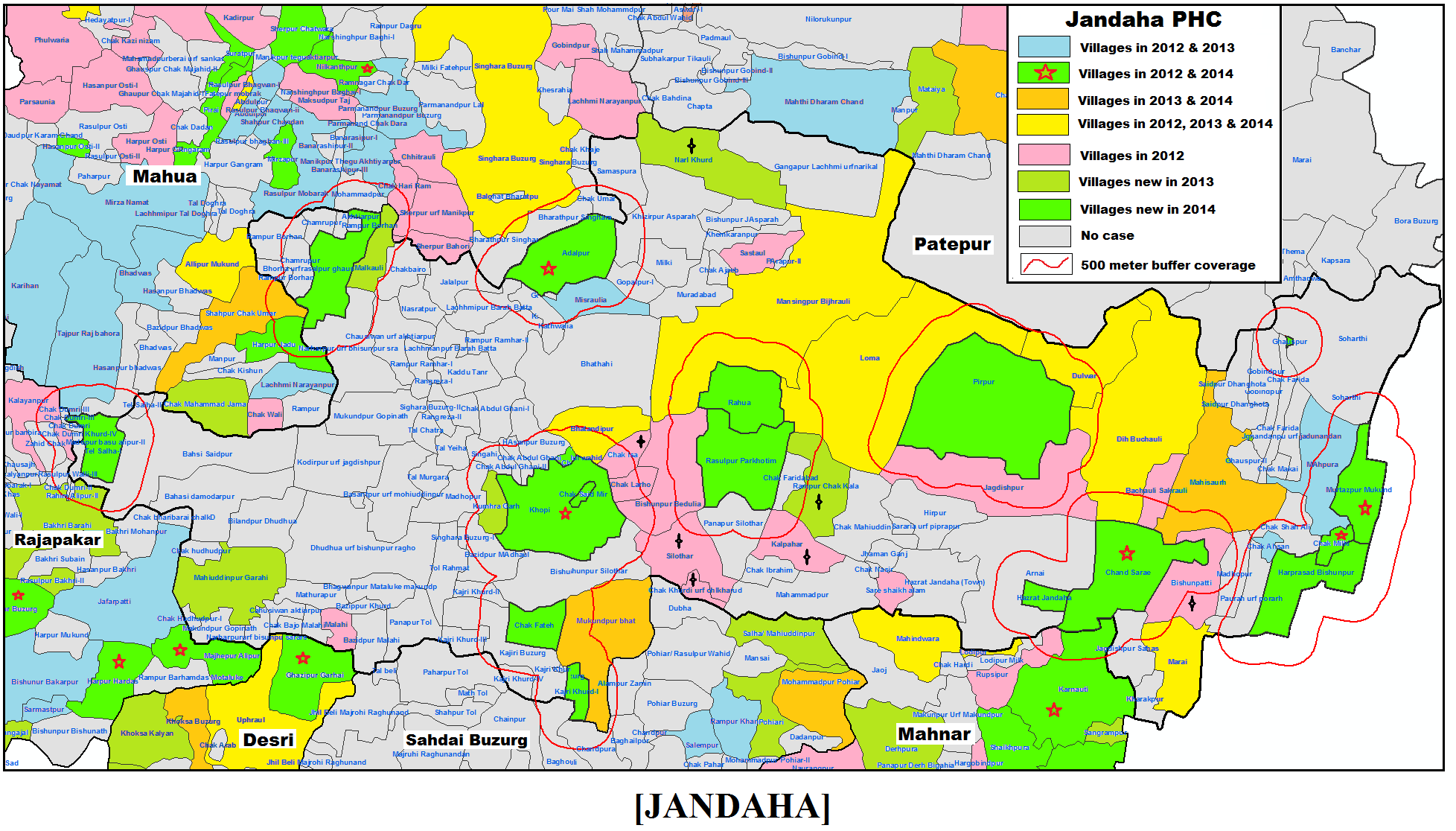


**K. Lalganj**


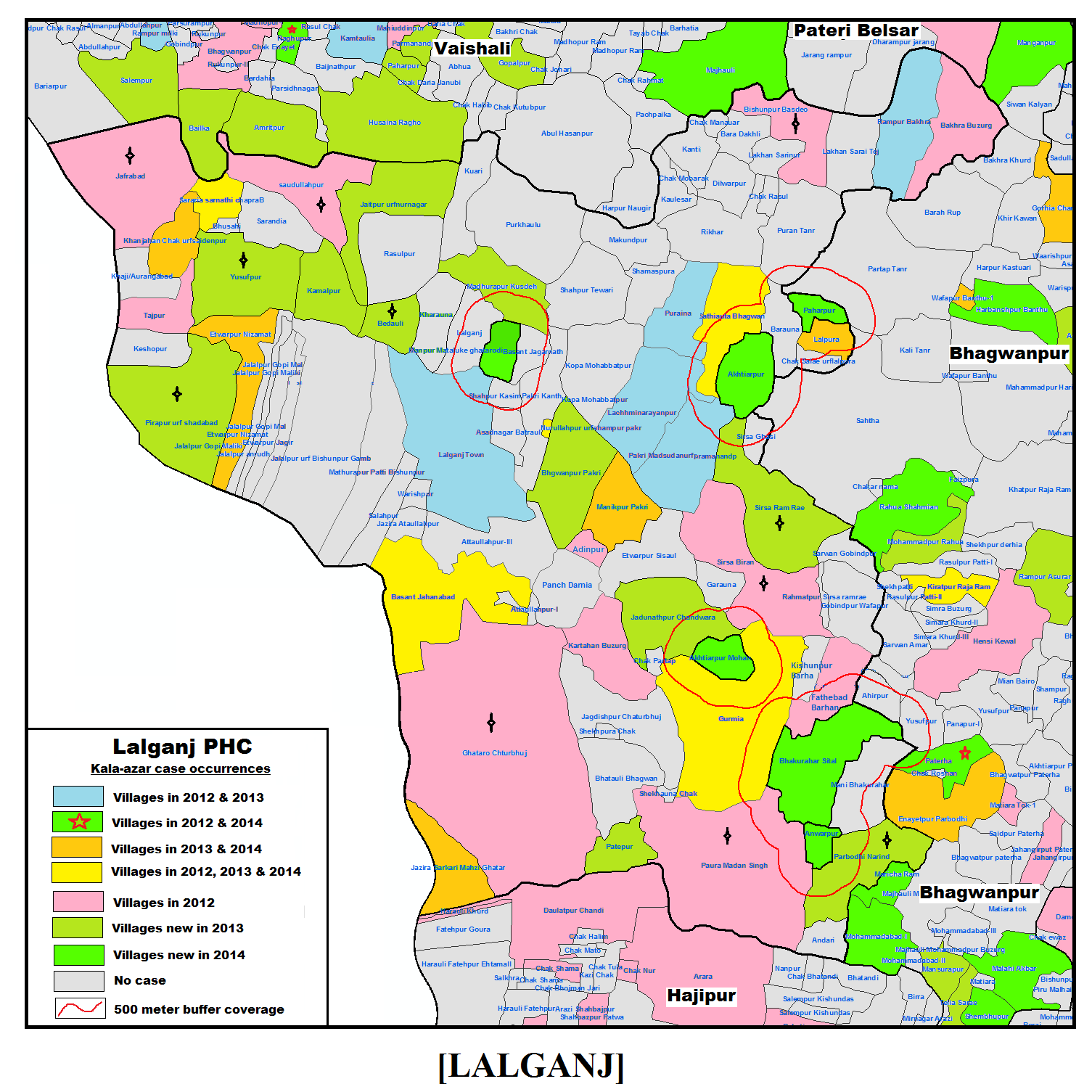


**L. Mahnar**


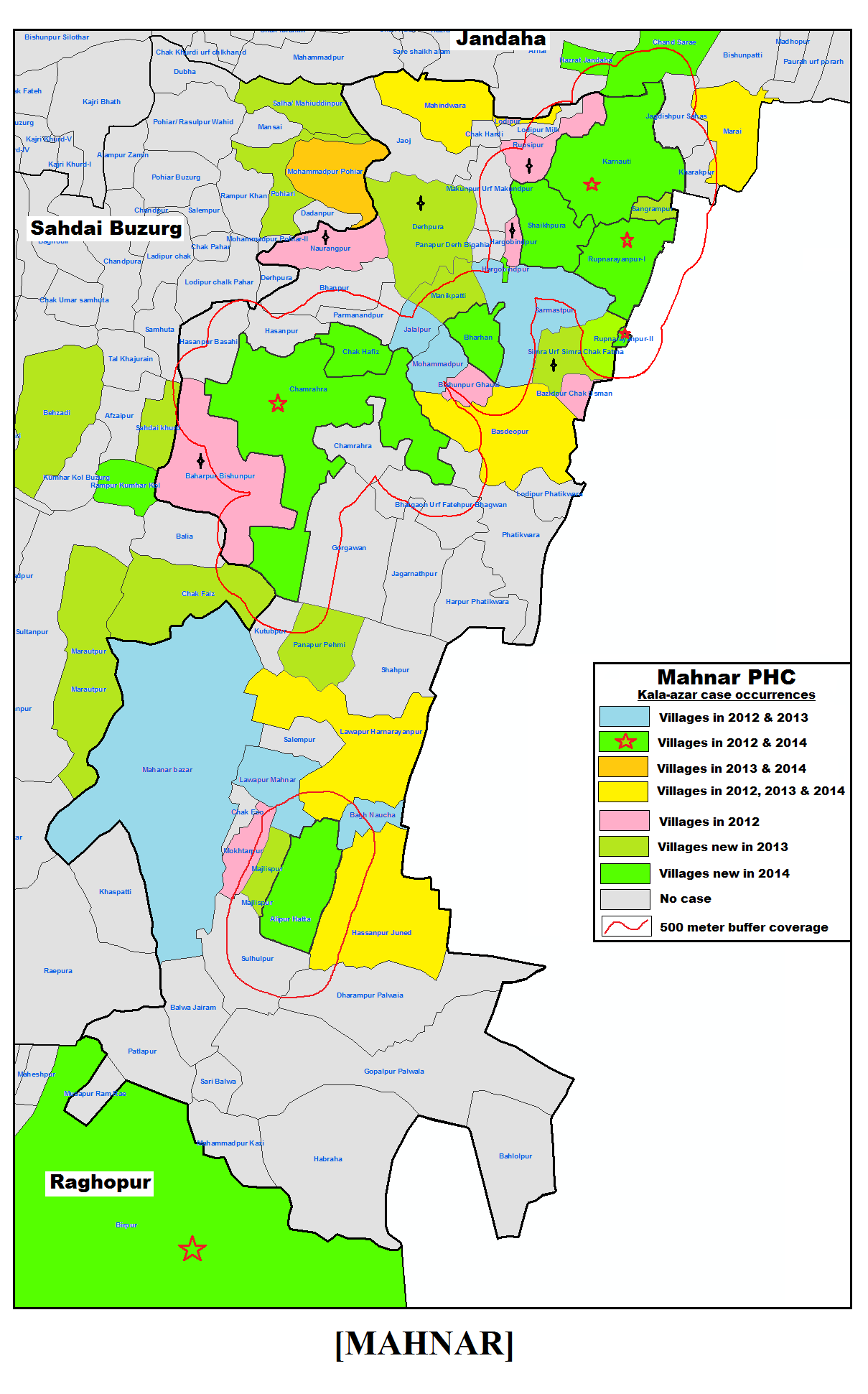


**L. Mahua**


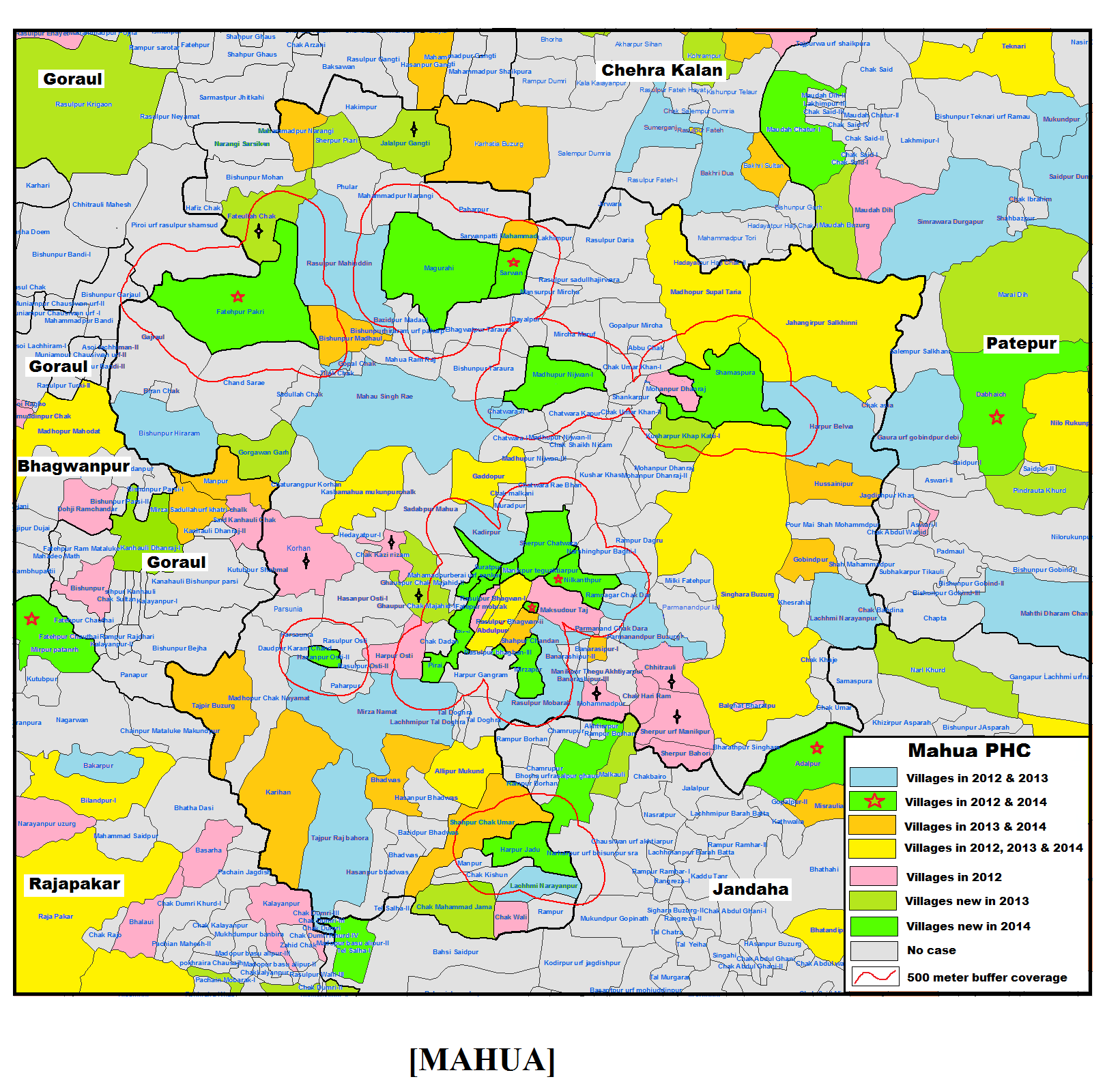


**M. Patepur**


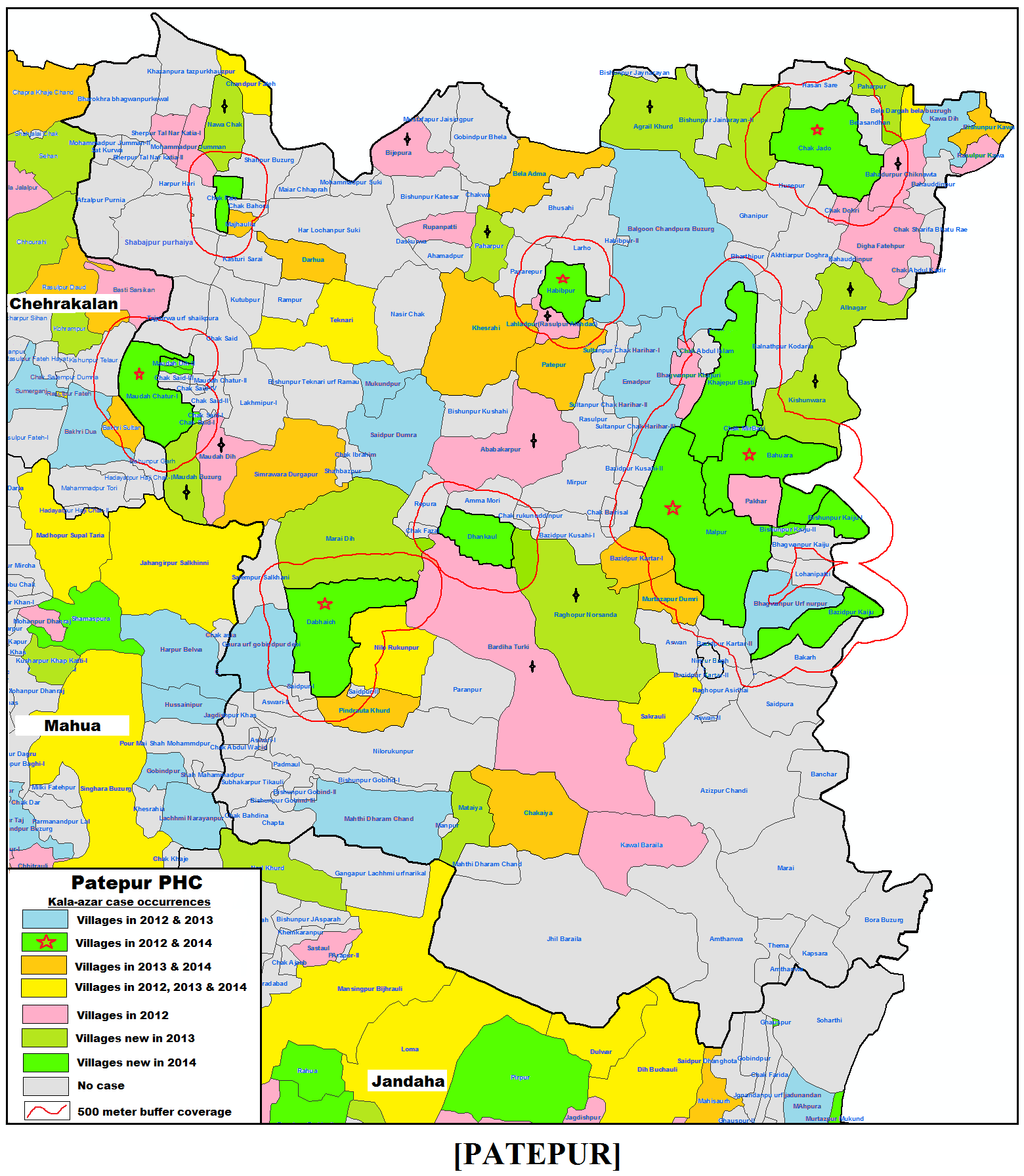


**N. Paterhi Belsar**


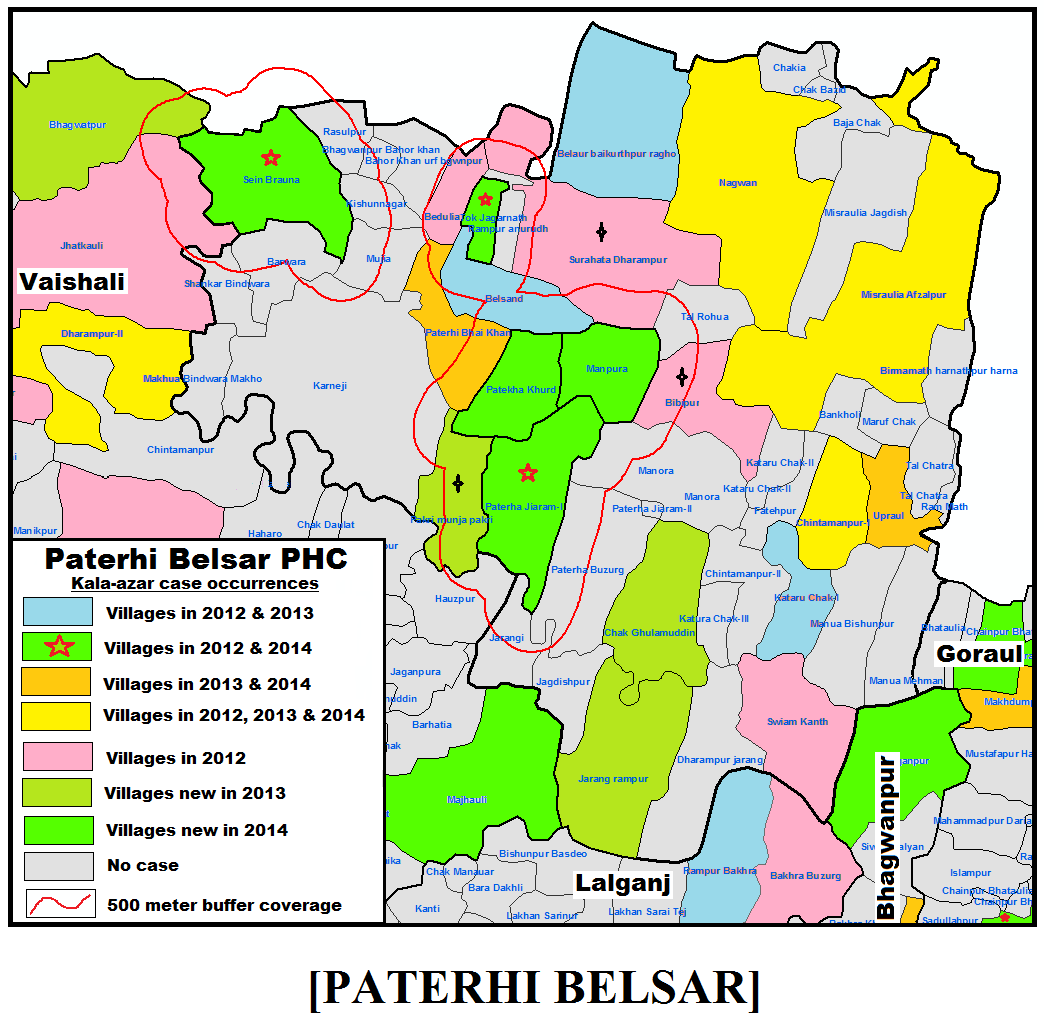


**M. Raghopur**
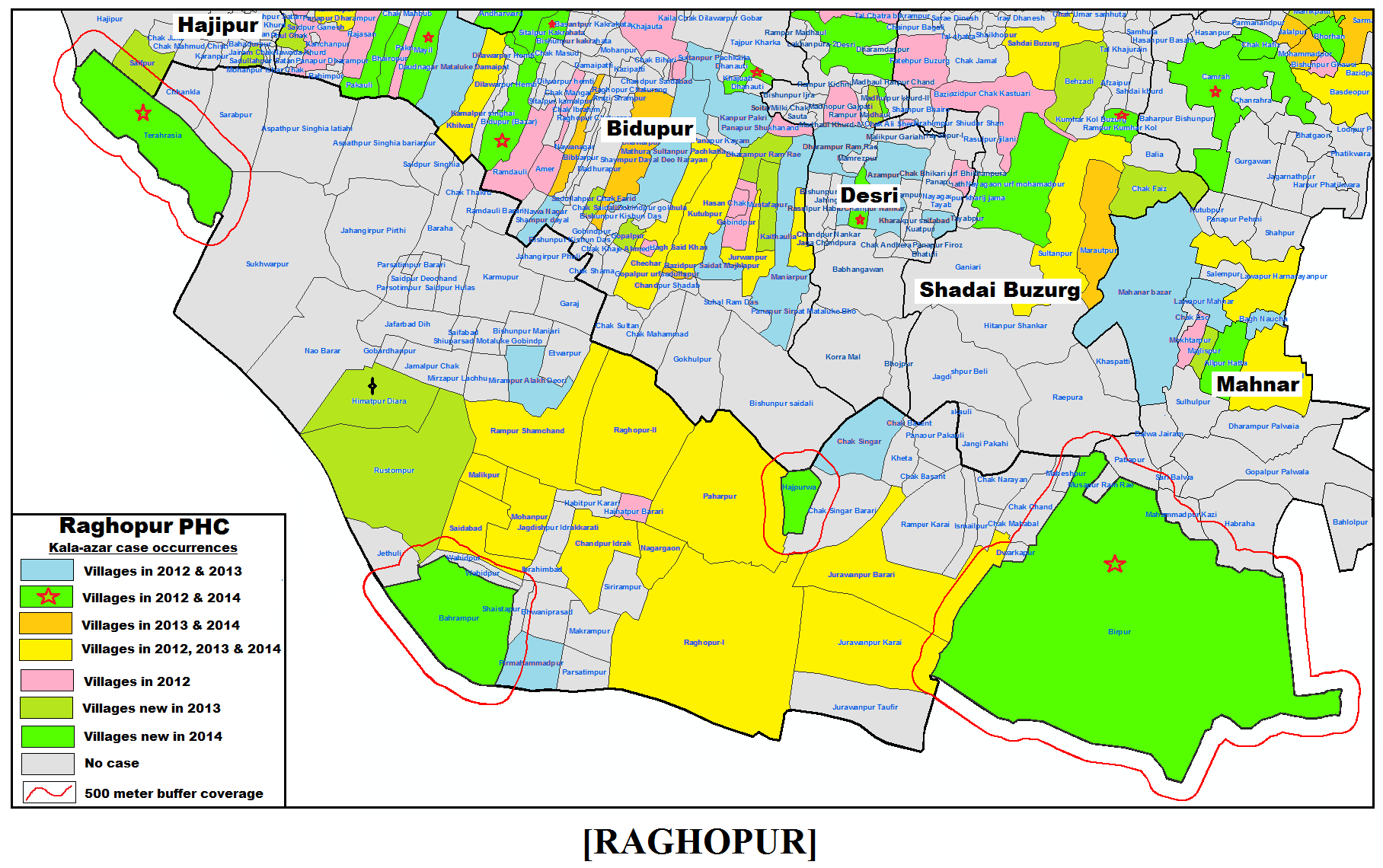


**N. Rajapakar**


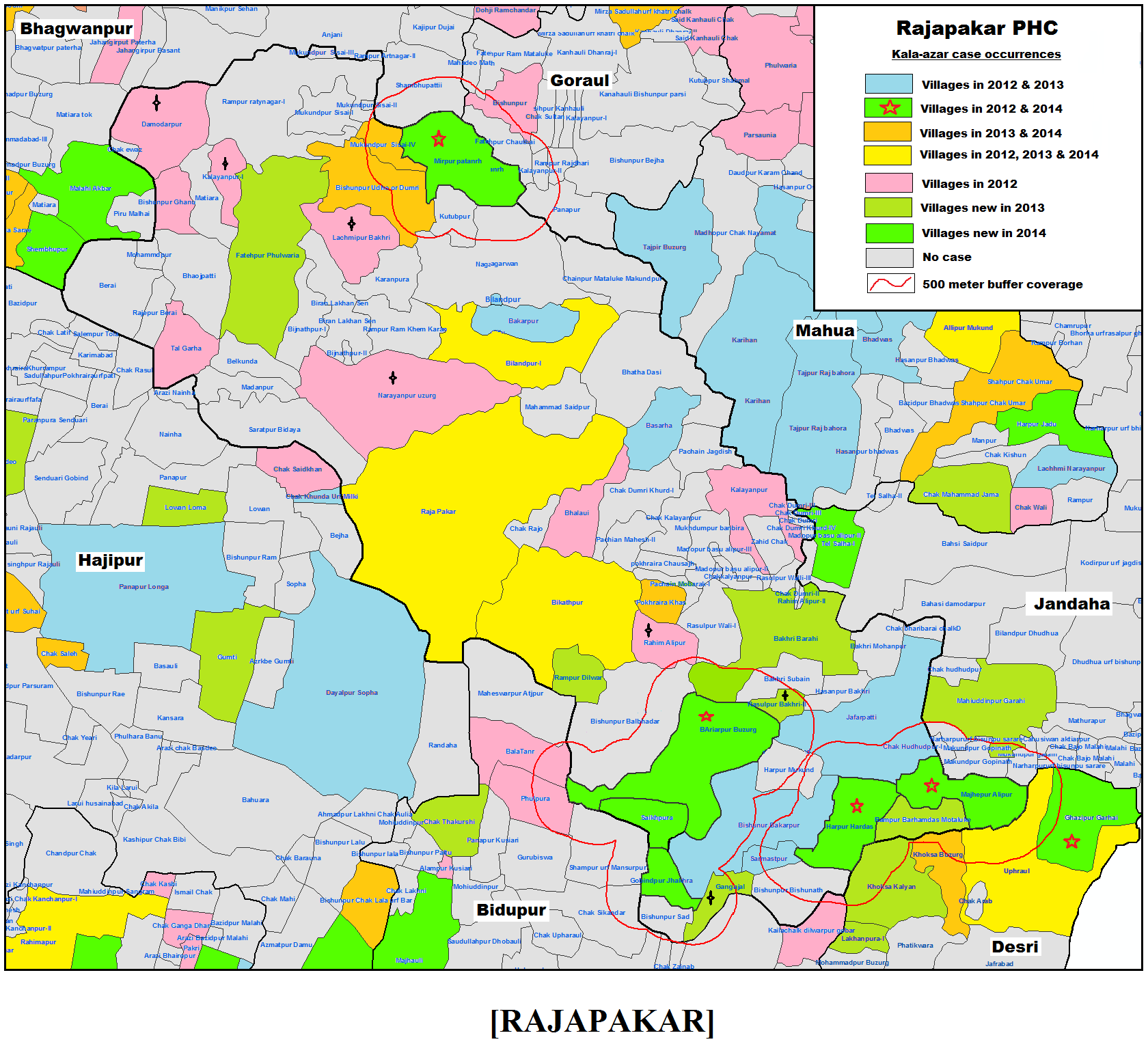


**O. Sahdai Buzurg**
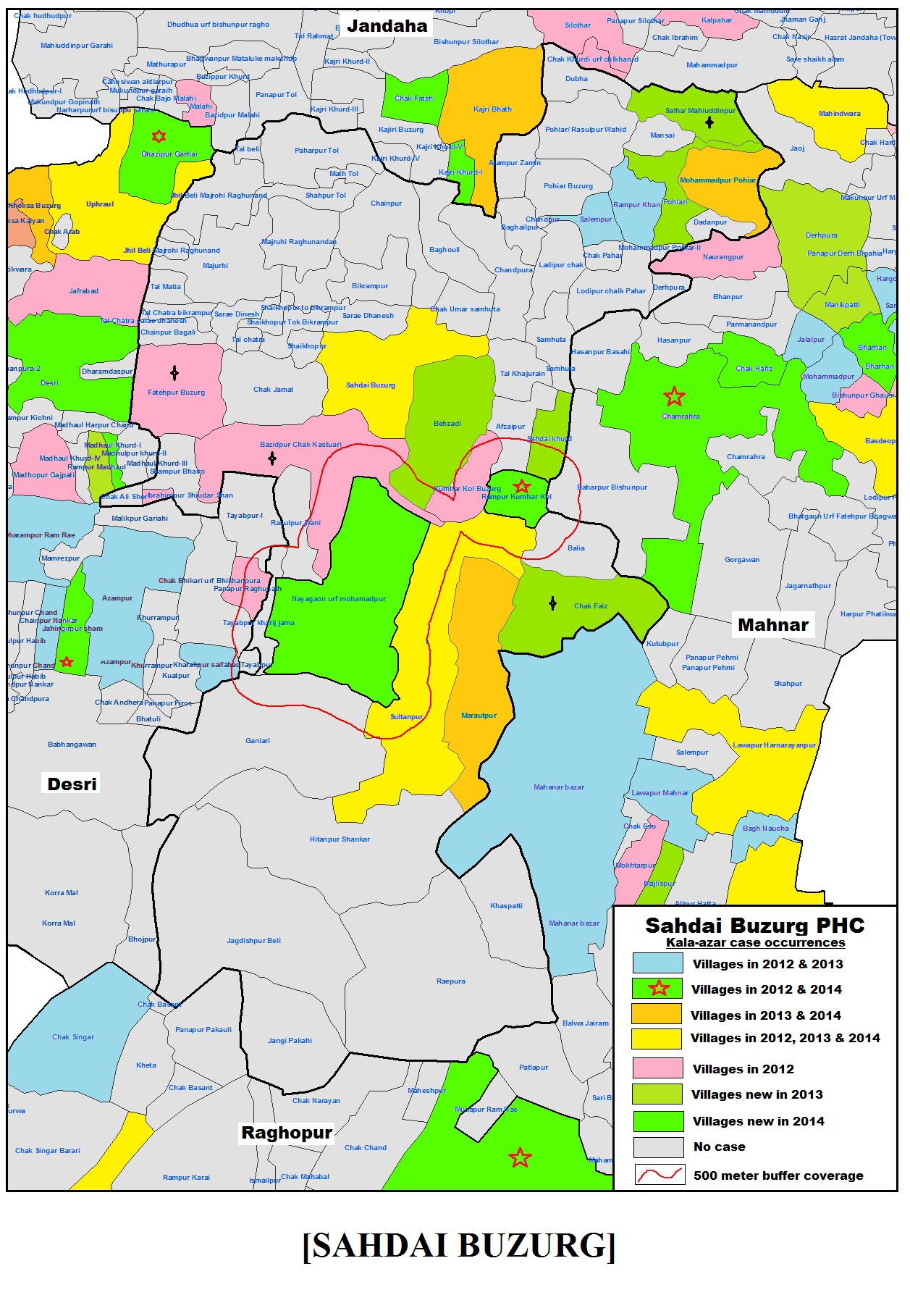


**P. Vaishali**
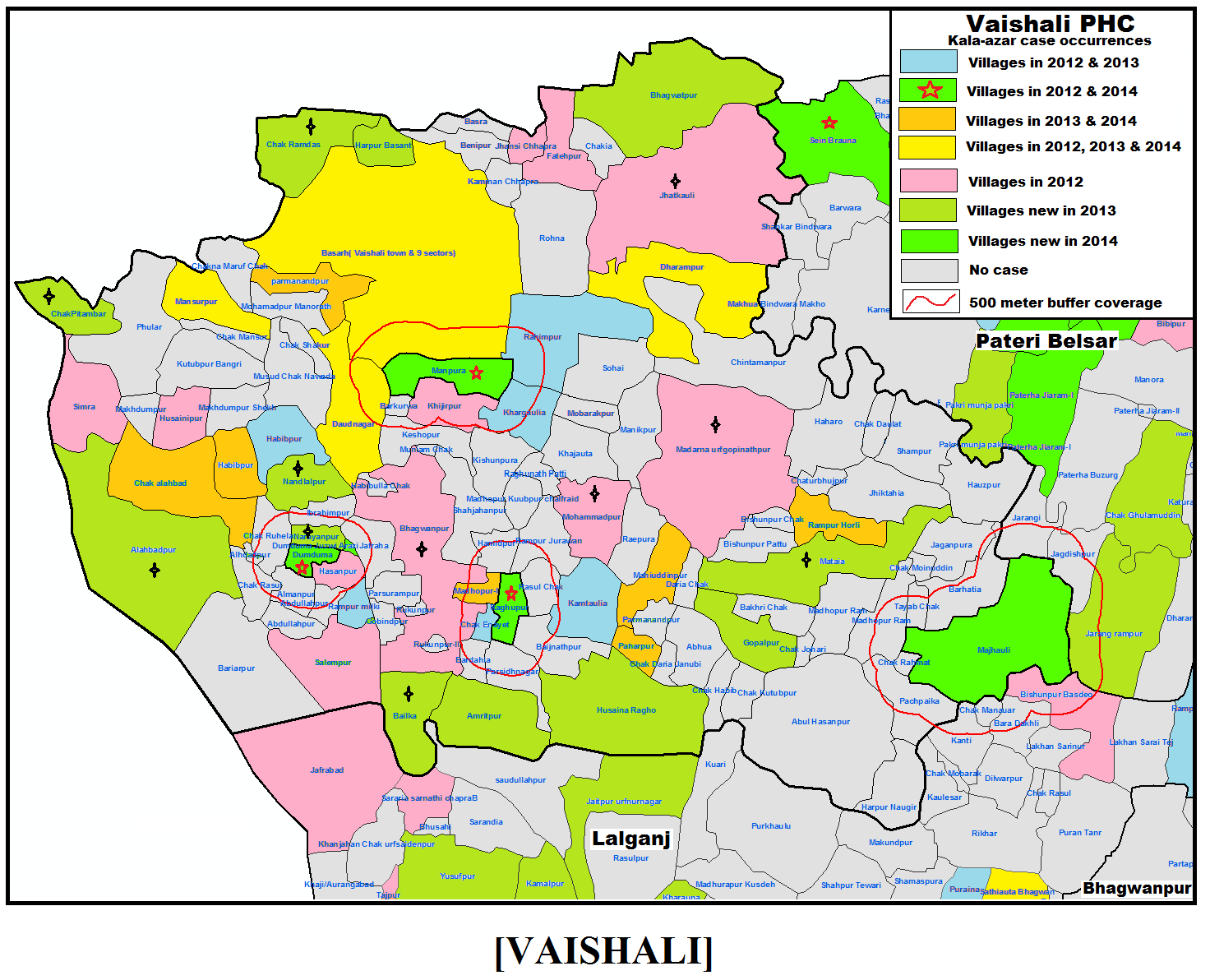

Supplement: S1 Appendix — An example of GIS based epidemiological mapping used for IRS-village selection at the block level (Panel ‘A-P’) of Vaishali district, Bihar (India). The red line around the new villages administrative boundary shows the nearest neighboring VL-endemic hot-spot and high-risk non-endemic villages within 500 m. A GIS-database built in the remote sensing project of ICMR-Rajendra Memorial Research Institute of Medical Sciences was used to create the maps in the figure. (DOCX) [file pntd.0008254.s004.docx]
