# Supplementary material for: Kala-azar elimination in a highly-endemic district of Bihar, India: A success story
Source: PLoS Negl Trop Dis. 2020 May 4;14(5):e0008254. doi: 10.1371/journal.pntd.0008254 (PMC7224556; doi:10.1371/journal.pntd.0008254)
Supplement: S2 Appendix — (DOCX) [file pntd.0008254.s005.docx]

**S2 Appendix. Details of different types IEC/BCC (Panel ‘A-M’) conducted during VL-vector control programme in Vaishali district, Bihar (India) during 2015-16.**

1. **Block level banner**


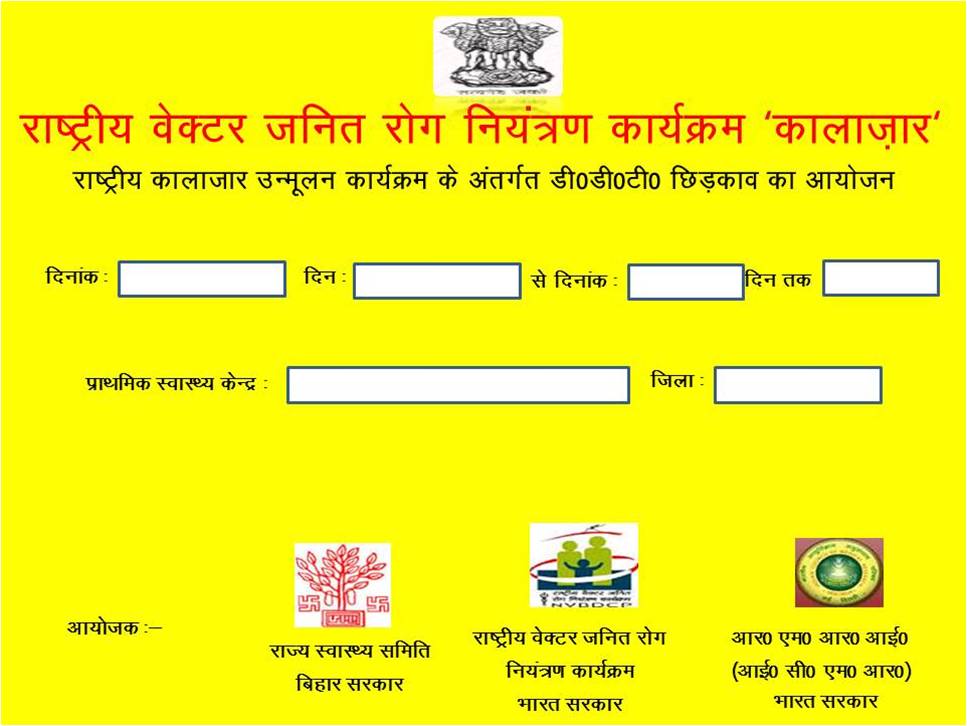


1. **Village level banner:**
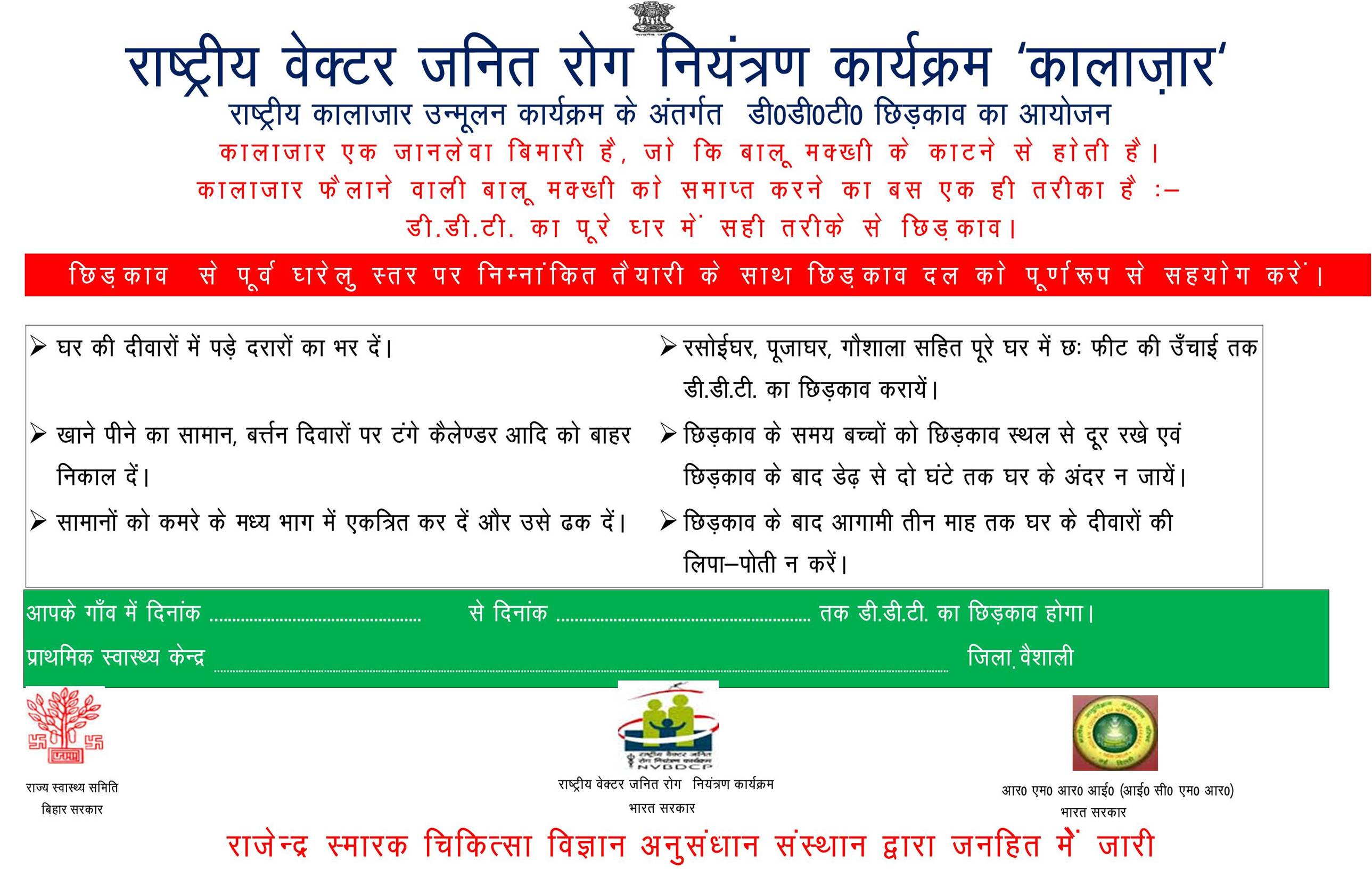

2. **Block level hoarding:**


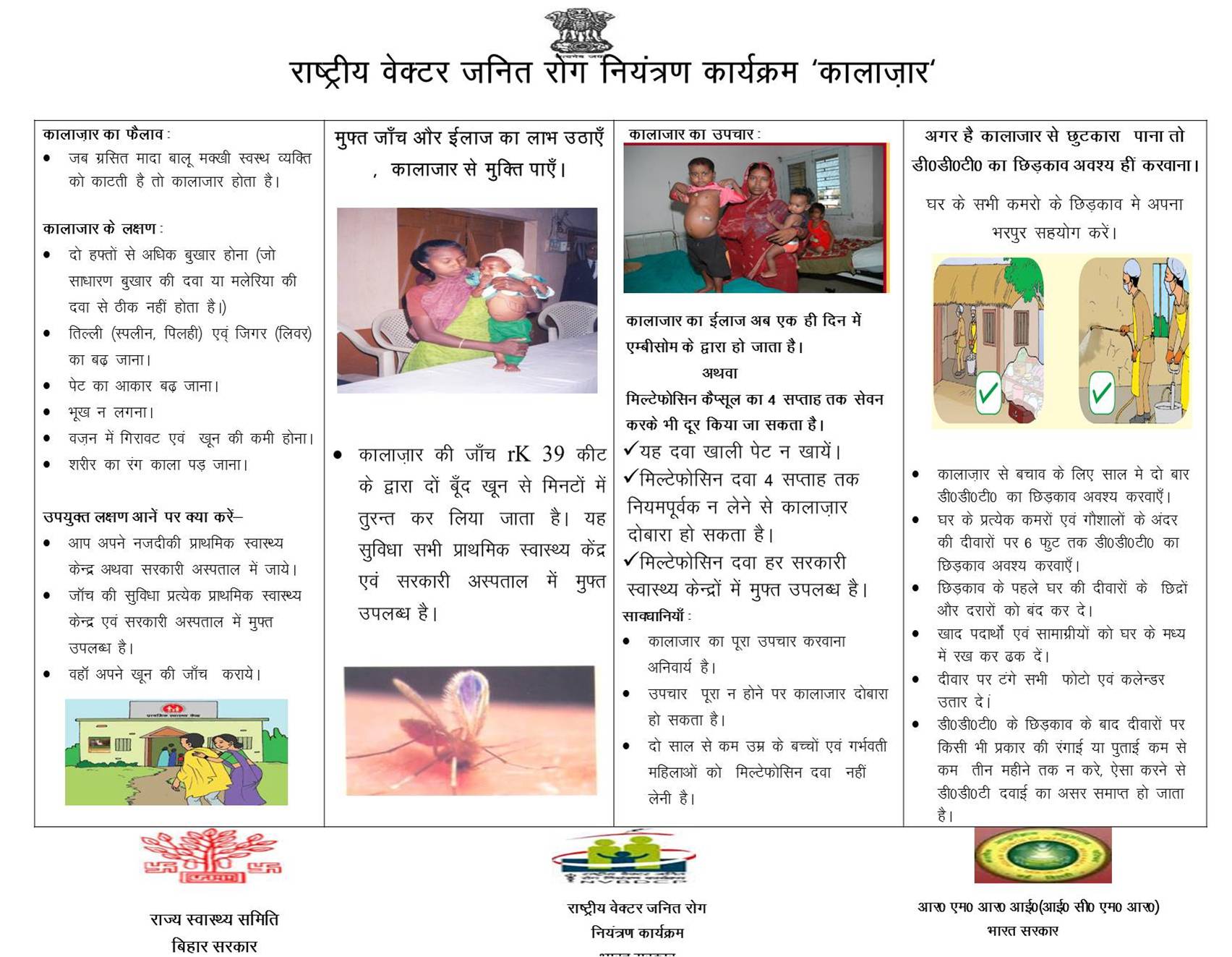


1. **Leaflet:**
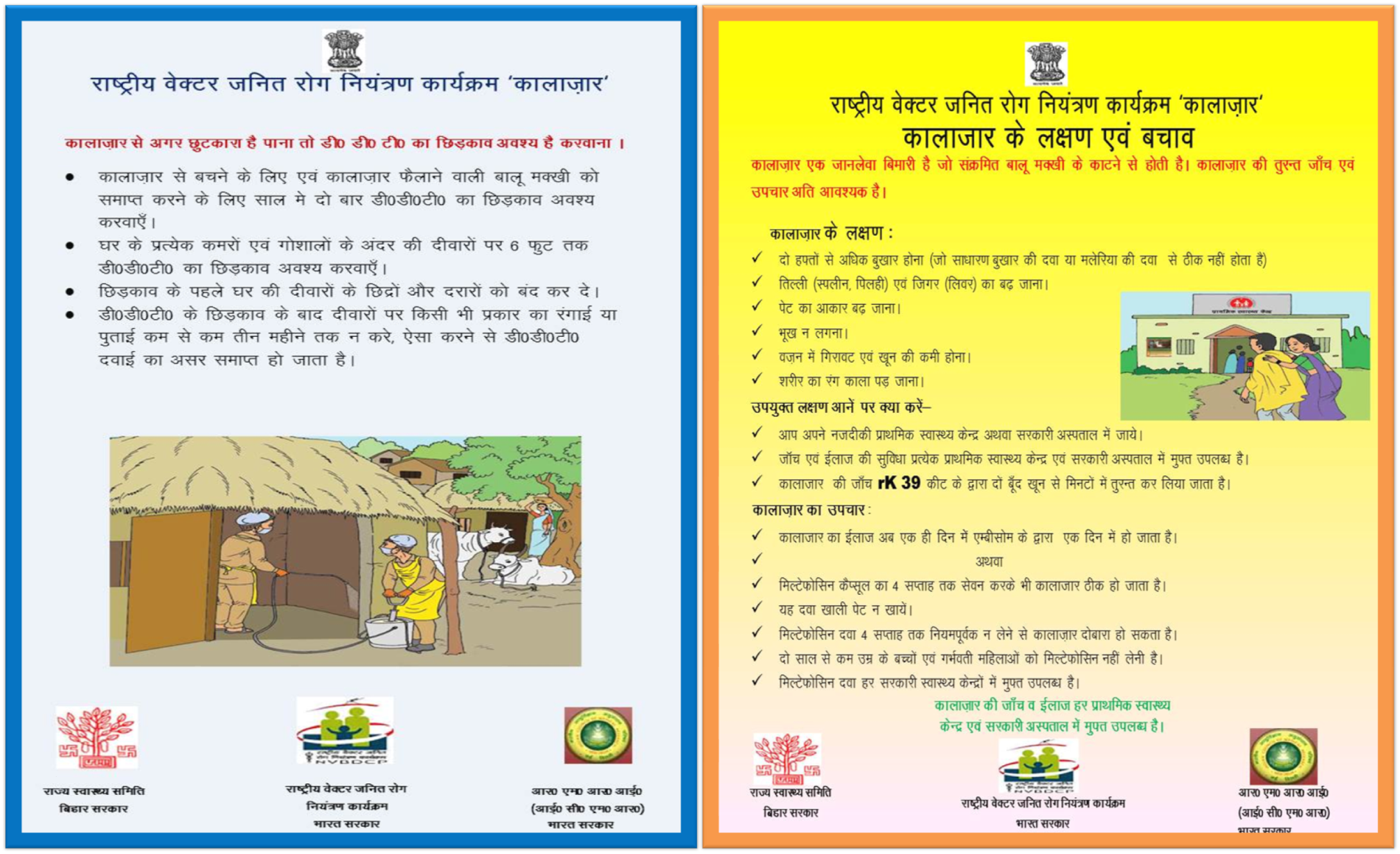

2. **Village level poster:**


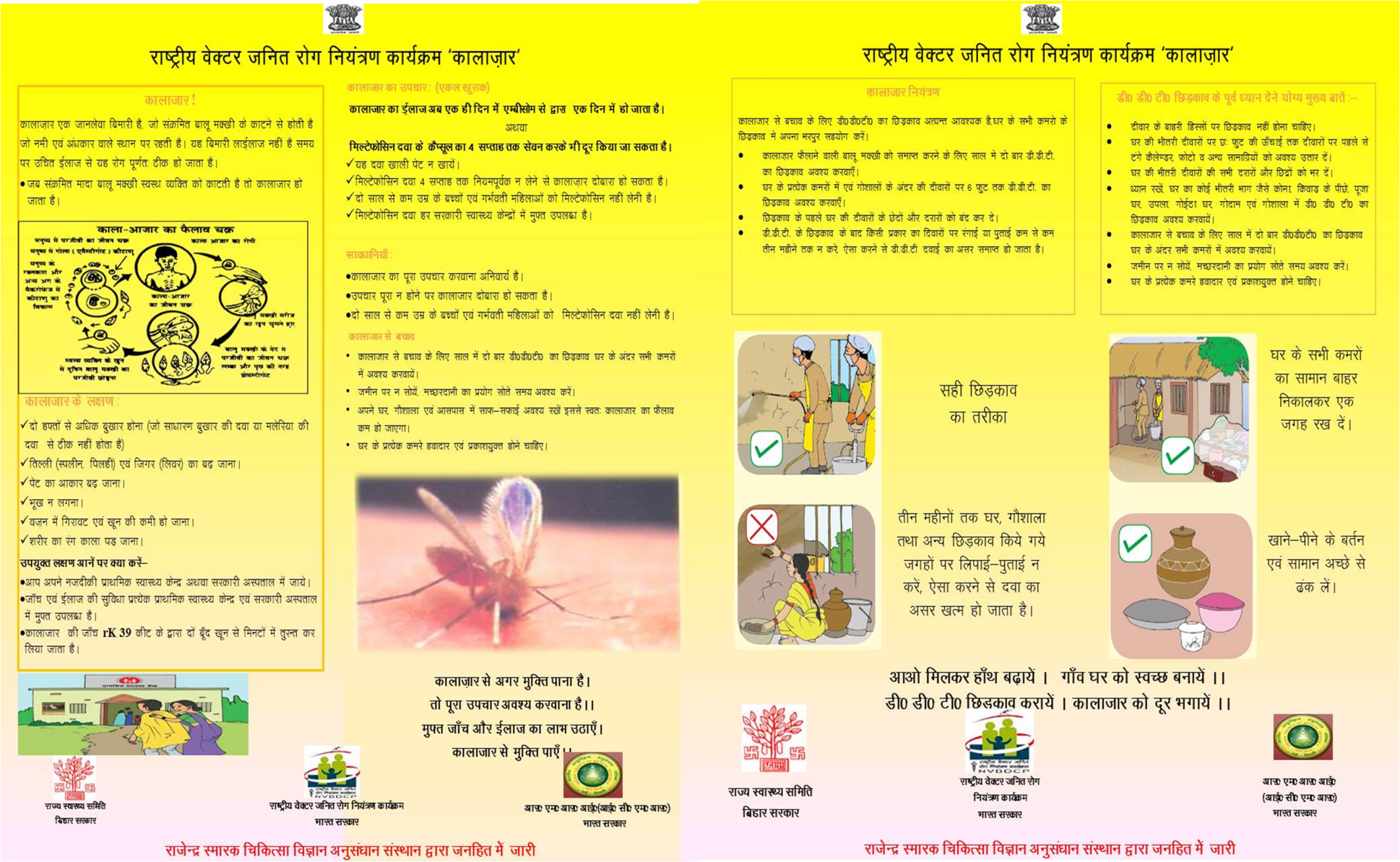


1. **Household level sticker type 1:**


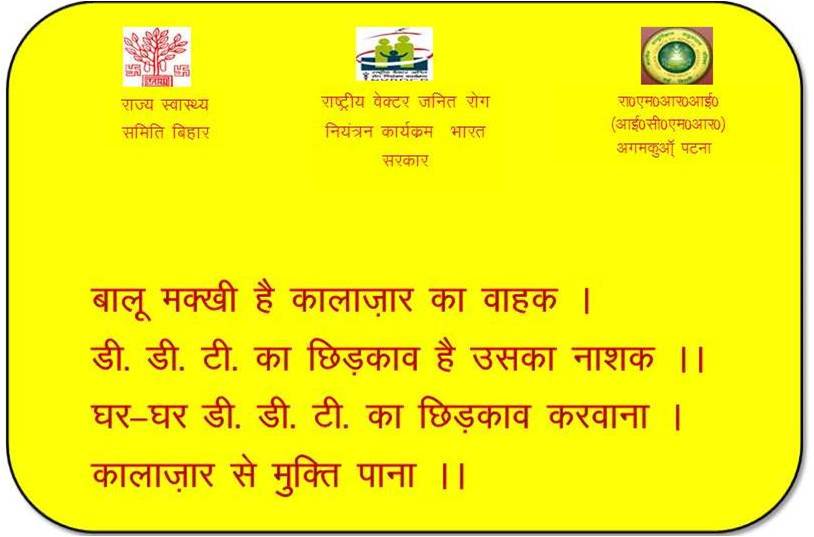


1. **Household level sticker type 2:**
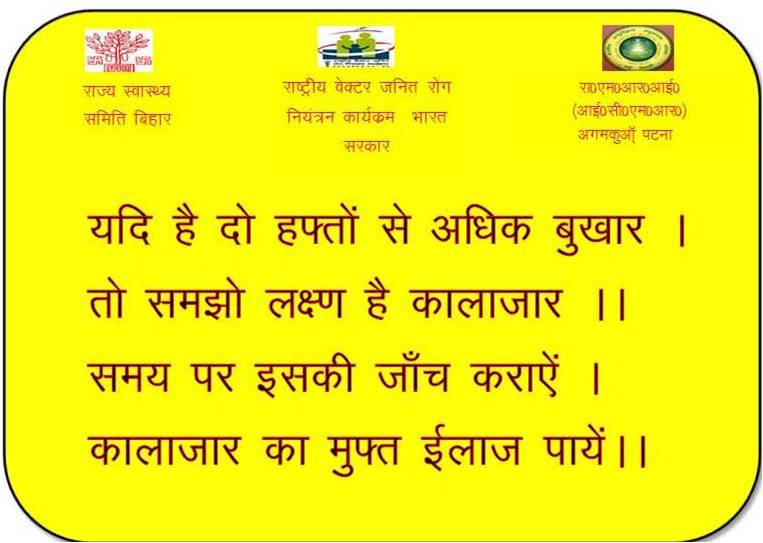

2. **Household level sticker type 3:**


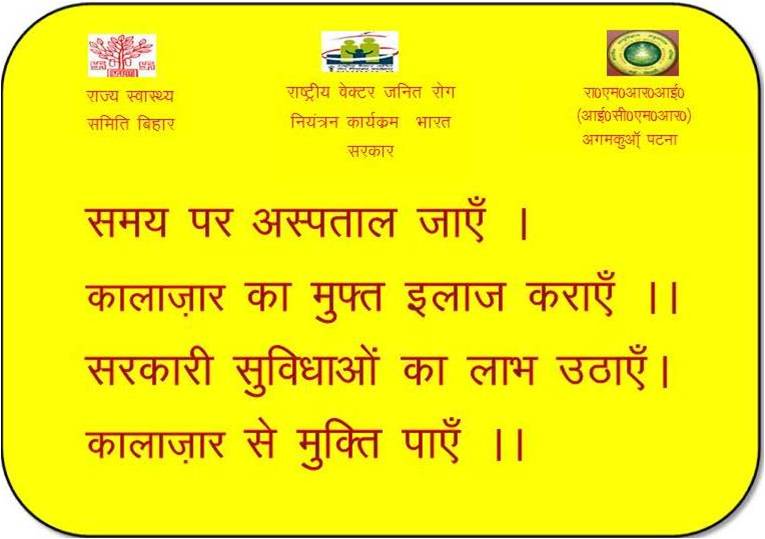


1. **Household level sticker type 4:**
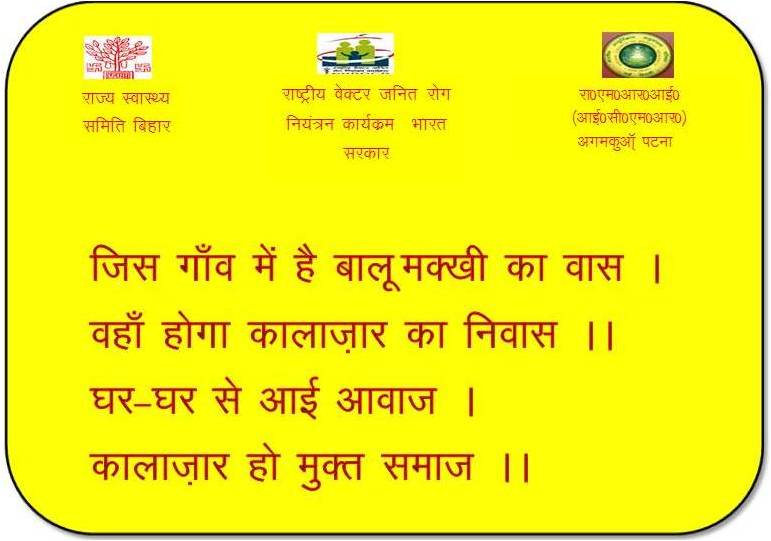

2. **Field photograph 1:**
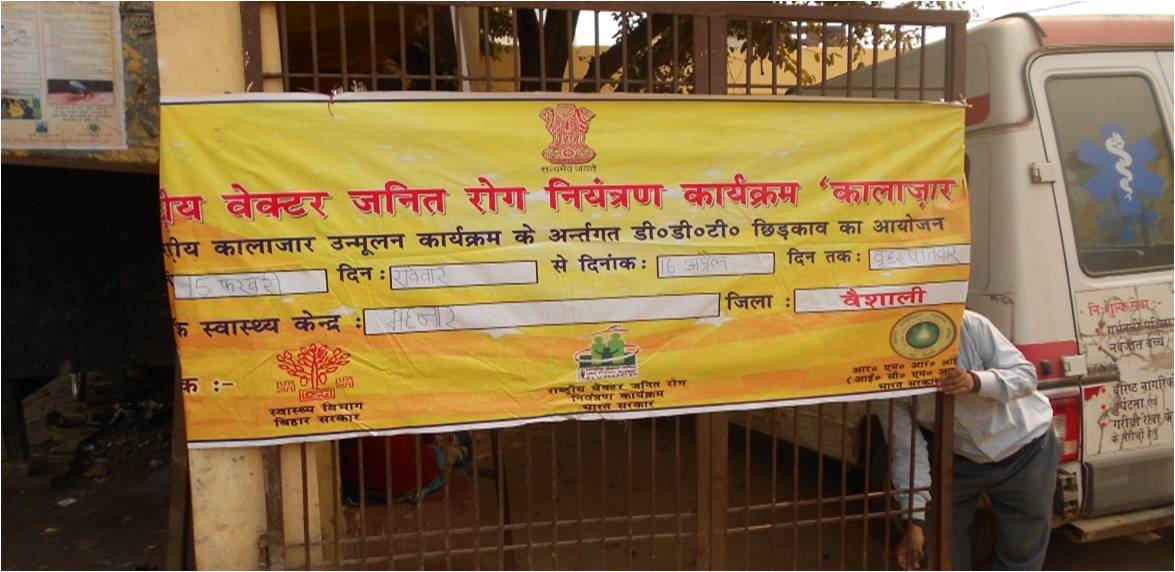

3. **Field photograph 2:**
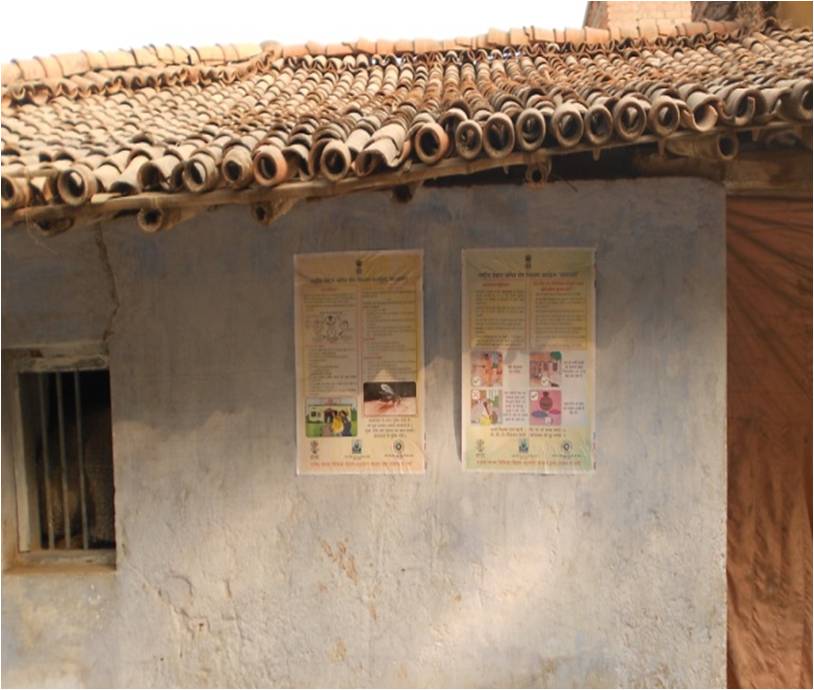

4. **Field photograph 3:**


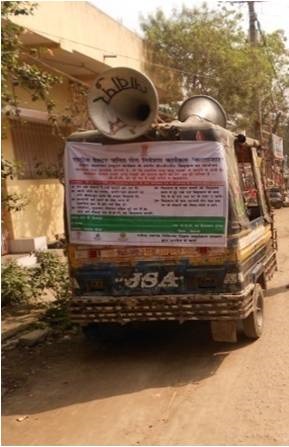


1. **Field photograph 4:**

**
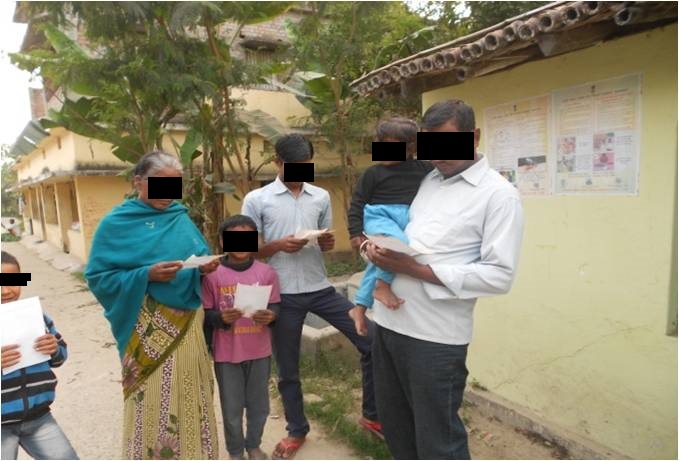
**
