# Supplementary material for: Kala-azar elimination in a highly-endemic district of Bihar, India: A success story
Source: PLoS Negl Trop Dis. 2020 May 4;14(5):e0008254. doi: 10.1371/journal.pntd.0008254 (PMC7224556; doi:10.1371/journal.pntd.0008254)
Supplement: S1 Table — (DOCX) [file pntd.0008254.s006.docx]

**S1 Table: IRS schedule of Kala-azar vector control programme during 2015 and 2016 in Vaishali district Bihar.**

| Date | IRS round(s) |
| --- | --- |
| **15^th^ February – 25^th^ April 2015** | 1^st^ |
| **17^th^ June – 4^th^ September 2015** | 2^nd^ |
| **16^th^ March – 7^th^ June 2016** | 1^st^ |
| **16^th^ August – 19^th^ November 2016** | 2^nd^ |
