# Supplementary material for: Kala-azar elimination in a highly-endemic district of Bihar, India: A success story
Source: PLoS Negl Trop Dis. 2020 May 4;14(5):e0008254. doi: 10.1371/journal.pntd.0008254 (PMC7224556; doi:10.1371/journal.pntd.0008254)
Supplement: S2 Table — (DOCX) [file pntd.0008254.s007.docx]

**S2 Table: Details of IEC/BCC materials distributed during 2015 and 2016 in Vaishali district, Bihar.**

| **Sl. No** | **Name of the PHCs** | **Number of villages and ward covered** | **Banner-I (Block level)** | **Banner-II**  **(village level)** | **Hoarding** | **Poster** | **Leaflet** | **Sticker** |
| --- | --- | --- | --- | --- | --- | --- | --- | --- |
| **1** | **Hajipur** | 160 | 5 | 54 | 7 | 6400 | 12800 | 6400 |
| **2** | **Bidupur** | 95 | 4 | 39 | 4 | 3800 | 7600 | 3800 |
| **3** | **Goraul** | 53 | 2 | 21 | 2 | 2120 | 4240 | 2120 |
| **4** | **Chehrakala** | 40 | 2 | 18 | 2 | 1600 | 3200 | 1600 |
| **5** | **Bhagwanpur** | 63 | 2 | 24 | 2 | 2520 | 5040 | 2520 |
| **6** | **Vaishali** | 86 | 3 | 27 | 3 | 3440 | 6880 | 3440 |
| **7** | **Patedhi Belsar** | 40 | 2 | 15 | 2 | 1600 | 3200 | 1600 |
| **8** | **Lalganj** | 86 | 3 | 36 | 3 | 3440 | 6880 | 3440 |
| **9** | **Mahua** | 114 | 4 | 39 | 5 | 4560 | 9120 | 4560 |
| **10** | **Rajapakar** | 54 | 2 | 21 | 2 | 2160 | 4320 | 2160 |
| **11** | **Jandaha** | 90 | 3 | 42 | 4 | 3600 | 7200 | 3600 |
| **12** | **Mahnar** | 64 | 2 | 30 | 4 | 2560 | 5120 | 2560 |
| **13** | **Sahdei Bujurg** | 49 | 2 | 18 | 2 | 1960 | 3920 | 1960 |
| **14** | **Desari** | 34 | 2 | 15 | 2 | 1360 | 2720 | 1360 |
| **15** | **Patepur** | 124 | 4 | 63 | 6 | 4960 | 9920 | 4960 |
| **16** | **Raghopur** | 44 | 2 | 33 | 2 | 1760 | 3520 | 1760 |
| **Total** | | 1196 | 44 | 495 | 52 | 47,840 | 95,680 | 47,840 |
