# Supplementary material for: Kala-azar elimination in a highly-endemic district of Bihar, India: A success story
Source: PLoS Negl Trop Dis. 2020 May 4;14(5):e0008254. doi: 10.1371/journal.pntd.0008254 (PMC7224556; doi:10.1371/journal.pntd.0008254)
Supplement: S3 Table — (DOCX) [file pntd.0008254.s008.docx]

**S3 Table: Definition of refused, partially- and fully-sprayed, and missed houses, false stenciling, and doubling in house serial number during IRS-based VL-vector control programme in Vaishali district, Bihar, India.**

| **Word detail** | **Definition** |
| --- | --- |
| **Refused houses** | Houses in an intervention village those owners refused permission to IRS. |
| **Partially sprayed house** | A house in an intervention village where spraying is not done in all rooms (one or, more rooms remained unsprayed including kitchen, toilet, and animal shelters) or room walls are not covered up to the 1.82 m (or 6 feet) height. |
| **Fully sprayed house** | A house in an intervention village where IRS is done up to the 1.82 m (or 6 feet) height in all walls of the available rooms including kitchen, toilet, and animal shelters. |
| **Missed house** | A house in a village which is not attained by IRS team during spraying. |
| **False** **stenciling** | A house in a village which is actually unsprayed during IRS, but spraymen has noted down the householder’s name under sprayed houses in IRS-register and provided a spray ID against the house and also stencil a mark on the house wall. |
| **HH doubling** | Spray status of a single house noted down and stencilled with multiple IDs. |
