# Supplementary material for: Kala-azar elimination in a highly-endemic district of Bihar, India: A success story
Source: PLoS Negl Trop Dis. 2020 May 4;14(5):e0008254. doi: 10.1371/journal.pntd.0008254 (PMC7224556; doi:10.1371/journal.pntd.0008254)
Supplement: S4 Table — (DOCX) [file pntd.0008254.s009.docx]

**S4 Table: The total number of villages targeted and covered during the first and second rounds of IRS in 2015 and 2016 in the Vaishali District, Bihar.**

| **Year (s)** | **Average Days per IRS Round** | **Total Villages in Vaishali** | **Villages Targeted** | | **Total Villages Targeted**  **(% of Total Villages)** | **Total Villages Covered**  **(% of Total Targeted Villages)** |
| --- | --- | --- | --- | --- | --- | --- |
|  |  |  | **Endemic (n)** | **Non-Endemic Peripheral (n)** |  |  |
| **2015** | **First Round (59.3)** | 1542 | 662 | 434 | 1,096 (71.1%) | 10,96 (100%) |
|  | **Second Round (58.5)** |  | 645 | 433 | 1,078 (69.9%) | 1,078 (100%) |
| **2016** | **First Round (57.8)** |  | 580 | 415 | 995 (64.5%) | 995 (100%) |
|  | **Second Round (58.2)** |  | 611 | 407 | 1,018 (66%) | 1,018 (100%) |
| **Average (58.4)** | | | 624.5 | 422.3 | 1,046.8 (67.9%) | 1,046.8 (100%) |
