# Supplementary material for: Kala-azar elimination in a highly-endemic district of Bihar, India: A success story
Source: PLoS Negl Trop Dis. 2020 May 4;14(5):e0008254. doi: 10.1371/journal.pntd.0008254 (PMC7224556; doi:10.1371/journal.pntd.0008254)
Supplement: S5 Table — (DOCX) [file pntd.0008254.s010.docx]

**S5 Table: Details of population and HH data targeted, covered, and sprayed during the first and second rounds of IRS in 2015 and 2016 in the Vaishali District, Bihar.**

| **Year (s)** | **IRS Round (S)** | **Total Population Targeted** | **Total HHs Targeted** | **Total Population Covered (%)** | **Total Refused HHs (%)** | **Total Locked HHs (%)** | **Total HHs Sprayed (%)** | **Total HHs Fully-Sprayed (%)** | **Total HHs Partially-Sprayed (%)** |
| --- | --- | --- | --- | --- | --- | --- | --- | --- | --- |
|  |  |  |  |  |  |  |  |  |  |
| **2015** | **First Round** | 2,989,566 | 492,111 | 2,873,443 (96.1%) | 11,958 (2.4%) | 13,112 (2.7%) | 467,041 (94.9%) | 423,939 (90.8%) | 43,102 (9.2%) |
|  | **Second Round** | 3,193,216 | 516,759 | 3,098,813 (97%) | 7,344 (1.4%) | 12,408 (2.4%) | 497,007 (96.2%) | 468,124 (94.2%) | 28,883 (5.8%) |
| **2016** | **First Round** | 3,053,130 | 533,822 | 2,988,195 (97.9%) | 5,041 (0.9%) | 9,460 (1.8%) | 519,321 (97.3%) | 492,563 (94.8%) | 26,758 (5.2%) |
|  | **Second Round** | 3,074,819 | 543,843 | 3,034,701 (98.7%) | 4,722 (0.9%) | 4,965 (0.9%) | 534,157 (98.2%) | 513,897 (96.2%) | 20,260 (3.8%) |
| **Average** | | 3,077,683 | 521,634 | 2,998,788 (97.4%) | 7,266 (1.4%) | 9,986 (1.9%) | 504,382 (96.7%) | 474,631 (94.1%) | 29,751 (5.9%) |
