# Supplementary material for: Kala-azar elimination in a highly-endemic district of Bihar, India: A success story
Source: PLoS Negl Trop Dis. 2020 May 4;14(5):e0008254. doi: 10.1371/journal.pntd.0008254 (PMC7224556; doi:10.1371/journal.pntd.0008254)
Supplement: S6 Table. Details of villages, population, and HHs data targeted and covered by FS during IRS in 2015–2016 in the Vaishali District, Bihar — (DOCX) [file pntd.0008254.s011.docx]

**S6 Table: Details of villages, population, and HHs data targeted and covered by FS during IRS in 2015-2016 in the Vaishali District, Bihar.**

| **Year(s)** | **Total Villages Targeted (N)** | **Total Villages Covered (%)** | **Total HHs Covered** | **Total Population Covered** | **Average HHs Covered per FS** | **Average Population Covered per FS** | **Villages Reporting VL Cases after FS at End of the Year (%)** | **Villages Not Covered by FS (%)** | **Non-FS Villages Reporting VL Cases at End of the Year (%)** |
| --- | --- | --- | --- | --- | --- | --- | --- | --- | --- |
| **2015** | 54 | 47 (87%) | 3,063 | 17,199 | 65.2 | 365.9 | 14 (29.8%) | 7 (13%) | 5 (71.4%) |
| **2016** | 39 | 34 (87.2%) | 2,132 | 11,742 | 62.7 | 345.4 | 9 (26.5%) | 5 (12.8%) | 4 (80%) |
| **Average** | 46.5 | 40.5 (87.1%) | 2,597.5 | 14,470.5 | 63.9 | 355.6 | 11.5 (28.1%) | 6 (12.9%) | 4.5 (75%) |
