# Supplementary material for: Kala-azar elimination in a highly-endemic district of Bihar, India: A success story
Source: PLoS Negl Trop Dis. 2020 May 4;14(5):e0008254. doi: 10.1371/journal.pntd.0008254 (PMC7224556; doi:10.1371/journal.pntd.0008254)
Supplement: S7 Table — (DOCX) [file pntd.0008254.s012.docx]

**S7 Table: The number of households that refused IRS and the conversion rate to sprayed status by squad monitors, ASHAs, KTSs, and PHC supervisors during IRS in the Vaishali District, Bihar, in 2015-2016.**

| **IRS Round(s)** | **HHs that Refused IRS by Spraymen** | **HHs that Accepted IRS after Visit (%)** | **HHs Converted after Refusing to be Sprayed by Different Teams** | | | |
| --- | --- | --- | --- | --- | --- | --- |
|  |  |  | **By Squad Monitors** | **By ASHAs** | **By KTSs** | **By PHC Supervisors** |
| **First Round 2015** | 959,038 | 947,080 (98.8%) | 477,328 (50.4%) | 163,845 (17.3%) | 53,461 (5.6%) | 252,446 (26.7%) |
| **Second Round 2015** | 108,887 | 101,543 (93.3%) | 53,310 (52.5%) | 16,653 (16.4%) | 7,730 (7.6%) | 23,850 (23.5%) |
| **First Round 2016** | 90,976 | 85,935 (94.5%) | 47,608 (55.4%) | 13,234 (15.4%) | 5,380 (6.3%) | 19,713 (22.9%) |
| **Second Round 2016** | 78,312 | 73,590 (94%) | 42,461 (57.4%) | 10,744 (14.6%) | 5,223 (7.1%) | 15,162 (20.6%) |
| **Average** | 309,303 | 302,037 (97.7%) | 155,177 (54%) | 51,119 (15.9%) | 17,935 (6.7%) | 77,806 (23.4%) |
