# Supplementary material for: Kala-azar elimination in a highly-endemic district of Bihar, India: A success story
Source: PLoS Negl Trop Dis. 2020 May 4;14(5):e0008254. doi: 10.1371/journal.pntd.0008254 (PMC7224556; doi:10.1371/journal.pntd.0008254)
Supplement: S8 Table — (DOCX) [file pntd.0008254.s013.docx]

**S8 Table: Household level post-IRS survey report regarding the IRS activities and quality spraying in the villages of the Vaishali District, Bihar, during 2015-2016.**

| **IRS Round(s)** | **Villages Surveyed (n)** | **HHs Surveyed (n)** | **Details of Stencil Status on Wall** | | | | **HHs Washed Out or Repainted (%)** | **Details of IRS Quality Survey** | | | | | **Villages with Missed HHs (%)** |
| --- | --- | --- | --- | --- | --- | --- | --- | --- | --- | --- | --- | --- | --- |
|  |  |  | **Total False Stenciled HHs (%)** | **Partially Sprayed HHs Stenciled as Fully Sprayed (%)** | **Unsprayed HHs Stenciled as Partially/Fully Sprayed (%)** | **HHs with Double/Multiple Stencils (%)** |  | **HHs Passed IRS Evidence on Wall (%)** | **HHs Uniformly Sprayed (%)** | **HHs Patchy Sprayed (%)** | **HHs Sprayed up to 1.8 m Height (%)** | **HHs Sprayed <1.8 m Height (%)** |  |
| **First Round 2015** | 240 | 7,200 | 77 (1.1%) | 58 (75.3%) | 19 (24.7%) | 27 (0.4%) | 37 (0.5%) | 7,144 (99.2%) | 5,582 (78.1%) | 1,562 (21.9%) | 6,547 (91.6%) | 597 (8.4%) | 8 (3.3%) |
| **Second Round 2015** | 240 | 7,200 | 23 (0.3%) | 18 (78.3%) | 5 (21.7%) | 9 (0.1%) | 16 (0.2%) | 7,185 (99.8%) | 6,997 (97.4%) | 188 (2.6%) | 7,012 (97.6%) | 173 (2.4%) | 1 (0.4%) |
| **First Round 2016** | 240 | 7,200 | 4 (0.1%) | 3 (75%) | 1 (25%) | 6 (0.1%) | 2 (0.0%) | 7,197 (100%) | 6,919 (96.1%) | 278 (3.9%) | 7,161 (99.5%) | 36 (0.5%) | 0 (0.0%) |
| **Second Round 2016** | 240 | 7,200 | 0 (0.0%) | 0 (0.0%) | 0 (0.0%) | 2 (0.03%) | 1 (0.0%) | 7,199 (100%) | 6,972 (96.8%) | 227 (3.2%) | 7,182 (99.8%) | 17 (0.2%) | 0 (0.0%) |
| **Average** | 240 | 7,200 | 26 (0.4%) | 19.8 (76%) | 6.2 (24%) | 11 (0.2%) | 14 (0.2%) | 7,181.3 (99.7%) | 6,617.5 (92.1%) | 563.8 (7.9%) | 6,975.5 (97.1%) | 205.8 (2.9%) | 2.3 (0.9%) |
