# Supplementary material for: Kala-azar elimination in a highly-endemic district of Bihar, India: A success story
Source: PLoS Negl Trop Dis. 2020 May 4;14(5):e0008254. doi: 10.1371/journal.pntd.0008254 (PMC7224556; doi:10.1371/journal.pntd.0008254)
Supplement: S9 Table — (DOCX) [file pntd.0008254.s014.docx]

**S9 Table: Details of the damaged, faulty, and missing equipment repaired or newly provided during IRS in the Vaishali District, Bihar, in 2015-2016.**

| **IRS Round(s)** | **Pumps** | **Buckets** | **Insecticide Measuring Mugs** | **Water Measuring Mugs** | **Measuring Cylinders** | **Nozzle Tips** | **CFVs Cleaned** | **Washers** | **Gloves** | **Markin Clothes** |
| --- | --- | --- | --- | --- | --- | --- | --- | --- | --- | --- |
| **First Round 2015** | 272 | 168 | 99 | 92 | 103 | 188 | 17 | 49 | 73 | 178 |
| **Second Round 2015** | 49 | 64 | 39 | 41 | 14 | 73 | 142 | 28 | 47 | 123 |
| **First Round 2016** | 498 | 51 | 27 | 24 | 9 | 2 | 8 | 0 | 21 | 98 |
| **Second Round 2016** | 0 | 12 | 14 | 13 | 4 | 6 | 32 | 0 | 32 | 84 |
| **Total** | 819 | 295 | 179 | 170 | 130 | 269 | 199 | 77 | 173 | 483 |
