# Supplementary material for: Kala-azar elimination in a highly-endemic district of Bihar, India: A success story
Source: PLoS Negl Trop Dis. 2020 May 4;14(5):e0008254. doi: 10.1371/journal.pntd.0008254 (PMC7224556; doi:10.1371/journal.pntd.0008254)
Supplement: S10 Table — (DOCX) [file pntd.0008254.s015.docx]

**S10 Table: The impact of IEC/BCC and social mobilization on the VL control program assessed pre- and post-IRS in the Vaishali District, Bihar.**

| **Awareness survey pre-IRS 15 days** | | | | | | | |
| --- | --- | --- | --- | --- | --- | --- | --- |
| **IRS Round(s)** | **Villages Surveyed (n)** | **HHs Surveyed (n)** | **HHs Aware of VL and PKDL Cases and Its Symptoms (%)** | **HHs Aware of Government Facilities about VL and PKDL (%)** | **HHs Aware of VL Vector and Its Breeding and Resting Sites (%)** | **HHs Aware of VL Vector Control Intervention or IRS (%)** | **HHs Used Repellents (Mosquitoes Net, Coil, Ointment, and Cleanliness) for Vector Bites (%)** |
| **First Round 2015** | 256 | 7,680 | 1,173 (15.3%) | 1,519 (19.8%) | 687 (8.9%) | 3,283 (42.7%) | 2,412 (31.4%) |
| **Second Round 2015** | 256 | 7,680 | 3,758 (48.9%) | 4,012 (52.2%) | 3,361 (43.8%) | 4,178 (54.4%) | 2,627 (34.2%) |
| **First Round 2016** | 256 | 7,680 | 5,491 (71.5%) | 6,113 (79.6%) | 4,733 (61.6%) | 5,192 (67.6%) | 4,566 (59.5%) |
| **Second Round 2016** | 256 | 7,680 | 5,998 (78.1%) | 6,498 (84.6%) | 5,382 (70.1%) | 5,719 (74.5%) | 4,587 (59.7%) |
| **Average** | 256 | 7,680 | 4,105 (53.5%) | 4,535.5 (59.1%) | 3,540.8 (46.1%) | 4,593 (59.8%) | 3,548 (46.2%) |
| **Awareness Survey Post-IRS 1 Day** | | | | | | | |
| **First Round 2015** | 256 | 7,680 | 4,724 (61.5%) | 5,128 (66.8%) | 4,593 (59.8%) | 4,321 (56.3%) | 2,712 (35.3%) |
| **Second Round 2015** | 256 | 7,680 | 6,139 (79.9%) | 6,617 (86.2%) | 5,974 (77.8%) | 4,987 (64.9%) | 3,178 (41.4%) |
| **First Round 2016** | 256 | 7,680 | 6,354 (82.7%) | 6,921 (90.1%) | 6,304 (82.1%) | 5,429 (70.7%) | 4,721 (61.5%) |
| **Second Round 2016** | 256 | 7,680 | 6,441 (83.9%) | 7,158 (93.2%) | 6,428 (83.7%) | 6,459 (84.1%) | 5,281 (68.8%) |
| **Average** | 1024 | 30,720 | 5,914.5 (77%) | 6,456 (84.1%) | 5,824.8 (75.8%) | 5,299 (69%) | 3,859.3 (51.7%) |
