# Supplementary material for: Kala-azar elimination in a highly-endemic district of Bihar, India: A success story
Source: PLoS Negl Trop Dis. 2020 May 4;14(5):e0008254. doi: 10.1371/journal.pntd.0008254 (PMC7224556; doi:10.1371/journal.pntd.0008254)
Supplement: S11 Table — (DOCX) [file pntd.0008254.s016.docx]

**S11 Table: Survey report for HHs that received pre-IRS information from different sources, and the awareness developed about post-IRS activities in the Vaishali District, Bihar.**

| **IRS Round (S)** | **Total Villages Surveyed (n)** | **Total HHs Surveyed (n)** | **Total HHs Received Pre-IRS Information (%)** | **HHs Received Pre-IRS Information through Microphone Announcements** **and ASHAs (%)** | **HHs Received Pre-IRS Information through Microphone** **Announcements** **only (%)** | **HHs Received Pre-IRS Information through ASHAs Only (%)** | **HHs Received Pre-IRS Information through Other Sources (%)** | **HHs Refused IRS after Pre-IRS Information (%)** | **HHs Aware of post-IRS Activities (%)** |
| --- | --- | --- | --- | --- | --- | --- | --- | --- | --- |
| **First Round 2015** | 256 | 7,680 | 7,671 (99.9%) | 3,928 (51.2%) | 3,174 (41.4%) | 447 (5.8%) | 122 (1.6%) | 137 (1.8%) | 3,189 (41.5%) |
| **Second Round 2015** | 256 | 7,680 | 7,674 (99.9%) | 5,054 (65.9%) | 1,702 (22.2%) | 874 (11.4%) | 44 (0.6%) | 53 (0.7%) | 5,437 (70.8%) |
| **First Round 2016** | 256 | 7,680 | 7,680 (100%) | 5,209 (67.8%) | 1,498 (19.5%) | 906 (11.8%) | 67 (0.9%) | 32 (0.4%) | 6,012 (78.3%) |
| **Second Round 2016** | 256 | 7,680 | 7,679 (100%) | 5,548 (72.2%) | 1,284 (16.7%) | 758 (9.9%) | 89 (1.2%) | 37 (0.5%) | 5,887 (76.7%) |
| **Average** | 256 | 7,680 | 7,676 (99.9%) | 4,934.8 (64.3%) | 1,914.5 (24.9%) | 746.3 (9.7%) | 80.5 (1%) | 64.8 (0.8%) | 5,131.3 (66.8%) |
