# Supplementary material for: Kala-azar elimination in a highly-endemic district of Bihar, India: A success story
Source: PLoS Negl Trop Dis. 2020 May 4;14(5):e0008254. doi: 10.1371/journal.pntd.0008254 (PMC7224556; doi:10.1371/journal.pntd.0008254)
Supplement: S12 Table — (DOCX) [file pntd.0008254.s017.docx]

**S12 Table: Standardization of hand compression pump (HCP) for IRS in the Vaishali District, Bihar.**

| **No.** | **Characteristics** | **CSP** | **HCP** |
| --- | --- | --- | --- |
| **1** | **Quantity of suspension required** | 15 L/pump | 7.5 L/pump |
| **2** | **No. of strokes (one full bucket 15L solution for CSP and 7.5L one full pump solution for HCP)** | 23-27 | 55-58 |
| **3** | **Discharge rate without CFV** | 650-750 ml/m (average 700 ml/m) | 650-750 ml/m (average 700 ml/m) |
| **4** | **Discharge rate with CFV** | not applicable | 540-560 ml/m (average 550 ml/m) |
| **5** | **Persons per squad (SFW+FWs)** | 1+5 | 1+5 |
| **6** | **No. of pumps per squad** | 2 pumps | 3 pumps |
| **7** | **Persons required for each pump** | 2 | 1 |
| **8** | **Average HHs coverage per day/squad** | 40-50 HHs | 55-65 HHs |
| **9** | **Wastage of suspension** | 100-200ml for each pump with 15L of suspension | negligible |
| **10** | **Time required to spray 15/7.5 L of suspension.** | 30-40 min. | 13-14 min with CFV and 10-12 min without CFV |
| **11** | **Spray swath width required to maintain uniform spray on wall (with flat fan spray nozzle tips)** | 53 cm (without CFV; 8 cm overlapping between two consecutive swaths) | 75 cm (with CFV; 5 cm overlapping between two consecutive swaths) |
| **12** | **Distance of nozzle from wall** | 45 cm | 45 cm |
| **13** | **Spray timing to complete 6ft height with the standard swath width (downward to upward direction)** | 5-sec | 5-sec |
