# Supplementary material for: Kala-azar elimination in a highly-endemic district of Bihar, India: A success story
Source: PLoS Negl Trop Dis. 2020 May 4;14(5):e0008254. doi: 10.1371/journal.pntd.0008254 (PMC7224556; doi:10.1371/journal.pntd.0008254)
Supplement: S13 Table — (DOCX) [file pntd.0008254.s018.docx]

**S13 Table: Household based IRS survey for assessing the spray quality between HCPs and CSPs.**

| **Type of Pump** | **Total Villages Surveyed (n)** | **Total Sprayed HHs Visited (n)** | **HHs with Uniform Spray on Wall (%)** | **HHs with Patchy Spray on Wall (%)** | **Total Partially Sprayed HHs (%)** | **HHs Sprayed Partially due to Pump (%)** |
| --- | --- | --- | --- | --- | --- | --- |
| **Stirrup Pump** | 50 | 1,500 | 638 (42.5%) | 862 (57.5%) | 577 (38.5%) | 367 (50.1%) |
| **Compressor Pump** | 50 | 1,500 | 1492 (99.5%) | 8 (0.5%) | 131 (6.8%) | 0 (0.0%) |
