# Supplementary material for: Kala-azar elimination in a highly-endemic district of Bihar, India: A success story
Source: PLoS Negl Trop Dis. 2020 May 4;14(5):e0008254. doi: 10.1371/journal.pntd.0008254 (PMC7224556; doi:10.1371/journal.pntd.0008254)
Supplement: S14 Table — (DOCX) [file pntd.0008254.s019.docx]

**S4 Table. Insecticide susceptibility status of *P. argentipes* to DDT (4%) and SP (5%) assessed using the WHO-based tube method in the Vaishali District, Bihar, during 2015-2016.**

| **Year (s)** | **IRS Round (s)** | **Insecticide Test**  **(1h Exposure)** | **24 h Mortality Rate for Set-I (%)** | **24 h Mortality Rate for Set-II (%)** | **24 h Mortality Rate for Set-III (%)** | **24 h Mortality Rate for Set-IV (%)** | **Average Mortality Rate (%)** |
| --- | --- | --- | --- | --- | --- | --- | --- |
|  |  |  |  |  |  |  |  |
|  |  |  |  |  |  |  |  |
| **2015** | **First Round (Feb.-Apr.)** | DDT 4% | 47.1 | 48 | 40 | 55.6 | 47.7 |
|  | **Second Round (Jun.-Sept.)** | DDT 4% | 42.9 | 47.1 | 55 | 48 | 48.3 |
|  |  | SP 5% | 100 | 100 | 100 | 100 | 100 |
| **2016** | **First Round (Mar.-Jun.)** | SP 5% | 100 | 100 | 100 | 100 | 100 |
|  | **Second Round (Aug.-Nov.)** | SP 5% | 100 | 100 | 100 | 100 | 100 |
