# Supplementary material for: Kala-azar elimination in a highly-endemic district of Bihar, India: A success story
Source: PLoS Negl Trop Dis. 2020 May 4;14(5):e0008254. doi: 10.1371/journal.pntd.0008254 (PMC7224556; doi:10.1371/journal.pntd.0008254)
Supplement: S15 Table — The percent of change in post-IRS sand fly densities was calculated to assess the IRS intervention effect using DDT and SP. (DOCX) [file pntd.0008254.s020.docx]

**S15 Table: The results of *P. argentipes* densities caught in sprayed and unsprayed villages in pre- and post-IRS periods in the Vaishali District, Bihar, during 2015-2016. The percent of change in post-IRS sand fly densities was calculated to assess the IRS intervention effect using DDT and SP.**

| **Year (s)** | **IRS Round (s)** | **Number of LTs Installed** | **In Intervention Villages** | | | | **In Control Villages** | | | | **% of Change Post-IRS** | | |
| --- | --- | --- | --- | --- | --- | --- | --- | --- | --- | --- | --- | --- | --- |
|  |  |  | **Pre-IRS (7 Days)** | **Post-IRS (15 Days)** | **Post-IRS (1 Month)** | **Post-IRS (3 Months)** | **Pre-IRS (7 Days)** | **Post-IRS (15 days)** | **Post-IRS (1 Month)** | **Post-IRS (3 Months)** | **Post-IRS (15 Days)** | **Post-IRS (1 Month)** | **Post-IRS (3 Months)** |
|  |  |  |  |  |  |  |  |  |  |  |  |  |  |
| **2015** | **First Round (Feb-Apr)** | 96  (DDT-HHs) | (1-17) 2.0 | (1-6) 1.4 | (1-8) 1.7 | (1-8) 2.5 | (1-9) 2.3 | (1-8) 2.6 | (1-9) 2.1 | (1-7) 2.5 | -46.9 | -4.6 | 16.5 |
|  | **Second Round (Jun-Sept.)** | 42  (DDT-HHs) | (1-19) 3.7 | (1-6) 2.3 | (1-10) 3.4 | (1-7) 4.7 | (1-13) 3.3 | (2-14) 3.4 | (1-12) 3.6 | (1-14) 3.5 | -40.1 | -17.2 | 19.7 |
|  |  | 54  (SP-HHs) | (1-2016) 3.4 | (0-0) 0 | (0-2) 0.6 | (0-2) 2.3 | (1-14) 2.9 | (2-18) 2.7 | (1-8) 2.9 | (1-10) 3.0 | -94.6 | -84.2 | -38.6 |
| **2016** | **First Round (Mar-Jun)** | 96  (SP-HHs) | (1-12) 3.2 | (0-0) 0 | (0-1) 0.2 | (0-2) 1.7 | (2-17) 3.5 | (2-14) 3.2 | (1-12) 3.1 | (1-11) 3.4 | -90.8 | -81.5 | -43.9 |
|  | **Second Round (Aug-Nov)** | 96  (SP-HHs) | (1-14) 3.3 | (0-0) 0 | (0-2) 0.5 | (0-2) 1.8 | (2-13) 3.2 | (1-10) 2.9 | (1-2016) 3.3 | (2-12) 2.8 | -91.5 | -85.9 | -33.2 |
